# Phenotypic screening identifies kenpaullone as a prolymphangiogenic compound to improve heart repair following myocardial infarction

**DOI:** 10.1073/pnas.2619427123

**Published:** 2026-10-06

**Authors:** Christophe Ravaud, Caroline A. Cuoco, Sarah Sigal, Hannah M. Unsworth, Susanna T. E. Cooper, Adam B. Lokman, Carole J. R. Bataille, Angela J. Russell, Paul R. Riley

**Affiliations:** ^a^ Institute of Developmental and Regenerative Medicine, University of Oxford, Oxford OX3 7TY, United Kingdom; ^b^ Department of Physiology, Anatomy and Genetics, University of Oxford, Oxford OX1 3PT, United Kingdom; ^c^ Department of Pharmacology, University of Oxford, Oxford OX1 3QT, United Kingdom; ^d^ Chemistry Research Laboratory, Department of Chemistry, University of Oxford, Oxford OX1 3TA, United Kingdom

**Keywords:** phenotypic screening, small molecules, lymphangiogenesis, myocardial infarction

## Abstract

Myocardial infarction (MI) induces cardiac muscle death and its subsequent replacement by a noncontractile fibrotic scar, which underlies progression to heart failure. Current treatments restore blood flow and assist with cardiac workload but do not promote regeneration. We have previously shown endogenous growth of the cardiac lymphatic system following MI and further stimulation with the lymphatic endothelial specific mutated Vascular Endothelial Growth Factor-C (VEGF-C) isoform, VEGFC-C156S, resolves the immune response and improves cardiac function. However, the short half-life of VEGFC-C156S makes it suboptimal for clinical use, highlighting the need for alternative prolymphangiogenic strategies. We established a spheroid-based sprouting assay using human lymphatic endothelial cells to mimic lymphangiogenesis and performed a phenotypic screen of focused libraries of epigenetic regulators, kinase inhibitors, and stem cell modulators. Automated imaging and quantitative analysis identified several kinase inhibitors, previously characterized as GSK3ß inhibitors, as prolymphangiogenic regulators. Among them, the most potent was kenpaullone, which activated the same canonical prolymphangiogenic ERK pathway as VEGF-C, but induced a distinct transcriptional response in treated spheroids. Using kenpaullone-derived chemical probes and target-validation approaches, we identified MAP4K4 as a molecular target mediating the prolymphangiogenic effect observed. Finally, in a mouse MI model, kenpaullone enhanced cardiac lymphangiogenesis and improved cardiac function.

## Significance

Therapeutic lymphangiogenesis has emerged as a promising strategy to improve cardiac repair following myocardial infarction (MI). However, there remains an unmet need to identify pharmacological inducers of this process as potential future treatments. Here, we developed a scalable high-content phenotypic screening platform based on sprouting lymphatic spheroids to identify small molecule modulators of lymphangiogenesis. We identified kenpaullone, a known GSK3β inhibitor, as a potent inducer of lymphatic sprouting and demonstrated its effects were independent of Wnt-signaling and instead involved combined MAP4K4 inhibition and ERK activation. In vivo, kenpaullone enhanced cardiac lymphatic expansion and improved functional recovery in mice following MI. This study establishes a framework for the discovery of lymphangiogenic compounds and identifies kenpaullone as a potential therapeutic post-MI.

Cardiovascular disease is a leading cause of morbidity and mortality worldwide. Among them, myocardial infarction (MI) is a significant component, caused by a lack of blood flow and oxygen supply to the heart, often due to a narrowed or blocked coronary artery (1). Ensuing cardiomyocyte loss, inflammation, and fibrosis leads to remodeling and ultimately heart failure, a condition that is becoming increasingly prevalent worldwide (2).

Although current treatments improve survival, none of them adequately address the underlying tissue damage or promote effective cardiac repair. Several strategies have focused on stimulating cardiomyocyte proliferation, blocking cell death, or using exogenous progenitor/stem cells but these approaches have yielded limited success to date (3). More recently, the cardiac lymphatics have been identified as playing a key role in heart repair following MI, such that enhancing cardiac lymphangiogenesis post injury has emerged as a promising new therapeutic approach (4). Indeed, the delivery of the lymphatic specific Vascular Endothelial Growth Factor-C isoform [VEGF-C (C156S)] in a MI murine model stimulated cardiac lymphangiogenesis via the Vascular Endothelial Growth Factor Receptor 3 (VEGFR-3), reduced fibrosis, remodeling and improved cardiac function in the injured heart (5, 6). In a rat MI model, this treatment significantly lowered cardiac water content and edema (7) and in mice, VEGF-C (C156S)–induced lymphatics enhanced the removal of infiltrating immune cells, helping to resolve inflammation (6). Interestingly, this effect was absent in Lymphatic Vessel Endothelial hyaluronan receptor 1 (LYVE-1) knockout mice, where lymphatics can no longer dock and traffic immune cells, demonstrating the importance of newly formed cardiac lymphatic vessels in resolving inflammation (6, 8, 9).

The VEGF-C/VEGFR-3 signaling axis controls lymphangiogenesis. The absence of either VEGF-C or VEGFR3 in mice leads to severe defects in the formation of lymphatic vessels (10). VEGF-C binds to VEGFR-3 and induces receptor dimerization, triggering its autophosphorylation which in turn activates several downstream pathways, notably PI3K/AKT and ERK (11). In vitro studies reveal that the PI3K/AKT pathway mediates lymphatic endothelial cell (LEC) tube formation and migration (12) and the ERK/MAPK pathway promotes LEC survival and sprouting (13).

Despite the promising therapeutic effects of VEGF-C in promoting improved outcome post-MI translating to patients faces significant practical limitations, including cost, delivery complexity, short half-life (14), and a broad target profile, making it suboptimal for clinical use. Thus, new modulators of lymphangiogenesis are desirable to fill this therapeutic gap, and, to date, small-molecule drug-like inducers remain largely unexplored.

To identify such modulators, several in vitro assays using LECs have been adapted including simple and cost-effective 2D monolayer cultures (15) employed in Boyden chamber and wound scratch assays are to assess inducers of cell migration and proliferation. More recently, LEC cultures in flasks coated with extracellular matrix proteins (16) have been established to induce tubulogenesis using a tube formation assay (or plexus assay), in a Matrigel or Collagen matrix, to model early lymphatic capillary network formation. However, this method is limited to 2D cell behavior inferences and as such remains somewhat artificial and unable to model more complex biological processes.

Alternatively, the 3D spheroid sprouting assay allows for assessment of cell sprouting from LEC aggregates in a 3D type I collagen matrix. This assay is more physiologically relevant, as it involves extracellular matrix degradation, cell migration, proliferation, and lumen formation (17) and provides a robust, quantifiable platform for high-throughput screening of prolymphangiogenic modulators. The hanging drop (HD) method enables the formation of uniform spheroids by culturing cells in suspended droplets and is suitable for studying the effects of genetic manipulation or drug treatment on (lymph)angiogenesis (17). However, this approach is laborious and low-throughput, restricting its application for large-scale screening. To overcome these limitations, we adapted this assay using the AggreWell plates, enabling the formation of highly uniform LEC 3D spheroids on a larger scale. We miniaturized the system into a 384-well plate format, making it compatible with automated handling and high-content imaging, while preserving the biological complexity and relevance of the original 3D sprouting model.

Using this assay, we screened over 350 compounds with defined pharmacology and identified several compound classes with significant prosprouting activity. One such class identified to induce sprouting was inhibitors of the serine/threonine kinase glycogen synthase kinase-3β (GSK3β). Among them, kenpaullone was selected as the lead candidate due to its robust induction of spheroid sprouting in our screen. Kenpaullone is a well-characterized small molecule and classically associated with GSK3β/Wnt signaling, although it also targets other kinases such as CDKs and MAP4K4 (18, 19). Here, we show that kenpaullone promotes lymphatic endothelial sprouting independently of the GSK3β/Wnt pathway, instead acting via MAP4K4 inhibition and ERK activation. Finally, we demonstrate that Kenpaullone enhances cardiac lymphangiogenesis and improves cardiac repair post-MI in mice, highlighting the utility of our screening platform to find potential therapeutic candidates in the context of myocardial infarction.

## Results

**Fig. 1.**
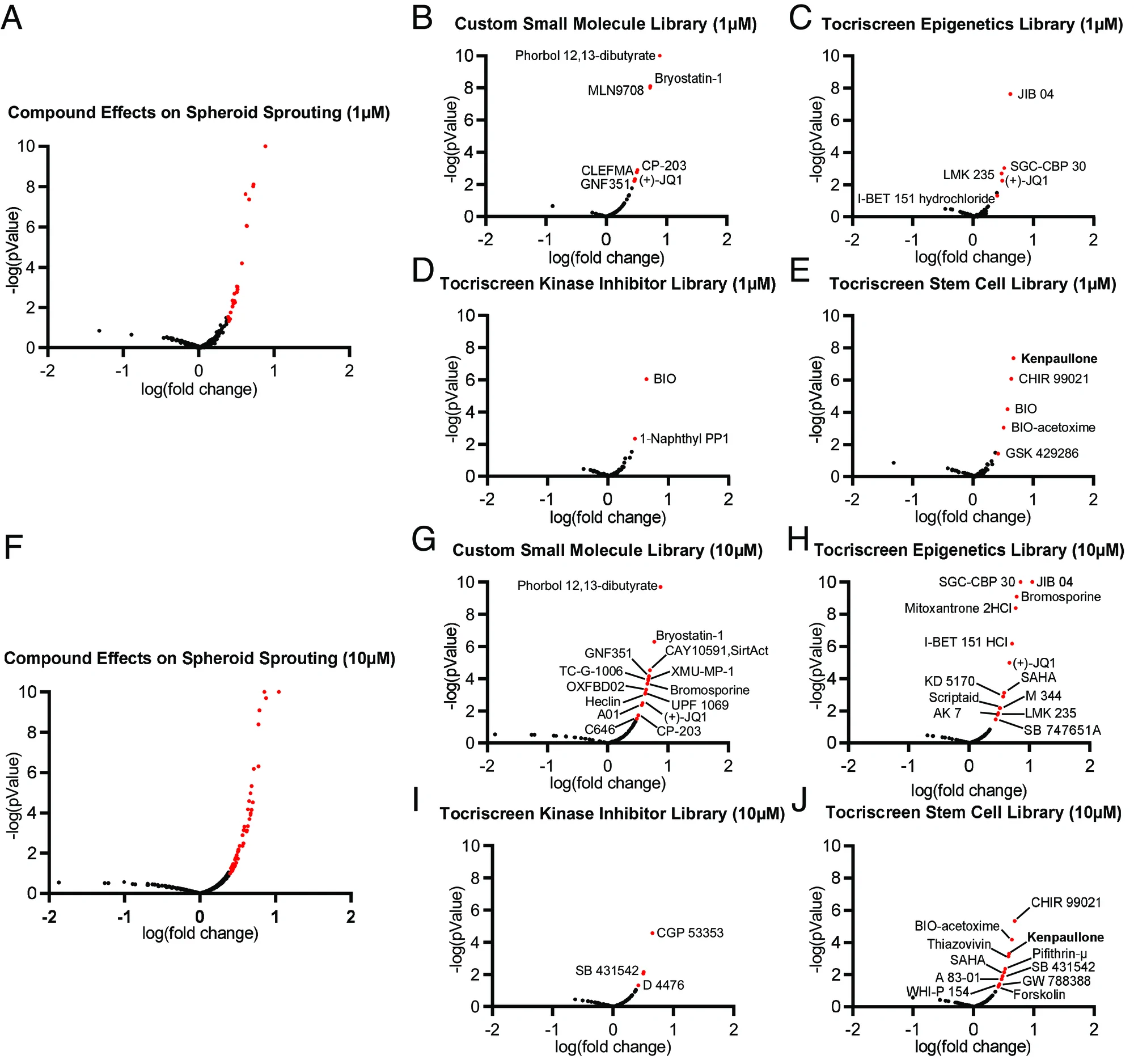
Volcano plot of small-molecule screening for spheroid sprouting. (*A*) Volcano plot of all compounds tested at 1 µM. (*B*–*E*) Volcano plots of individual libraries at 1 µM: (*B*) custom small library, (*C*) epigenetics modulators, (*D*) kinase inhibitors, (*E*) stem cell modulators. (*F*) Volcano plot of all compounds tested at 10 µM. (*G*–*J*) Volcano plots of individual libraries at 1 µM: (*G*) custom small library, (*H*) epigenetics modulators, (*I*) kinase inhibitors, (*J*) stem cell modulators. For each compound, the mean log2 fold-change in sprout number relative to vehicle, DMSO, (*x*-axis) is plotted against log10(*P*-value) (*y*-axis). Positive hits (red) were defined as compounds with a fold change > 2.5 and *P* < 0.05 and are annotated on *B*–*E* and *G*–*J*. n = 3, One-way ANOVA followed by Two-stage linear step-up procedure of Benjamini, Krieger, and Yekutieli.

**Fig. 2.**
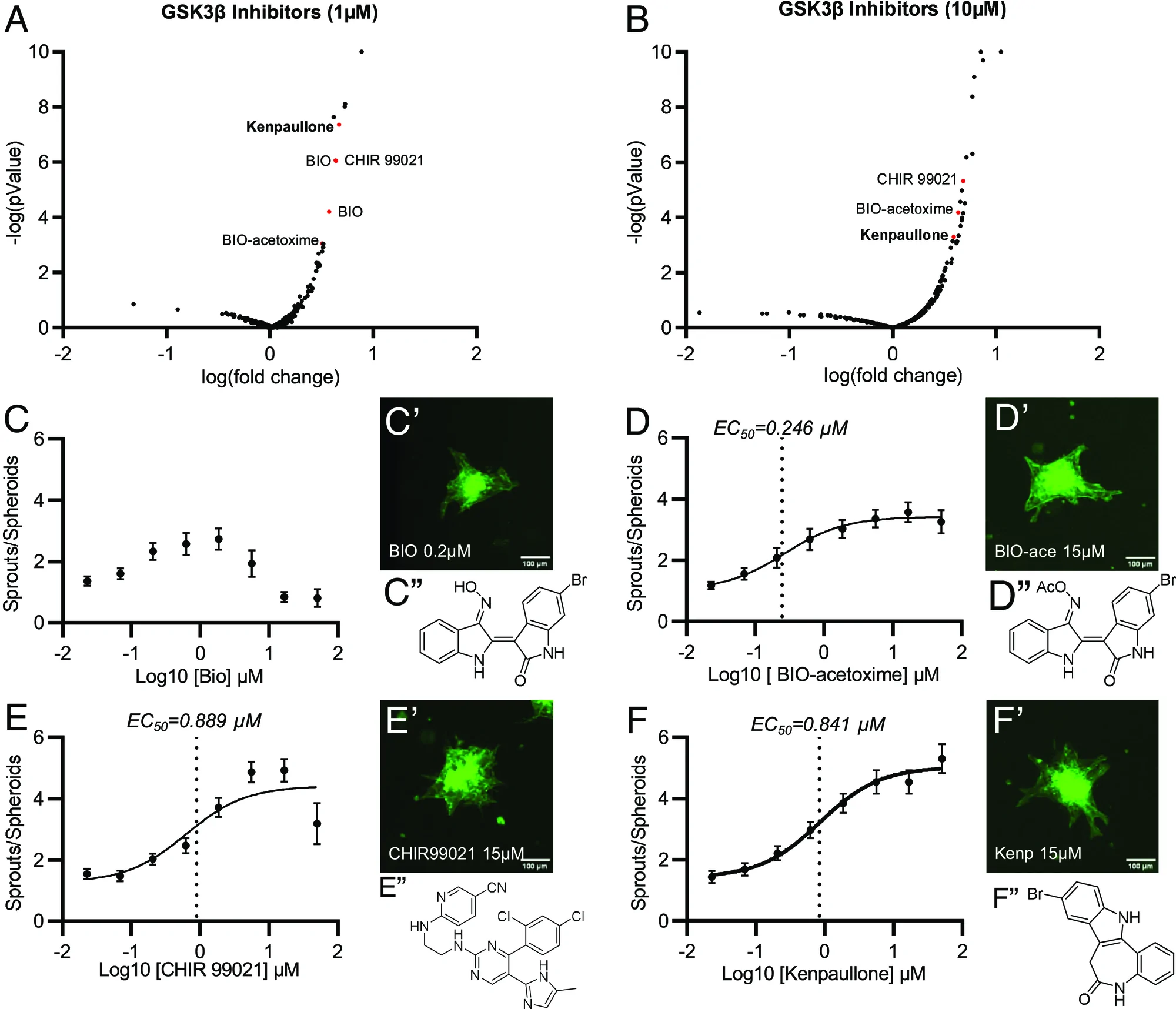
GSK3β inhibitors induce LEC sprouting in a dose-dependent manner. (*A* and *B*) Volcano plots of the compound screen at 1 µm (*A*) and 10 µm (*B*) with GSK3β inhibitors annotated. (*C*–*F*) Dose–response curves of BIO (*C*), BIO-acetoxime (*D*), CHIR 99021 (*E*), and Kenpaullone (*F*) with their corresponding calculated EC_50_ values, representative spheroid images at a given concentration (*C’*–*F’*) and chemical structures (*C’’*–*F’*’). Data are shown as mean ± SEM, n = 10 to 13 independent experiments. (Scale bar: 100 µM.)

Primary human LEC spheroids were embedded in a 3D collagen type I matrix and imaged after 24 h of treatment (*SI Appendix*, Fig. S1*A*). VEGF-C (50 ng/mL) treatment, a known prolymphangiogenic factor, significantly increased sprout number compared to control (vehicle treated) demonstrating the sensitivity of the assay (*SI Appendix*, Fig. S1 *B* and *C*). To enable robust and unbiased quantification of lymphatic endothelial cell (LEC) sprouting, we developed a high-throughput image analysis pipeline using CellProfiler. The pipeline was designed to identify individual spheroids by applying fluorescence intensity threshold to detect spheroid cores and to quantify individual sprouts by subtracting the cores to the masked image, thereby isolating the sprouting structures (*SI Appendix*, Fig. S1*D*). Manual and CellProfiler quantification under VEGF-C dose response gave similar results, with a significant Spearman correlation test (r = 0.9762, *P* = 0.004), demonstrating the sensitivity and specificity of the automated analysis (*SI Appendix*, Fig. S1*E*). In addition, this assay proved to be reproducible inter- and intraplates, across technical replicates, with low variability and consistent signal-to-noise ratio, making it suitable for large-scale screening. We then conducted a screen of 350 small molecules from commercially available libraries targeting epigenetic regulators, kinases, and stem cell modulators along with a custom-made compound set. The sprout to spheroid ratio was normalized to vehicle-treated control for each plate, and data were visualized using volcano plots to identify compounds that significantly enhanced LEC sprouting (Fig. 1). A fold change threshold of 2.5 and statistical significance cut-off of *P* < 0.05 were used to define positive hits, highlighted in red at both 1 µM and 10 µM concentrations on the volcano plots (Fig. 1 *A* and *F*). Each library was analyzed separately for each concentration and the compound ID for each positive hit was shown (Fig. 1 *B*–*E* and *G*–*J*). Thus, 20 and 48 compounds at 1 µM and 10 µM, respectively, were reported with their fold change and *P-*values in Table 1. Notably, several overlapping hits were independently identified, such as (+)-JQ1 (Fig. 1 *B*, *C*, *G*, and *H*), Bio (Fig. 1 *D* and *E*), and SB 431542 (Fig. 1 *I* and *J*), highlighting the robustness and reproducibility of the assay. The remaining list of the compounds, along with their average fold change and *P*-value is provided in *SI Appendix*, Tables S1 and S2. To further characterize the selected hits, dose–response analyses were performed across a concentration range of 23 nM to 50 µM (*SI Appendix*, Fig. S2 *C*–*F*). Several compound classes emerged as potential inducers of lymphangiogenic sprouting, including PKC modulators, bromodomain inhibitors, HDAC inhibitors, (*SI Appendix*, Fig. S2 *B*–*D*) and notably GSK3β inhibitors (Fig. 2). Given that GSK3β is a well-known regulator of multiple signaling pathways, including Wnt/β-catenin (20), and has been implicated in vascular development and angiogenesis (21), we focused on hit molecules known to act as GSK3β inhibitors. Four independent compounds, all with GSK3β as a common target were identified as hits in the screen at 1 µM (Fig. 2*A*): Bio, Bio-acetoxime, CHIR 99021, and Kenpaullone. All of these compounds induced sprouting at 10 µM except for Bio (Fig. 2*B*). Bio and Bio-acetoxime share a nearly identical chemical structure and are derived from indirubins (22) (Fig. 2 *C”* and *D”*), whereas CHIR99021 is an aminopyrimidine derivative (Fig. 2*E”*) and kenpaullone a paullone derivative (Fig. 2*E”*) (18, 23, 24). Even though these small molecules are structurally distinct, they are all ATP-competitive Type I inhibitors, binding to the same site on GSK3β as ATP, effectively blocking the enzymatic activity. Dose–response curves were generated to further validate and assist in prioritizing the most active GSK3β inhibitor for further studies. Bio displayed a bell-shaped curve (Fig. 2*C*), indicating reduced activity at higher concentrations, consistent with the findings at 10 µM dose. In contrast, sigmoidal curves were obtained with the alternative GSK3β inhibitors and the half maximal effective concentration (EC_50_) was calculated for Bio-Acetoxime (Fig. 2*D*; EC_50_ = 0.246 µM), CHIR 99021 (Fig. 2*E*; EC_50_ = 0.889 µM), and kenpaullone (Fig. 2*F*; EC_50_ = 0.841 µM). Representative images of sprouted spheroids are shown for each treatment (Fig. 2 *C’*–*F’*). CHIR 99021 revealed reduced efficacy at the highest concentration (50 µM), suggesting potential toxicity (Fig. 2*E*). Kenpaullone induced robust sprouting, with a higher fold-change than Bio-acetoxime, without evidence of toxicity at high concentrations. Based on its potency, efficacy, and favorable profile, alongside previous insights into toxicity, tolerability, and structure–activity relationships (SAR) (25–27) kenpaullone was selected for further investigation. To determine whether kenpaullone effects were specific to LECs, a similar dose–response analysis was performed using human umbilical vein endothelial cell (HUVEC) spheroids. Although HUVEC spheroids displayed higher basal sprouting activity, kenpaullone resulted in only a modest increase in sprouting above baseline, suggesting its prosprouting activity is more targeted toward LECs (*SI Appendix*, Fig. S3).

**Table 1.** Positive hits identified at 1 µM and 10 µM in the 3D LEC sprouting assay

Compounds	Libraries	1 µM fold	10 µM fold
GSK 429286	Tocris StemCells Library	2.600/*P* = 0.037	NS
1-Naphthyl PP1	Tocris Kinase Library	2.819/*P* = 0.005	NS
CLEFMA	Custom Library	3.204/*P* = 0.002	NS
BIO	Tocris StemCells Library	3.755/*P* = 6.4*10^−5^	NS
BIO	Tocris Kinase Library	4.363/*P* = 9.0*10^−7^	NS
MLN9708	Custom Library	5.312/*P* = 1.0*10^−10^	NS
D 4476	Tocris Kinase Library	NS	2.641/*P* = 0.047
WHI-P 154	Tocris StemCells Library	NS	2.641/*P* = 0.047
GW 788388	Tocris StemCells Library	NS	2.705/*P* = 0.039
6,2′,4′-Trimethoxyflavone	Custom Library	NS	2.721/*P* = 0.064
SB 747651A dihydrochloride	Tocris Epigenetic Library	NS	2.755/*P* = 0.034
*VEGFA* 50 ng/mL	*Custom Library*	3.330/*P* = 0.001	2.789/*P* = 0.054
PF 670462	Custom Library	NS	2.836/*P* = 0.048
MK-8617	Custom Library	NS	2.862/*P* = 0.045
AMG 900	Custom Library	NS	2.880/*P* = 0.043
SirReal2	Custom Library	NS	2.892/*P* = 0.042
A 83-01	Tocris StemCells Library	NS	2.933/*P* = 0.019
LMK 235	Tocris Epigenetic Library	2.975/*P* = 0.002	2.969/*P* = 0.017
C646	Custom Library	NS	3.019/*P* = 0.030
AK 7	Tocris Epigenetic Library	NS	3.032/*P* = 0.014
*VEGFC 50 ng/mL*	*Custom Library*	2.926/*P* = 0.006	3.049/*P* = 0.028
SB 431542	Tocris StemCells Library	NS	3.059/*P* = 0.013
SB 431542	Tocris Kinase Library	NS	3.174/*P* = 0.009
CP-203	Custom Library	3.278/*P* = 0.001	3.189/*P* = 0.019
SAHA	Tocris StemCells Library	NS	3.225/*P* = 0.007
SB 203580 hydrochloride	Tocris Kinase Library	NS	3.235/*P* = 0.007
Scriptaid	Tocris Epigenetic Library	NS	3.243/*P* = 0.007
M 344	Tocris Epigenetic Library	NS	3.245/*P* = 0.007
Pifithrin-μ	Tocris StemCells Library	NS	3.357/*P* = 0.004
KD 5170	Tocris Epigenetic Library	NS	3.668/*P* = 0.001
A01	Custom Library	NS	3.677/*P* = 0.004
(+)-JQ1	Custom Library	2.981/*P* = 0.005	3.798/*P* = 0.003
SAHA	Tocris Epigenetic Library	NS	3.798/*P* = 0.001
Thiazovivin	Tocris StemCells Library	NS	3.805/*P* = 0.00
Kenpaullone	Tocris StemCells Library	4.679/*P* = 4.4*10^−8^	3.889/*P* = 5.0*10^−4^
Heclin	Custom Library	NS	4.152/*P* = 0.001
UPF 1069 (IG-4D-49)	Custom Library	NS	4.195/*P* = 0.001
BIO-acetoxime	Tocris StemCells Library	3.224/*P* = 0.001	4.315/*P* = 6.6*10^−5^
OXFBD02	Custom Library	NS	4.322/*P* = 4.7*10^−4^
CGP 53353	Tocris Kinase Library	NS	4.494/*P* = 2.7*10^-5^
Bromosporine	Custom Library	NS	4.539/*P* = 2.0*10^−4^
TC-G-1006	Custom Library	2.654/*P* = 0.017	4.667/*P* = 1.2*10^−4^
(+)-JQ1	Tocris Epigenetic Library	3.049/*P* = 0.006	4.668/*P* = 1.1*10^−5^
XMU-MP-1	Custom Library	NS	4.714/*P* = 1.0*10^−4^
GNF351	Custom Library	2.913/*P* = 0.006	4.810/*P* = 7.0*10^−5^
CHIR 99021	Tocris StemCells Library	4.329/*P* = 8.7*10^−7^	4.814/*P* = 4.7*10^−6^
CAY10591,SirtAct	Custom Library	NS	5.016/*P* = 3.0*10^−5^
I-BET 151 hydrochloride	Tocris Epigenetic Library	2.530/*P* = 0.048	5.151/*P* = 6.6*10^−7^
Mitoxantrone dihydrochloride	Tocris Epigenetic Library	NS	5.934/*P* = 4.1*10^−9^
Bryostatin-1	Custom Library	5.347/*P* = 7.7*10^−9^	5.942/*P* = 5.0*10^−7^
Bromosporine	Tocris Epigenetic Library	NS	6.159/*P* = 8.0*10^−10^
SGC-CBP 30	Tocris Epigenetic Library	3.279/*P* = 0.001	7.100/*P* = 1.0*10^−10^
Phorbol 12,13-dibutyrate	Custom Library	7.720/*P* = 1.0*10^−10^	7.495/*P* = 2.0*10^−10^
JIB 04	Tocris Epigenetic Library	4.174/*P* = 2.3*10^−8^	11.14/*P* = 1.0*10^−10^

Compounds were considered hits based on a fold-change >2.5 and *P* < 0.05 compared to vehicle control. Data are presented as mean fold change from three independent experiments. NS: No significant effect.

Given that kenpaullone is a known GSK3β inhibitor, we first determined the relationship between prolymphangiogenic effects in LEC spheroids and canonical GSK3β inhibition-dependent Wnt signaling. The canonical Wnt pathway is regulated by the stabilization and nuclear translocation of β-catenin which drives the transcription of Wnt target genes. Under resting condition, β-catenin is phosphorylated by GSK3β leading to its ubiquitination and proteasomal degradation, thereby preventing Wnt pathway activation (28). Inhibition of GSK3β, by small molecules like Kenpaullone, impairs this process, allowing β-catenin to accumulate and translocate to the nucleus, thereby activating canonical Wnt signaling downstream targets. We first confirmed that β-catenin protein levels were increased in kenpaullone treated cells and that it was translocated into the nucleus, indicating activation of the Wnt pathway (*SI Appendix*, Fig. S4 *A*–*C*). To assess if Wnt pathway signaling played a role in LEC sprouting, spheroids were treated with a dose range, from 91 pg/mL to 200 ng/mL, of various human Wnt recombinant proteins, including Wnt3a, Wnt5a, and Wnt5b. Interestingly, none of these proteins promoted sprouting (*SI Appendix*, Fig. S4*D*). Furthermore, Wnt pathway activators that act independently of GSK3β inhibition, such as AMBMP (29), SKL2001 (30), or WAY 316606 (31), also failed to enhance sprouting (*SI Appendix*, Fig. S4*E*). These results suggest that Wnt signaling activation alone is neither sufficient nor required for LEC spheroids sprouting in this context. To investigate if kenpaullone’s activity, specifically its inhibitory activity of GSK3β, could account for the observed effects, we synthesized two structural analogues based on previously published SAR (18, 23, 24). Kenpaullone Analogue A (KP-An-A) lacks the 9-bromo group, disrupting kinase binding and reducing its GSK3β inhibition potency [IC_50_ = 0.620 µM versus 0.023 µM for kenpaullone previously reported (23)]. Kenpaullone Analogue B (KP-An-B), lacking the 9-bromo group and replacing the lactam with a carbocycle, completely loses kinase binding activity [IC_50_ > 1,000 µM previously reported (23)] (*SI Appendix*, Fig. S5*A*). Dose–response analysis revealed that the less potent kinase inhibitor showed reduced potency in inducing LEC sprouting, and the inactive analogue completely ablated sprouting, supporting a functional correlation between kinase targeting and biological effect (*SI Appendix*, Fig. S5*B*). Contrary to the previously described GKS3β ATP-competitive inhibitors, Compound 1 (*SI Appendix*, Fig. S5*C*), a GSK3β allosteric inhibitor (32), failed to stimulate sprouting (*SI Appendix*, Fig. S5*D*), suggesting that direct inhibition of GSK3β alone is not responsible for LEC sprouting. To further support that GSK3β inhibition itself was not accountable for kenpaullone-induced sprouting, we genetically knocked down GSK3β with two independent siRNAs. Both mRNA and protein levels of GSK3β were efficiently reduced in our LEC model (*SI Appendix*, Fig. S5 *E*–*G*). However, GSK3β-deficient LEC spheroids did not exhibit enhanced sprouting (*SI Appendix*, Fig. S5*H*), further supporting that kenpaullone’s prolymphangiogenic effect is not mediated through GSK3β inhibition. Taken together, these findings demonstrate that kenpaullone induced lymphangiogenesis independently of both GSK3β inhibition and Wnt pathway activation.

**Fig. 3.**
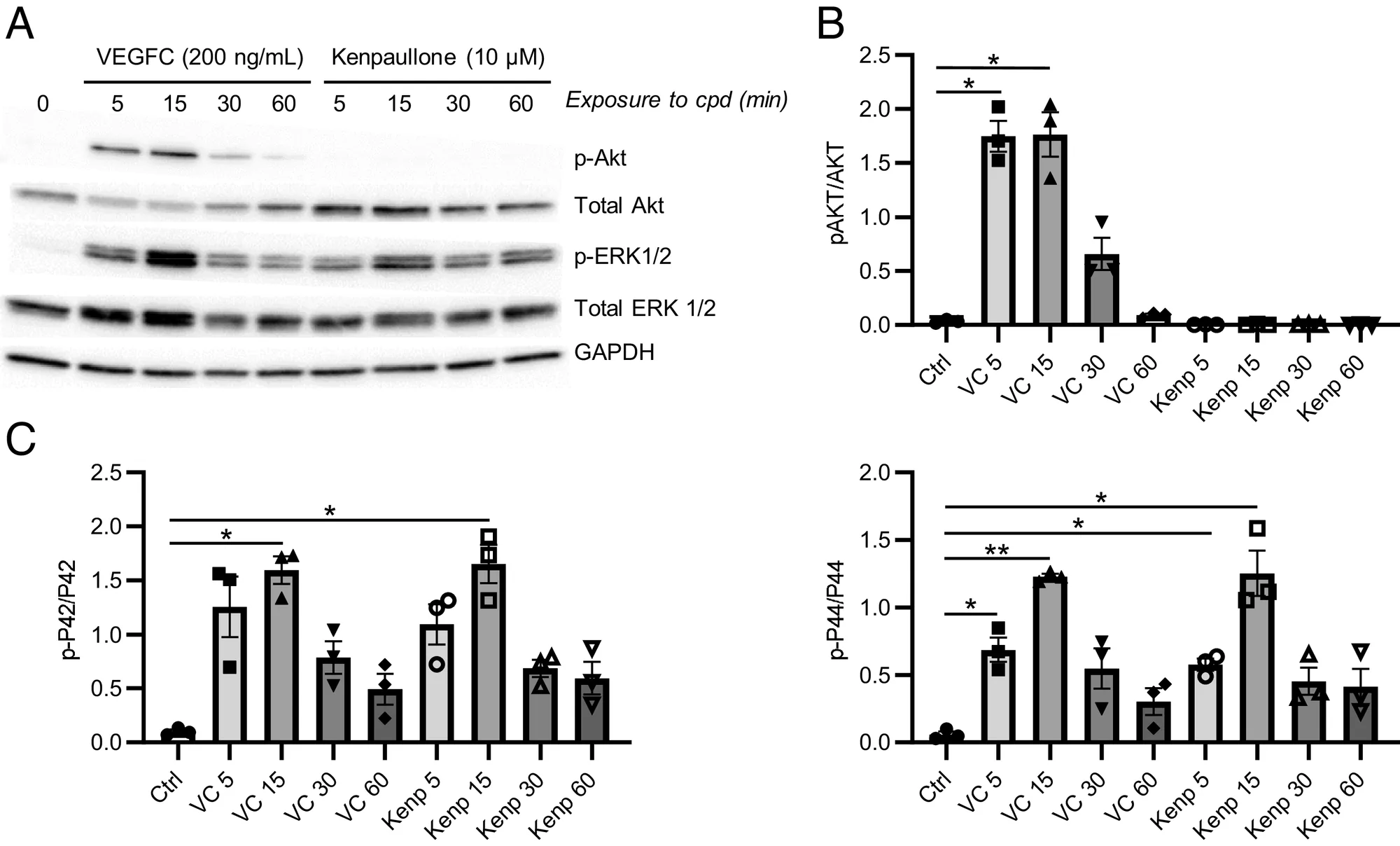
Kenpaullone selectively activates the prolymphangiogenic ERK pathway. (*A*) Representative western blot of phosphorylated Akt (p-AKT) and phosphorylated ERK1/2 (p-ERK1/2) with their respective total form on serum-starved LEC stimulated with VEGFC (200 ng/mL) or Kenpaullone (10 µM) for the indicated exposure time. GAPDH was used as a loading control. (*B* and *C*) Quantification analysis of AKT and ERK1/2 phosphorylation normalized to total AKT and ERK levels, respectively. Data are shown as mean ± SEM, n = 3. One-way ANOVA followed by Dunnett’s multiple comparison test versus DMSO control *P < 0.05, **P < 0.01.

To explore potential downstream effectors, we next focused on pathways commonly activated by VEGF-C-VEGFR3 signaling in LEC, particularly ERK/MAPK and PI3K/AKT (33). Both pathways are known to be critical for lymphangiogenesis and thus we investigated whether kenpaullone might exert its effects through similar mechanisms. To this end, we compared the activation kinetics of the ERK and AKT pathways in response to kenpaullone or VEGF-C stimulation over a time course (0, 5, 15, 30, and 60 min). Western Blot analysis revealed that VEGF-C rapidly induced phosphorylation of AKT, with levels increasing from 5 min, peaking at 15 min, and progressively declining thereafter (Fig. 3 *A* and *B*). ERK phosphorylation followed a similar kinetic profile (Fig. 3 *A* and *C*), consistent with previously reported VEGFR-3 downstream signaling (33). In contrast, kenpaullone treatment did not induce AKT phosphorylation at any time point (Fig. 3 *A* and *B*). However, ERK phosphorylation was robustly activated, displaying a similar profile to VEGF-C (Fig. 3 *A* and *C*). These results suggest that kenpaullone selectively activates the ERK/MAPK pathway without engaging PI3K/AKT signaling, indicating a distinct but partially overlapping mechanism with VEGF-C in promoting lymphangiogenesis.

**Fig. 4.**
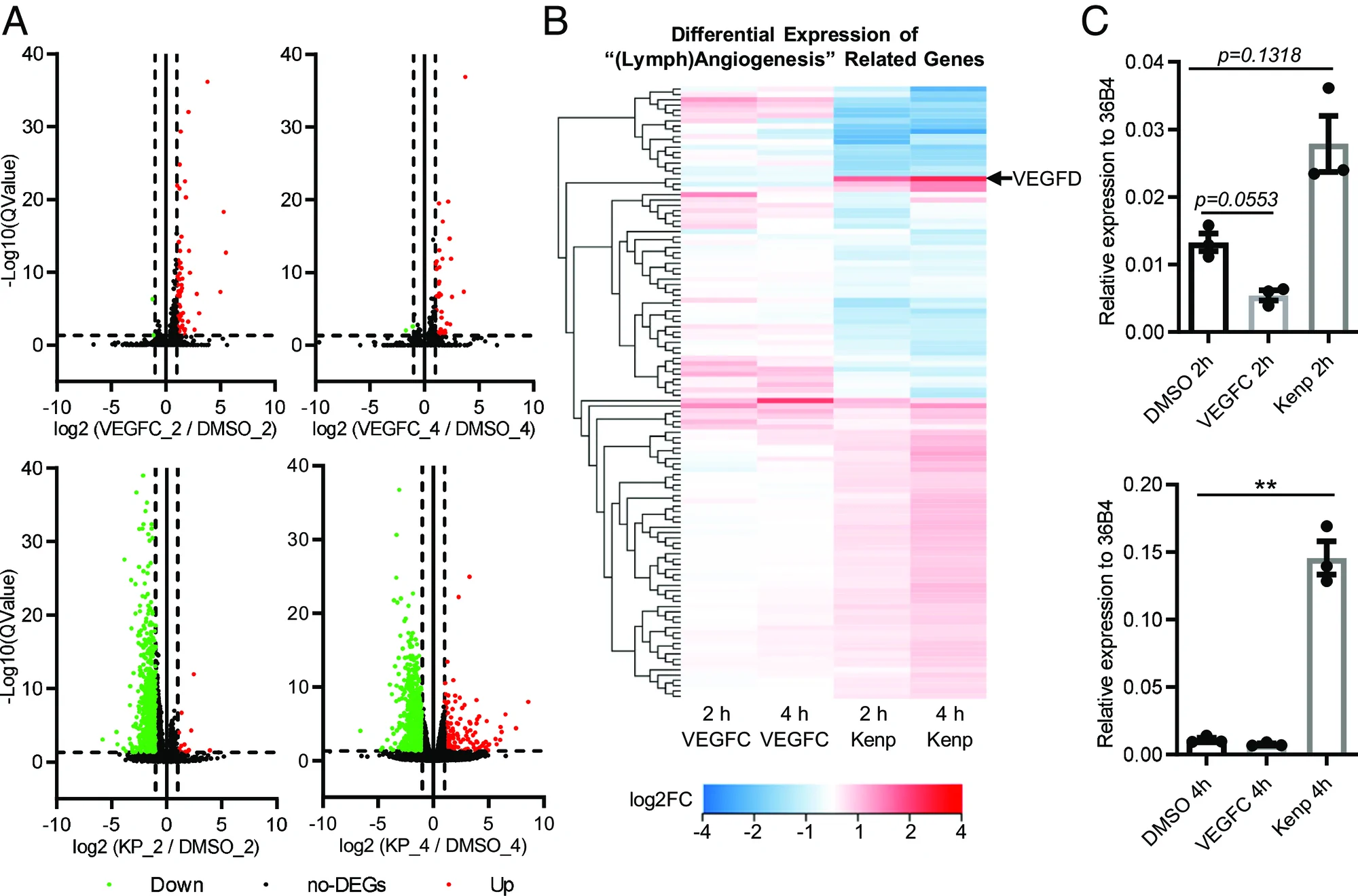
Bulk RNA-Seq reveals distinct transcriptomic response to Kenpaullone compared to VEGFC. (*A*) Volcano plots of differentially expressed genes (DEGs) in LEC spheroids treated with VEGF-C (200 ng/mL) or Kenpaullone (10 µM) compared to vehicle (DMSO) at 2 h and 4 h posttreatment. Red and green dots indicate significantly up- and down-regulated genes, respectively (q-value <0.05 and log2 fold change >1). n = 3 replicates per condition. (*B*) Heatmap of DEGs related to angiogenesis and lymphangiogenesis GO terms. (*C*) qPCR analysis of VEGFD expression following 2 h and 4 h LEC spheroid treatment. Data are shown as mean ± SEM, n = 3. One-way ANOVA followed by Dunnett’s multiple comparison test versus DMSO control **P < 0.01.

To further explore the molecular mechanisms underlying the distinct signaling responses induced by kenpaullone and VEGF-C, we performed bulk RNA sequencing on LEC spheroids treated with VEGF-C, kenpaullone, or DMSO for 2 and 4 h. We chose these time points to coincide with just prior to and at the onset of the initiation of sprouting, typically observed by 4 h post treatment. Differentially expressed genes (DEG) were visualized using volcano plots, comparing treated samples to DMSO controls at each time point (Fig. 4*A*). DEGs were defined as genes with a *q*-value <0.05 and absolute log2 fold change >1, corresponding to a minimum twofold change in expression. The transcriptional responses to VEGF-C and kenpaullone were markedly different and clearly distinguishable in the volcano plots, underscoring divergent transcriptional programs triggered by each treatment. At 2 h and 4 h, VEGF-C treatment led to 65 DEGs (63 upregulated and two downregulated) and 45 DEGs (43 upregulated and two downregulated), respectively. In contrast, kenpaullone induced a far broader transcriptional shift with 807 DEGs (14 upregulated and 793 downregulated) at 2 h and 1,283 DEGs (212 upregulated and 1,071 downregulated) at 4 h (Fig. 4*A*). Direct comparison of kenpaullone and VEGF-C treatments further highlighted their divergence, revealing 1,001 (43 upregulated and 958 downregulated) at 2 h and 1,458 (299 upregulated and 1,159 downregulated) at 4 h suggesting that kenpaullone engages a distinct regulatory program. Given the broad kinase target profile of kenpaullone, it is likely that a substantial fraction of these transcriptional changes reflects off-target effects. To gain further insight into the signaling pathways modulated by VEGF-C and kenpaullone, we performed gene ontology (GO) biological process enrichment analysis (*SI Appendix*, Fig. S6). In VEGF-C-treated spheroids, several GO terms related to (lymph)angiogenesis emerged, including positive regulation of cell migration, cellular response to vascular endothelial growth stimulus, cell junction assembly, and endothelial cell chemotaxis. In contrast, kenpaullone-treated spheroids led to global transcriptional and translational changes, with enriched GO terms including SRP-dependent cotranslational protein targeting to membrane, protein ubiquitination, cytoplasmic translation, chromatin organization, and translation initiation among others. We next focused on gene sets related to angiogenesis and lymphangiogenesis in our RNA-seq data and generated heatmaps to examine differential gene expression patterns (Fig. 4*B*). Remarkably, Kenpaullone resulted in both upregulation and downregulation of numerous (lymph)angiogenesis genes compared to VEGF-C treatment. Among them, VEGF-D, a known lymphangiogenic factor (34), was significantly upregulated following kenpaullone treatment, and this was confirmed by qPCR (Fig. 4*C*). Altogether, these data support the fact that kenpaullone acts to induce lymphangiogenesis via a distinct pathway(s) as compared to VEGF-C which in turn induces distinct transcriptional signature in LECs.

**Fig. 5.**
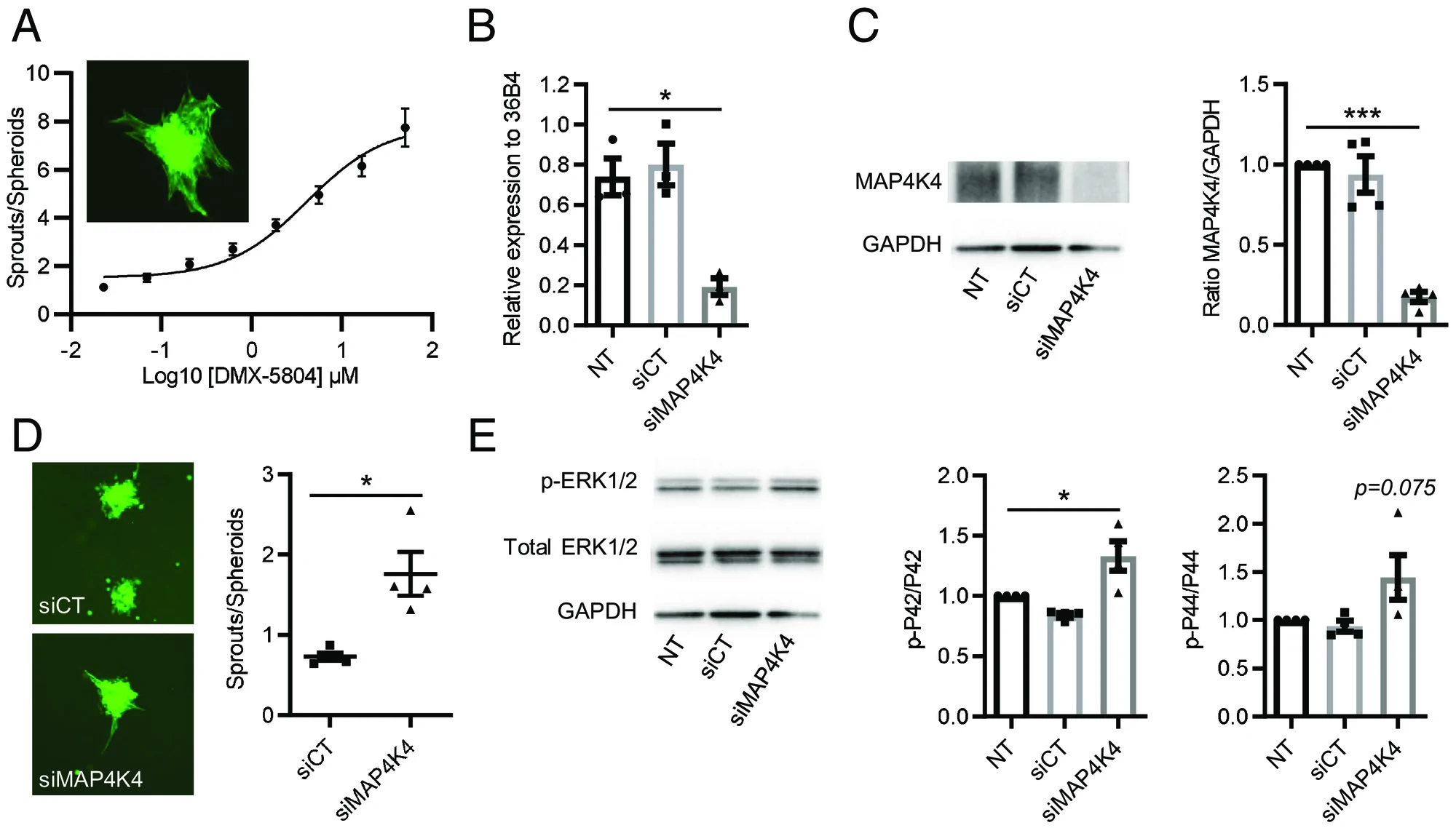
Kenpaullone promotes LEC sprouting through MAP4K4 inhibition. (*A*) Dose–response curve analysis of DMX5804 on LEC spheroid sprouting. A representative image of a spheroid sprouting under DMX5804 is shown (10µM). Data shown as mean ± SEM, n = 7. (*B*) qPCR analysis of LEC transfected with siRNA targeting MAP4K4. Data shown as mean ± SEM, n = 4. One-way ANOVA followed by Dunnett’s multiple comparison test versus NT *P < 0.05. (*C*) Representative Western Blot of MAP4K4 levels 2 d post LEC transfection. Quantification analysis of MAP4K4 expression normalized to loading control. Data are shown as mean ± SEM, n = 4. One-way ANOVA followed by Dunnett’s multiple comparison test versus NT ***P < 0.001. (*D*) Spheroids sprouting assay following MAP4K4 silencing with representative spheroid images. Data shown as mean ± SEM, n = 4, paired *t* test, *P < 0.05. (*E*) Representative Western Blot of p-ERK1/2 and ERK1/2 2 d post LEC transfection with siRNA targeting MAP4K4. The GAPDH loading control shown in this panel is the same shown in (*C*), as the membrane was stripped and reprobed for the indicated proteins. Quantification analysis of p-ERK1/2 normalized to total ERK. Data are shown as mean ± SEM, n = 4. One-way ANOVA followed by Dunnett’s multiple comparison test versus NT *P < 0.05.

To gain insight into the upstream signaling events responsible for the transcriptional differences, we further explored the initial observation that kenpaullone activates ERK but does not engage Akt, in contrast to VEGF-C suggesting its effects may be mediated through an alternative kinase-dependent pathway. We investigated other known kinases reported to be targeted by kenpaullone beyond GSK3β. We compiled a list of known direct targets together with the reported percentage of residual kinase activity remaining at 0.5 µM and 10 µM (35, 36), and selected a panel of small-molecule inhibitors for each kinase to evaluate their individual contribution to LEC sprouting (*SI Appendix*, Table S3). A dose response of each compound was tested on LEC spheroids using the same conditions as the original screen (Fig. 5*A* and *SI Appendix*, Fig. S7). Among them, only the MAP4K4 inhibitor, DMX-5804, induced a strong sprouting response, suggesting that MAP4K4 might be a key mediator of its activity. In contrast, inhibition of the other target kinases had no significant effect (*SI Appendix*, Fig. S7*A*), indicating these were unlikely to be responsible for kenpaullone’s lymphangiogenic activity. To validate the involvement of MAP4K4 in the prosprouting effect, we tested additional MAP4K4 inhibitors with similar, low-mid nanomolar range potencies (MAP4K4-IN-3, PF-062306933, and GNE-495 with their reported MAP4K4 inhibitory potency, alongside DMX-5804 and kenpaullone–*SI Appendix*, Table S4) (37–40). Consistently, they all displayed sigmoidal dose–response curves and similar EC_50_ values, further supporting the involvement of MAP4K4 in regulating LEC sprouting (*SI Appendix*, Fig. S7*B*). To confirm that kenpaullone directly inhibits MAP4K4 kinase activity, we performed an in vitro kinase enzymatic radiometric assay. The result demonstrated that kenpaullone significantly reduced MAP4K4 enzymatic activity in a dose-dependent manner with an IC_50_ of 66 nM, consistent with previous studies (37) and only 4% MAP4K4 residual activity remaining at 10 µM kenpaullone (*SI Appendix*, Fig. S7*C*). In contrast, the analogue KP-AN-A exhibited reduced potency, with an IC_50_ of 378 nM, and 20% MAP4K4 activity persisted even at the highest concentrations tested (*SI Appendix*, Fig. S7*D*). To further support MAP4K4 inhibition as a mechanism of action, we revisited our bulk RNA sequencing dataset. We filtered the KEGG “MAPK signaling pathway” gene set with DEGs in response to VEGF-C and kenpaullone and generated a heatmap (*SI Appendix*, Fig. S8*A*). This analysis revealed altered expression of multiple MAPK-associated genes, including transcriptional regulators such as ATF2, JUN, and MYC, which were validated by qPCR (*SI Appendix*, Fig. S8*B*). This suggests that kenpaullone-mediated suppression of MAP4K4 activity alters the MAPK signaling cascade, potentially contributing to the observed sprouting phenotype. To validate the role of MAP4K4 in regulating lymphatic sprouting, we performed siRNA-mediated knockdown in LECs. After transfection, both mRNA and protein levels were decreased by 75% and 80%, respectively, confirming the efficiency of gene silencing (Fig. 5 *B* and *C*). Consistent with our pharmacological findings, MAP4K4 knockdown significantly enhanced sprouting in LEC spheroids compared to cells transfected with control siRNA (siCT) (Fig. 5*D*). In addition, Western Blot analysis revealed that MAP4K4 silencing led to a marked increase in phosphorylated ERK1/2 (p-P42/44) levels (Fig. 5*E*), consistent with our earlier observation that kenpaullone stimulates ERK phosphorylation. Since ERK signaling is a well-established driver of lymphangiogenesis, these findings suggest that kenpaullone at least in part, acts by inhibiting MAP4K4 activity, thereby relieving suppression of ERK pathway activity, to promote LEC spheroid sprouting.

We next sought to evaluate whether kenpaullone directly targeted MAP4K4 in LECs. To this end, we generated chemical probes to investigate kenpaullone’s subcellular localization (azide probes). The addition of an azide group to kenpaullone or KP-AN-A, a less potent analogue (KP-AZ and KP-AN-A-AZ, respectively, *SI Appendix*, Fig. S9*A*) did not alter their respective potency or efficacy to induce sprouting (*SI Appendix*, Fig. S9*B*). A strain-promoted azide-alkyne cycloaddition (SPAAC) bioorthoganol conjugation reaction was then performed on the treated LEC monolayer to attach BP Fluor 647 DBCO to the azide probes. Cells treated with KP-AZ exhibited stronger fluorescence signals compared to KP-treated cells, as expected. Interestingly, KP-AZ expression appeared enriched within the endoplasmic reticulum (ER), as indicated by coimmunostaining with the ER marker Calnexin (*SI Appendix*, Fig. S9*C*). Importantly, MAP4K4 also showed predominant localization within the ER (*SI Appendix*, Fig. S9*C*), suggesting that kenpaullone and MAP4K4 shared a similar subcellular distribution.

To test whether kenpaullone directly interacts with MAP4K4 we next synthesized photo affinity labeling (PAL) probes (41) from kenpaullone and the negative control compound KP-AN-A by addition of a diazirine photoaffinity group and an alkyne click handle (KP-DA and KP-AN-A-DA, respectively; *SI Appendix*, Fig. S10*A*). Similarly to the addition of the azide group, these modifications did not alter potency or efficacy to induce spheroid sprouting (*SI Appendix*, Fig. S10*B*). In addition, we tested if these probes also activated the ERK pathway. In LEC monolayers, ERK phosphorylation was significantly increased by both kenpaullone and KP-DA compared to control, whereas this effect was blunted in the presence of the negative probes, KP-AN-A and KP-AN-A-DA (*SI Appendix*, Fig. S10 *C* and *D*). Consistent with these findings, pharmacological MAP4K4 inhibitors (DMX5804 and PF06260933) also increased ERK phosphorylation. Similar results were observed in treated spheroids, confirming that ERK activation is essential for sprouting (*SI Appendix*, Fig. S10 *E* and *F*). Upon UV irradiation, the diazirine group produces a highly reactive carbene species that covalently binds to nearby interacting proteins. Following this, biotin-azide was attached to the probe via a copper (I)-catalyzed azide-alkyne cycloaddition (CuAAC) click reaction. Biotin was detected only in those samples treated with PAL probes, as expected (*SI Appendix*, Fig. S10*G*). Notably, the same MAP4K4 band was also detected with Biotin antibody, indicating that MAP4K4 had been covalently trapped by the PAL probes. Furthermore, the competition assay (KP-DA+KP) led to a partial reduction in the biotin signal, while the negative PAL probes (KP-AN-A-DA) produced a markedly weaker biotin signal (*SI Appendix*, Fig. S10*H*). Similar results were obtained for GSK3-β but with a weaker biotin signal at the corresponding band, suggesting that kenpaullone preferentially interacts with MAP4K4 in LEC. All together, these results demonstrate that Kenpaullone binds directly to MAP4K4, with higher affinity than GSK3β, in LECs to induce lymphangiogenesis.

**Fig. 6.**
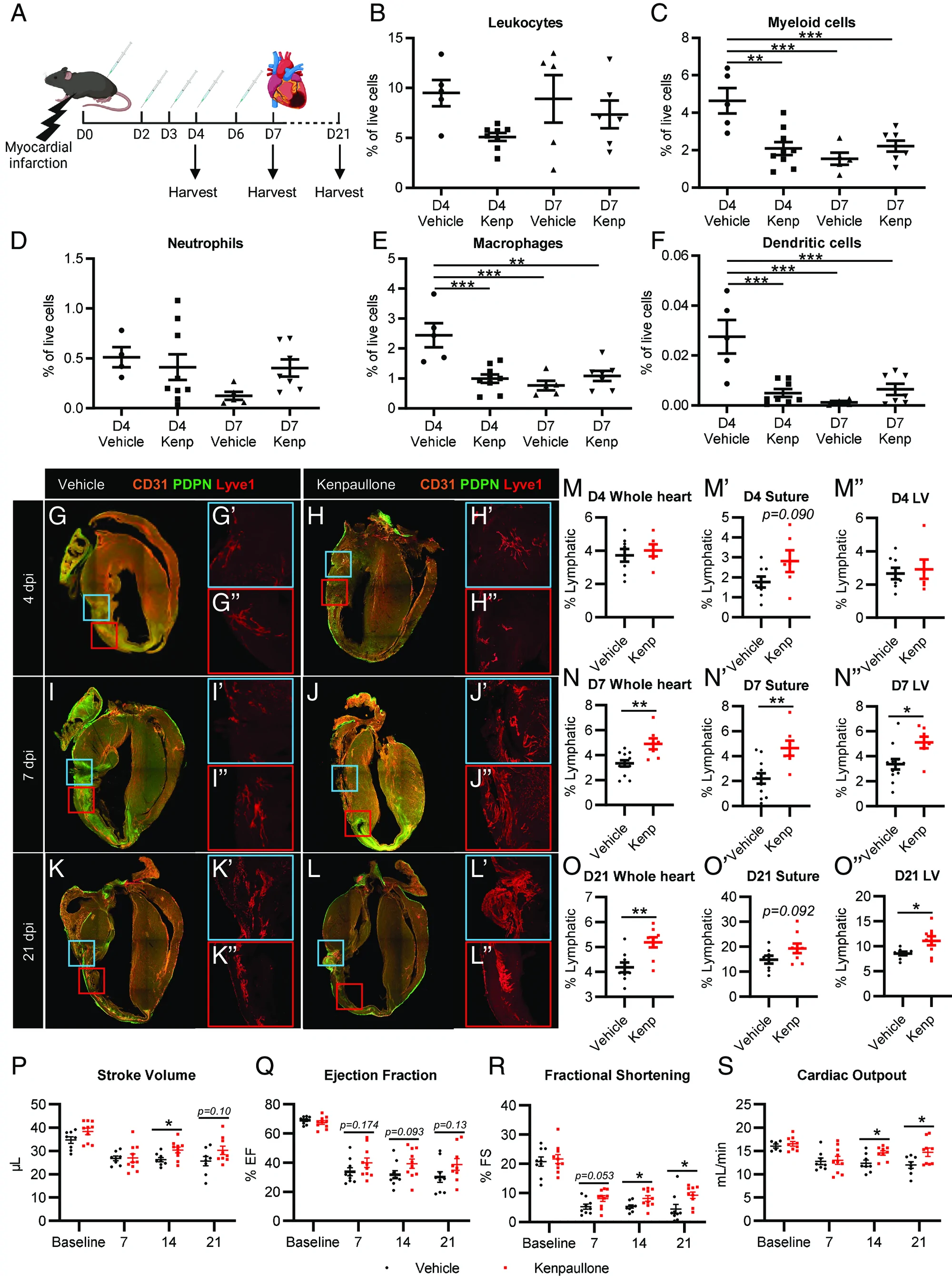
Kenpaullone treatment promotes in vivo lymphangiogenesis and improves cardiac function after myocardial infarction. (*A*) Schematic representation of treatment timeline with harvesting time points for further analysis. (*B*–*F*) Characterization of cardiac immune cell content in vehicle- and Kenpaullone–treated mice. Heart were collected on day 4 and 7 after MI and analyzed by flow cytometry using antibodies against CD45 [pan-leukocyte marker (*B*)], CD11b [CD45+ CD11b+, myeloid cells (*C*)], Ly6G [CD45+CD11b+Ly6G+, neutrophils (*D*)], F4/80 [CD45+CD11b+Ly6G - F4/80+, macrophages (*E*)], and CD11c [CD45+CD11b+Ly6G - F4/80 - CD11c+, dendritic cells (*F*)]. 7AAD staining was used to exclude dead cells. Data are presented as mean ± SEM; vehicle, n = 5 hearts; Kenpaullone, n = 7 to 9 hearts. One-way ANOVA followed by Tukey’s multiple comparisons test (**P < 0.01; ***P < 0.001). (*G*–*L*) Representative vibratome sections (200 µm) of infarcted hearts 4, 7, and 21 d post injury respectively stained for CD31 (orange), PDPN (green), and Lyve1 (red). (Scale bar: 900 µm.) Higher magnification showing suture area (blue box–*G’–L’*) and infarct area in the left ventricle (red box–*G”–L”*). (Scale bar: 300 µm.) (*M–O*) Quantification of the % of lymphatic area in different regions of interest (whole heart, suture and LV: Left Ventricle) using the Lyve1 staining. Data are presented as mean ± SEM; n = 6 to 12, *t* test *P < 0.05, **P < 0.01. (*P*–*S*) Echocardiography measurement of cardiac function at baseline, 7 dpi, 14 dpi, and 21 dpi including stroke volume (*P*), ejection fraction (*Q*), fractional shortening (*R*), and cardiac output (*S*). Data are presented as mean ± SEM; vehicle, n = 8; Kenpaullone, n = 10. Multiple t tests for each time point *P < 0.05.

Kenpaullone promotes lymphatic sprouting in vitro, but its relevance in vivo remains unknown. To investigate its potential therapeutic effects in the context of heart injury, we used a mouse model of MI induced by permanent ligation of the left anterior descending coronary artery. Kenpaullone or vehicle was administered systemically by i.p. injection following MI at days 0, 2, 3, 4, and 6 post injury (dpi) and hearts were harvested for further analysis at different time points (Fig. 6*A*).

To assess the local immune response, we performed flow cytometry on digested heart tissue harvested at 4 and 7 d postinjury (dpi), focusing on major immune cell populations. At 4 dpi, kenpaullone-treated mice showed a reduction of CD45^+^ leukocytes (vehicle, 9.49% ± 1.32; kenpaullone, 5.09% ± 0.41; *P* = 0.12), statistically significant within the myeloid cell compartment (vehicle, 4.63% ± 0.68; kenpaullone, 2.09% ± 0.34; *P* = 0.0012). More specifically, the number of CD45^+^CD11b^+^Ly6G^−^F4/80^+^ macrophages (vehicle, 2.44% ± 0.41; kenpaullone, 0.99% ± 0.14; *P* = 0.0005) and CD45^+^CD11b^+^Ly6G^−^F4/80^–^CD11c^+^ dendritic cells (vehicle, 0.028% ± 0.007; kenpaullone, 0.005% ± 0.0016; *P* = 0.0004) were also significantly and notably decreased following kenpaullone treatment. In contrast, CD45^+^CD11b^+^Ly6G^+^ neutrophil levels remained similar between both groups (vehicle, 0.51% ± 0.10; kenpaullone, 0.41% ± 0.13; *P* = 0.9330) (Fig. 6 *B*–*F*). By 7 dpi, these differences were no longer observed across all immune cell populations analyzed, including leukocytes (vehicle, 8.92% ± 2.39; kenpaullone, 7.35% ± 1.39; *P* = 0.8593), myeloid cells (vehicle, 1.55% ± 0.32; kenpaullone, 2.22% ± 0.3; *P* = 0.6877), macrophages (vehicle, 0.76% ± 0.16; kenpaullone, 1.09% ± 0.67; *P* = 0.7395), dendritic cells (vehicle, 0.0013% ± 0.0006; kenpaullone, 0.006% ± 0.001; *P* = 0.7236), and neutrophils (vehicle, 0.13% ± 0.04; Kenpaullone, 0.40% ± 0.19; *P* = 0.3503). These data suggest that kenpaullone selectively modulates the early inflammatory response post-MI without affecting later immune cell dynamics during resolution.

To investigate the spatial and temporal dynamics of lymphatic remodeling following kenpaullone treatment, we performed imaging on 200 µm vibratome sections of hearts at 4, 7, and 21 dpi enabling visualization of intact lymphatic vessels. Tissues were stained with Podoplanin (a lymphatic marker but also expressed in epicardium and fibroblast), CD31 (an endothelial marker), and Lyve1 to specifically detect lymphatic vessels. Quantification was performed across the whole heart and specifically within the suture area and infarcted left ventricle (LV). Lymphatic expansion was assessed by measuring the percentage area covered by Lyve1^+^ vessels in each region. At 4 dpi, compare to vehicle-treated mice, Kenpaullone triggered an increase of lymphatic vessels area only around the suture area (vehicle, 1.76% ± 0.28; kenpaullone, 2.81% ± 0.54; *P* = 0.090), where the injury was induced (Fig. 6 *G”*, *H”*, and *M”*). At 7 dpi, lymphangiogenesis was significantly increased in the Kenpaullone group across the entire heart (vehicle, 3.33% ± 0.26; kenpaullone, 4.90% ± 0.44; *P* = 0.0044) (Fig. 6 *I*, *J*, and *N*) and specifically in the suture area (vehicle, 2.20% ± 0.41; kenpaullone, 4.64% ± 0.60; *P* = 0.0029) (Fig.6 *I”*, *J”* and *N”*) and infarcted LV (vehicle, 3.36% ± 0.41; kenpaullone, 5.10% ± 0.47; *P* = 0.016) (Fig. 6 *I”*, *J”*, and *N”*). This difference persisted at 21 dpi, with kenpaullone-treated mice showing greater coverage of lymphatics in the whole heart (vehicle, 4.18% ± 0.19; kenpaullone, 5.19% ± 0.20; *P* = 0.0023) (Fig. 6 *K*, *L*, and *O*) and infarcted LV (vehicle, 8.55% ± 0.37; kenpaullone, 11.09% ± 0.91; *P* = 0.026) (Fig. 6 *K”*, *L”*, and *O”*). Notably, and as reported in other studies (5, 42), lymphangiogenesis is endogenously stimulated following MI and here, we showed that kenpaullone further enhanced and sustained this response. Collectively, these results confirmed than kenpaullone promotes lymphangiogenesis following MI. To determine whether this response was accompanied by a broader vascular expansion, we quantified the total CD31-positive vascular area across the whole heart. No significant differences were observed between vehicle and kenpaullone-treated hearts at any time point (*SI Appendix*, Fig. S11), suggesting that kenpaullone does not induce a global increase in vascular density following MI.

To assess cardiac function following kenpaullone treatment, we performed echocardiography at baseline, 7, 14, and 21 dpi. Kenpaullone treatment resulted in a significant increase in stroke volume at 14 dpi (vehicle, 26.28 µL ± 0.98; kenpaullone, 30.38 µL ± 1.23; *P* = 0.021) and the values remained higher than vehicle at 21 dpi (vehicle, 25.62 µL ± 1.98; kenpaullone, 30.23 µL ± 1.81; *P* = 0.10), although not statistically significant at the later timepoint (Fig. 6*P*). The ejection fraction (EF) increased in kenpaullone-treated mice compared to controls albeit this was not significant (7 dpi: vehicle, 33.76% ± 2.77; kenpaullone, 39.90% ± 3.26; *P* = 0.17; 14 dpi: vehicle, 31.70% ± 2.80; kenpaullone, 39.29% ± 3.17; *P* = 0.092; 21 dpi: vehicle, 30.15% ± 3.64; kenpaullone, 38.77% ± 4.00; *P* = 0.13) (Fig. 6*Q*). However, fractional shortening (FS) was significantly increased at 14 and 21 dpi (14 dpi: vehicle, 5.31% ± 0.56, kenpaullone, 8.10% ± 0.94, *P* = 0.024; 21 dpi: vehicle, 4.46% ± 1.60, kenpaullone, 9.20% ± 1.11, *P* = 0.027), with a near-significant trend already visible at 7 dpi (vehicle, 5.29% ± 0.88; kenpaullone, 8.05% ± 0.98; *P* = 0.053) (Fig. 6*R*) suggesting the ventricle was contracting more efficiently in the kenpaullone-treated mice. This was supported by significantly increased cardiac output (CO) at 14 and 21 dpi (14 dpi: vehicle, 12.36 mL/min ± 0.69, kenpaullone, 14.57 mL/min ± 0.46, *P* = 0.016; 21 dpi: vehicle, 11.98 mL/min ± 0.68, kenpaullone, 14.72 mL/min ± 0.90, *P* = 0.032) (Fig. 6*S*). Taken together, these data suggest that kenpaullone treatment contributes to significantly improved cardiac performance during post-MI recovery.

## Discussion

The beneficial role of therapeutic lymphangiogenesis following MI has previously been established, however there is an unmet need to identify therapeutic regulators of this process. Our study provides a robust phenotypic discovery pipeline to identify such regulators, whereby combining high-content 3D screening, chemical probe development, target deconvolution, and in vivo validation, to identify Kenpaullone, a canonical GSK3b inhibitor, as a significant small molecule inducer of lymphatic remodeling and improved cardiac outcome post-MI.

Our high-content 3D-LEC sprouting assay revealed that kenpaullone promotes lymphatic sprouting independently of VEGF-C. Mechanistically, this effect was mediated through MAP4K4 inhibition, resulting in ERK pathway activation and downstream up-regulation of *Vegfd* as a known inducer of lymphangiogenesis. While previous studies have implicated MAP4K4 in lymphatic endothelial biology and ERK regulation, the independent identification of this pathway through our unbiased phenotypic screen validates the robustness and biological relevance of our screening assay. Furthermore, kenpaullone exerted beneficial effects in vivo in an MI model, where it reduced cardiac immune cell infiltration as early as 4 dpi, enhanced lymphatic vessel expansion from 4 dpi to 21 dpi and was associated with significantly improved cardiac function. Together with previous studies linking enhanced lymphangiogenesis post-MI (4–7), these findings support the concept that kenpaullone-mediated lymphangiogenesis contributes to enhanced cardiac recovery following myocardial infarction. To further assess the vascular specificity of kenpaullone, we evaluated its effects on blood endothelial cells both in vitro and in vivo. Unlike LECs, HUVEC spheroids did not exhibit enhanced sprouting following kenpaullone treatment, and total CD31-positive vascular area remained unchanged after MI. These findings suggest that kenpaullone preferentially promotes lymphangiogenesis rather than inducing a global vascular expansion. Nevertheless, further dedicated analyses of blood vessel density and function will be required to fully determine whether kenpaullone also influences the blood vasculature.

This study successfully employs a spheroid-based assay as a phenotypic screen to identify inducers of lymphangiogenesis. Previous screening efforts have included a LEC-coated beads assay to investigate lymphatic sprouting inhibitors, and, using the Library of Pharmacologically Active Compounds 1280 (LOPAC®^1280^), identified statins and MEK1/2 as suppressors of lymphangiogenesis (43). The fact that MEK1/2 inhibition emerged as an antilymphangiogenic signal in their study underscores the critical importance of ERK1/2 signaling in lymphatic sprouting. The identification of kenpaullone in our assay aligns with its repeated emergence as a hit across diverse repurposing drug screening efforts, notably in cancer, neuroscience, and regenerative medicine (26, 44, 45). Kenpaullone exerts multiple effects across a number of biological systems, including enhancing the effect of temozolomide against glioblastoma (27), inducing neuroprotection via KCC2 upregulation, reducing pathologic pain-like behavior in a mouse model of nerve injury (25), proproliferative and proregenerative potential on airway basal cells in 3D organoid assays (46) and modulation of osteoclastogenesis (47). More relevant to cardiovascular indications, kenpaullone induces miR-182, suppressing cardiomyocyte hypoxia-induced apoptosis in vitro and synthesized derivatives prevented cardiac fibrosis to improve heart function in a rat ischemia/reperfusion injury model (48). In addition, kenpaullone can protect cardiomyocytes from oxidative stress (49). Thus, given the broad biological activity of kenpaullone, the improved cardiac outcome observed following MI could reflect the combined modulation of multiple cell types and reparative pathways. Although our data clearly establish kenpaullone as a potent inducer of lymphangiogenesis, future studies using cell type–specific approaches will be required to define the relative contribution of lymphatic remodeling to the overall therapeutic benefit. Our work therefore extends this pleiotropic activity of kenpaullone to include lymphatic endothelial biology, identifying lymphangiogenesis as a component of its therapeutic activity both in vitro and in vivo.

Mechanistically, our data highlight MAP4K4 as the key mediator of kenpaullone’s prolymphangiogenic activity in vitro in contrast to its more canonical role as a GSK3β-inhibitor. GSK3β inhibition has previously been reported to promote lymphangiogenesis in vitro, specifically in lung lymphatic endothelial cells, in a β-catenin-dependent pathway (50). While these findings are interesting and highlight that GSK3β inhibitors can impact LEC behavior, it is important to note that the assays employed were based on a 2D Matrigel-based tubulogenesis assay which do not model the complex 3D cellular behaviors involved in lymphangiogenesis. In contrast, our spheroid assay provides a more physiologically relevant assay to assess vessel sprouting. In our hands, neither Wnt recombinant proteins, Wnt activators nor GSK3β inhibition (genetically or via allosteric inhibitors) were able to induce sprouting, suggesting that kenpaullone’s action was not driven by the GSK3β-Wnt pathway. Instead by synthesizing KP-AZ and KP-DA probes, we validated MAP4K4 as a direct kenpaullone target and also provided insight into the spatial context of this interaction. KP-AZ predominantly localized to the ER, colocalizing with MAP4K4, suggesting that kenpaullone engages MAP4K4 in this subcellular compartment. Although KP-AZ retained biological activity comparable to unmodified kenpaullone, the introduction of the azide group and subsequent click chemistry could potentially influence its intracellular distribution. Therefore, the observed ER localization should be interpreted with appropriate caution and confirmed using complementary approaches. Importantly, photoaffinity labeling with KP-DA independently demonstrated specific covalent binding to MAP4K4 and preserved ERK activation, whereas negative control probes neither activated ERK nor captured MAP4K4 in LEC. These data are consistent with other studies in which kenpaullone’s protective effects on motor neurones involved MAP4K4, rather than GSK3β (26, 44, 45).

MAP4K4 has recently emerged as an important regulator of lymphatic biology during development. Endothelial specific MAP4K4 knockout in mice results in dilated lymphatic capillaries, valve defects, and impaired lymphatic flow, highlighting a role in establishing and maintaining lymphatic architecture during development (51). Here, we showed that in the adult injured heart, kenpaullone promoted lymphangiogenesis and improved tissue repair. Mechanistically, our in vitro data suggest that MAP4K4 inhibition in LECs is responsible for this increased lymphangiogenesis. These observations are not contradictory but rather reflect distinct biological requirements: MAP4K4 is necessary to preserve lymphatic vessel integrity and function during development, whereas in the adult setting of injury, its inhibition can relieve brakes on sprouting and remodeling. Importantly, MAP4K4 inhibition has resulted in therapeutic effects in many cardiovascular contexts; including eliciting improved survival and function of human iPSC-derived cardiomyocytes and reduced infarct size in cardiac ischemia–reperfusion injury in mice (37). MAP4K4 knockdown and pharmacological inhibition through DMX-5804 ameliorate cardiac microvascular endothelial dysfunction in diabetic cardiomyopathy (52). MAP4K4 has also been described as a regulator of vascular inflammation and endothelial activation involved in the development of atherosclerotic plaques (53). Together with our findings, these studies position MAP4K4 as a promising therapeutic target at the intersection of cardiomyocyte survival, vascular repair, immune modulation, and lymphatic remodeling.

Interestingly, in our in vitro model, MAP4K4 inhibition resulted in the activation of the ERK1/2 signaling pathway, a central driver of lymphangiogenesis. This could appear counterintuitive given that MAP4K4 has mostly been described as an upstream activator of ERK1/2, as seen, for instance, in hepatocytes (54), oncogenic studies (55), and inflammation-related settings (56, 57). However, MAP4K4-ERK1/2 crosstalk appears to be highly context-dependent. Indeed, MAP4K4 knockdown in endothelial cells, including LECs, led to elevated basal levels of phospho-ERK (51), thus supporting our findings. Together, these results indicate that, at least in the lymphatic endothelium, MAP4K4 functions as a negative regulator of ERK1/2 activity, and its inhibition to promote ERK-signaling enables lymphatic sprouting and remodeling. Finally, MAP4K4 is a key regulator of cytoskeletal and mechanical dynamics, controlling focal-adhesion, adherent junction stability, actomyosin contractility, the tension balance at cell–cell adhesion and controls forces transmitted among cells (58). It has also been identified as a mechano-associated gene (59). In this context, MAP4K4 modulation within LEC spheroids may shift the balance of tension across the spheroid surface, locally altering traction forces, and thereby facilitate sprouting initiation. Whether these mechanical changes directly drive the prosprouting effect remains to be experimentally determined.

In conclusion, our work identified kenpaullone as a novel lymphangiogenic modulator and highlights MAP4K4 inhibition as a new potential pathway driving lymphatic endothelial sprouting. A significant strength of our approach is the use of a 3D-LEC spheroid assay, which captures the complexity of sprouting behavior and is scalable for phenotypic drug screening. By combining this platform with in vivo validation in a MI mouse model, we established a framework for discovering new modulators of lymphangiogenesis. The clinical potential of therapeutic lymphangiogenesis is further highlighted by ongoing translational efforts using lymphangiogenic growth factors, including VEGF-D gene therapy for patients with refractory angina (ClinicalTrials.gov identifier: NCT03039751) (60). Although this approach differs mechanistically from small-molecule therapy, it underscores the growing interest in targeting the cardiac lymphatic vasculature to improve cardiovascular outcomes. These findings also provide preclinical evidence that targeting MAP4K4 may represent a promising therapeutic strategy to enhance lymphatic remodeling to optimize cardiac repair.

## Materials and Methods

### Chemistry Methods.

Chemistry methods are available within *SI Appendix*.

### Cell Culture and 3D-LEC Sprouting Assay.

Human Primary Lymphatic Endothelial Cells (LEC) (Lonza CC-2543) were cultured in EGM-2 MV medium following the manufacturer’s instructions and used for between passages 6 to 9. LEC spheroids were generated overnight in EGM-I, a growth factor-depleted formulation of EGM-2, using AggreWell™ plates and embedded in type I collagen (1 mg/mL). Spheroids were treated with compounds and incubated for 24 h before 4% PFA fixation and then stained with Phalloidin and DAPI. Detailed culture conditions, spheroids generation, collagen preparation, compound treatment, and staining procedures are provided in the *SI Appendix*.

### Phenotypic Screening and Imaging.

The spheroid assay was miniaturized to a 384-well format using automated pipetting and screened against Tocris Stem Cell, Epigenetics, and Kinase Inhibitor libraries, together with a custom-made compound library. Positive and negative controls were included on each plate to assess assay performance and calculate Z’-factors. Following treatment, spheroids were imaged using an ImageXpress Pico automated imaging system, and sprouting was quantified using an automated CellProfiler-based analysis pipeline. Concentration–response experiments were performed using eight-point serial dilutions, which each condition tested in duplicate. Detailed screening and image-analysis procedures are provided in the *SI Appendix*.

### Protein Extraction and Immunoblot.

Proteins were extracted from LEC spheroids or monolayer using RIPA buffer supplemented with protease and phosphatase inhibitors, quantified by BCA assay and analyzed by SDS-PAGE and immunoblotting. For signaling time course-experiments, LECs were serum-starved overnight in EGM-2 MV medium lacking supplements before stimulation with VEGF-C or kenpaullone for the indicated times. Detailed protein extraction, immunoblotting, and antibody procedures are provided in the *SI Appendix*.

### RNA Extraction, RT, and qPCR.

RNA was extracted from LEC spheroids using the Quick-RNA Miniprep Kit or from monolayer cultures using TRizol™ and was then reverse transcribed with SuperScript™ III enzyme according to the manufacturers’ instructions. Gene expression was quantified by RT-qPCR using SYBR Green and determined by the 2^−ΔΔCT^ method. Data were normalized to 36B4 housekeeping gene expression. Primer sequences are listed in *SI Appendix*, Table S5. Detailed RNA extraction, reverse transcription, and qPCR procedures are provided in the *SI Appendix*.

### Bulk RNA Sequencing.

Bulk RNA sequencing was performed by BGI. For each sample, ≥500 ng of total RNA was submitted for library preparation. RNA integrity and quality control were assessed by the service provider prior library construction, and all samples passed quality thresholds. Sequencing libraries were generated according to standard protocols and sequenced on the DNBseq™ platform (BGI) using paired-end 100 bp reads (PE100). Approximately 20 million read pairs were generated per sample, corresponding to ~4.0 GB of clean data per sample. Raw sequencing reads were processed and analyzed using the Tom online analysis platform provided by BGI. Three independent biological replicates were included per experimental condition.

### siRNA Transfection.

LECs were transfected with a final concentration of 10 nM siRNA targeting GSK3β, MAP4K4, or a Silencer™ Negative Control using Lipofectamine RNAiMAX according to the manufacturer’s instructions. 48 h posttransfection, cells were used to generate spheroids, which were then embedded in type I collagen and assessed for sprouting as described above. Detailed transfection and spheroid-generation procedures are provided in the *SI Appendix*.

### Photoaffinity Labeling.

Confluent LEC were treated with kenpaullone photoaffinity probes, followed by UV irradiation, cell lysis, and click chemistry to conjugate biotin for subsequent detection of probe-labeled proteins by immunoblotting. Detailed probe treatment, photo-crosslinking, click chemistry, and protein-processing procedures are provided in the *SI Appendix*.

### Animal Models and MI.

Mice were housed and maintained in a controlled environment by University of Oxford Biomedical Services. 8 to 12 wk wild-type C57BL/6J animals, from both sexes, were used for all experiments (Charles River Laboratories). All animal experiments were carried out in accordance with UK home office project license PP3194787 and were compliant with the UK Animals (Scientific Procedures) Act 1986.

Myocardial Infarction was induced in isoflurane-anesthetized mice by permanent ligation of the left anterior descending coronary artery as previously described (5). Animals were administered either 30 mg/kg body weight of Kenpaullone (Tocris) or vehicle (PBS-5% DMSO) by *i.p.* injections on days 0, 2, 3, 4, and 6 after surgery. Hearts were harvested at 4-, 7-, or 21 d postinjury (dpi).

### Flow Cytometry.

Post-MI hearts were enzymatically dissociated to generate single-cell suspension followed by red blood cell lysis and staining with antibodies against CD45, CD11b, Ly6G, F4/80, and CD11c to identify leukocyte, myeloid, neutrophil, macrophage, and dendritic cell populations, respectively. 7-aminoactinomycin D high fluorescence dye (7AAD) was added to determine cell viability and exclude dead cells. The Flow cytometric analyses were performed using a BD FACSAriaIII and FlowJo software. Antibodies and detailed tissue-dissociation procedures are provided in *SI Appendix*, Table S6.

### Vibratome.

4-, 7-, and 21-dpi hearts were longitudinally sectioned at 200 µm using a vibratome. Sections were immunostained for lymphatic and vascular markers and imaged on a Zeiss 980 Confocal microscope with an Airyscan detector. Lymphatic vessel density was quantified as the percentage of area covered by Lyve1^+^ across three distinct regions of interest (ROIs): the whole heart (excluding the atria), the left ventricle, and the suture area. Global vascular density was measured in the same way using CD31*^+^* staining with the ImageJ/FIJI software. Detailed sectioning, staining, imaging, and image-analysis procedures are provided in the *SI Appendix*.

### Echocardiography.

Cardiac function was assessed by transthoracic echocardiography using the Vevo F2 imaging system (FUJIFILM VisualSonics) at baseline and at 7, 14, and 21 dpi. Mice were anesthetized with isoflurane and maintained on a heated platform during imaging. Two-dimensional B-mode images were acquired in both long-axis and short-axis views, and cardiac function parameters, including ejection fraction, fractional shortening, stroke volume, and cardiac output, were calculated using Vevo Lab analysis software according to manufacturer guidelines. Detailed acquisition is provided in the *SI Appendix*.

## Supplementary Material

Appendix 01 (PDF)

## Data Availability

The bulk RNA-seq data generated in this study is deposited in the NCBI Gene Expression Omnibus (GEO) and are accessible under accession number GSE348365 (61). All other data are included in the article and/or *SI Appendix*.
