# Supplementary material for: Phenotypic screening identifies kenpaullone as a prolymphangiogenic compound to improve heart repair following myocardial infarction
Source: Proc Natl Acad Sci U S A. 2026 Oct 6;123(41):e2619427123. doi: 10.1073/pnas.2619427123 (PMC13663767; doi:10.1073/pnas.2619427123)
Supplement: Supplementary file 1 [file pnas.2619427123.sapp.pdf]

PNAS

# Supporting Information for

Phenotypic screening identifies kenpaullone as a pro-lymphangiogenic compound to improve heart repair following myocardial infarction

Christophe Ravaud[1,2#], Caroline A. Cuoco[3,4#], Sarah Sigal[1,2], Hannah M. Unsworth[3,4], Susanna T.E. Cooper[1,2], Adam B. Lokman[1,2], Carole J.R. Bataille[3,4], Angela J. Russell[3,4], Paul R. Riley[1,2*]

[1]Institute of Developmental and Regenerative Medicine, University of Oxford, Oxford, OX3 7TY, UK;
[2]Department of Physiology, Anatomy and Genetics, University of Oxford, Oxford OX1 3PT, UK;
[3]Department of Pharmacology, Mansfield Road, University of Oxford, OX1 3QT, UK;
[4]Department of Chemistry, Chemistry Research Laboratory, University of Oxford, Mansfield Road, Oxford OX1 3TA, UK

[#]these individuals contributed equally

* Paul R. Riley, Institute of Developmental and Regenerative Medicine, University of Oxford, Oxford, OX3 7TY, UK, United Kingdom. Phone: +44 (0)1865 282366;

**Email:** paul.riley@idrm.ox.ac.uk

**This PDF file includes:**

Figures S1 to S11
Tables S1 to S6
Detailed Materials and Methods
Chemistry Methods

## Figures

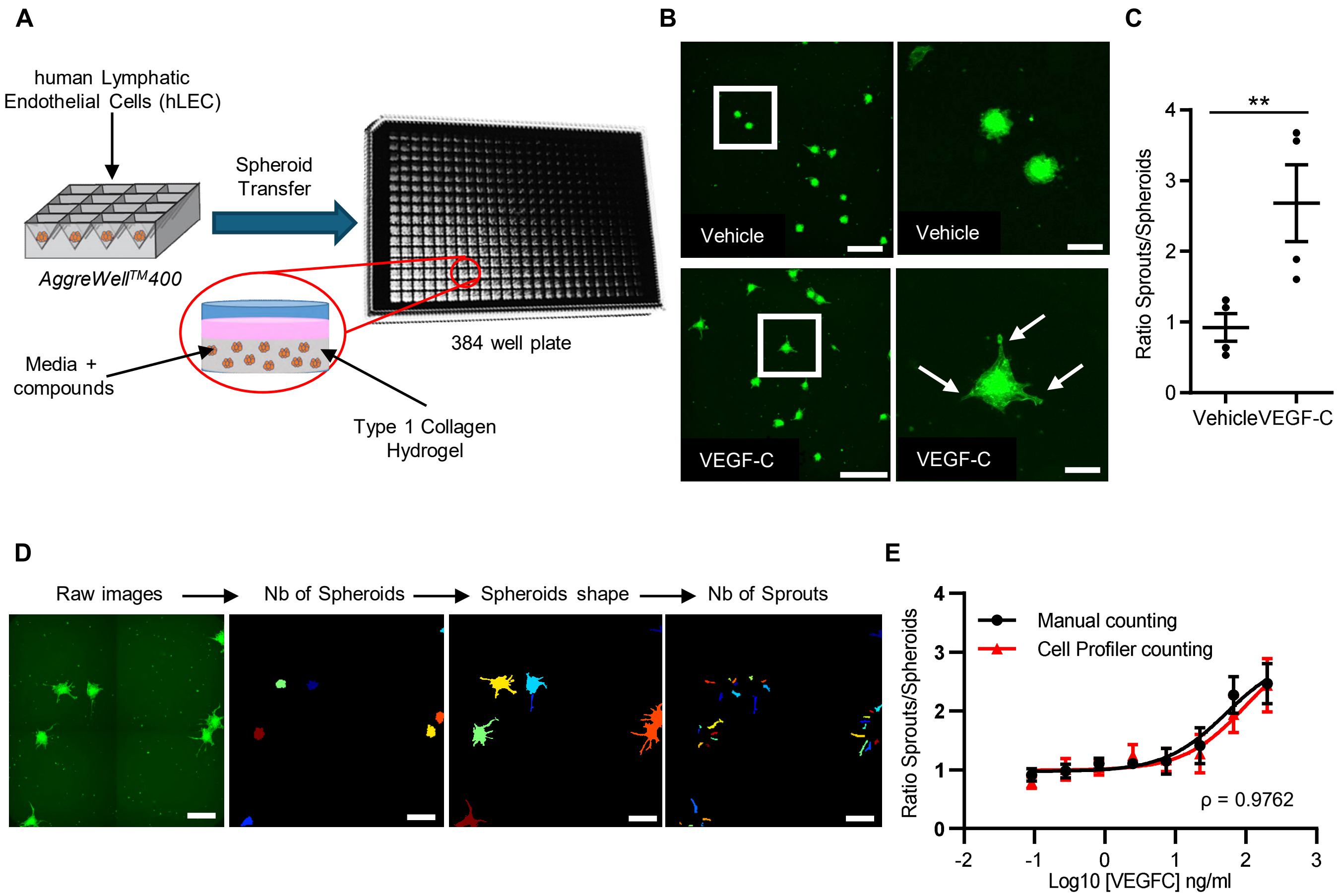


**Fig. S1. Workflow and validation of the 3D LEC spheroid sprouting assay.** (A) Schematic overview of the 3D LEC sprouting assay setup. (B) Representative image of vehicle- and VEGF-C (50 ng/mL)- treated spheroids stained with Phalloidin. Left panel represents the field of view of one well and right panel is a magnified view of the corresponding image. White arrows mark sprouts. Scale bars: 500µm and 125 µm respectively. (C) Quantification of the number of sprout/spheroids under vehicle and VEGF-C conditions. Each data point represents the ratio in 4 independent experiments. n=4; Error bars: S.E.M.; **p<0.01. (D) Workflow for automated spheroids and sprout detection using Cell Profiler. Scale bar: 250 µm. (E) Correlation between manual and automated quantification of mean sprout ratio per condition (each point represent the mean of 5 independent experiments; n = 8 paired conditions; Spearman r = 0.9762, p = 0.004)

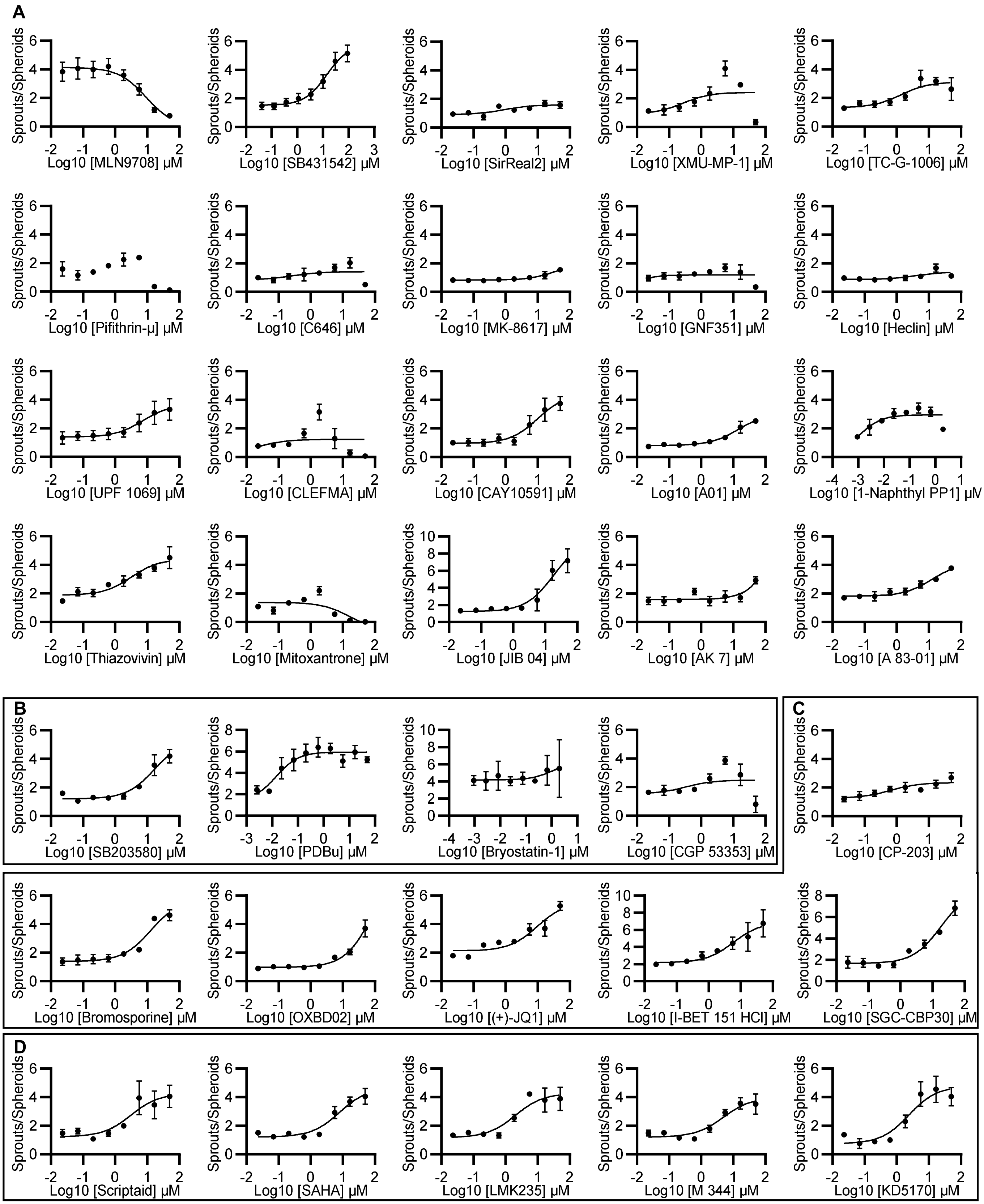
A
Sprouts/Spheroids
Log10 [MLN9708] μM
Log10 [SB431542] μM
Log10 [SirReal2] μM
Log10 [XMU-MP-1] μM
Log10 [TC-G-1006] μM
Log10 [Pifithrin-μ] μM
Log10 [C646] μM
Log10 [MK-8617] μM
Log10 [GNF351] μM
Log10 [Heclin] μM
Log10 [UPF 1069] μM
Log10 [CLEFMA] μM
Log10 [CAY10591] μM
Log10 [A01] μM
Log10 [1-Naphthyl PP1] μM
Log10 [Thiazovivin] μM
Log10 [Mitoxantrone] μM
Log10 [JIB 04] μM
Log10 [AK 7] μM
Log10 [A 83-01] μM
B
Log10 [SB203580] μM
Log10 [PDBu] μM
Log10 [Bryostatin-1] μM
Log10 [CGP 53353] μM
C
Log10 [CP-203] μM
Log10 [Bromosporine] μM
Log10 [OXBD02] μM
Log10 [(+)-JQ1] μM
Log10 [I-BET 151 HCl] μM
Log10 [SGC-CBP30] μM
D
Log10 [Scriptaid] μM
Log10 [SAHA] μM
Log10 [LMK235] μM
Log10 [M 344] μM
Log10 [KD5170] μM

**Fig. S2. Dose-response characterization of additional screening hits.** (A) Compounds with varied targets. (B) PKC modulators. (C) Bromodomain inhibitors. (D) HDAC inhibitors. Data are represented as mean ± SEM, n = 2 independent experiments

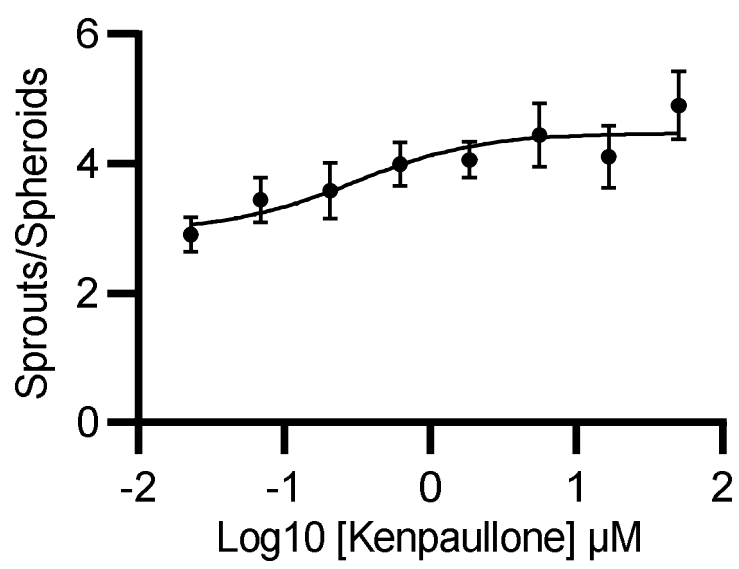


**Fig. S3. Kenpaullone induces limited sprouting response in HUVEC spheroids.** Dose-response curves of Kenpaullone on HUVEC spheroids. Data are shown as mean ± SEM, n=4 independent experiments

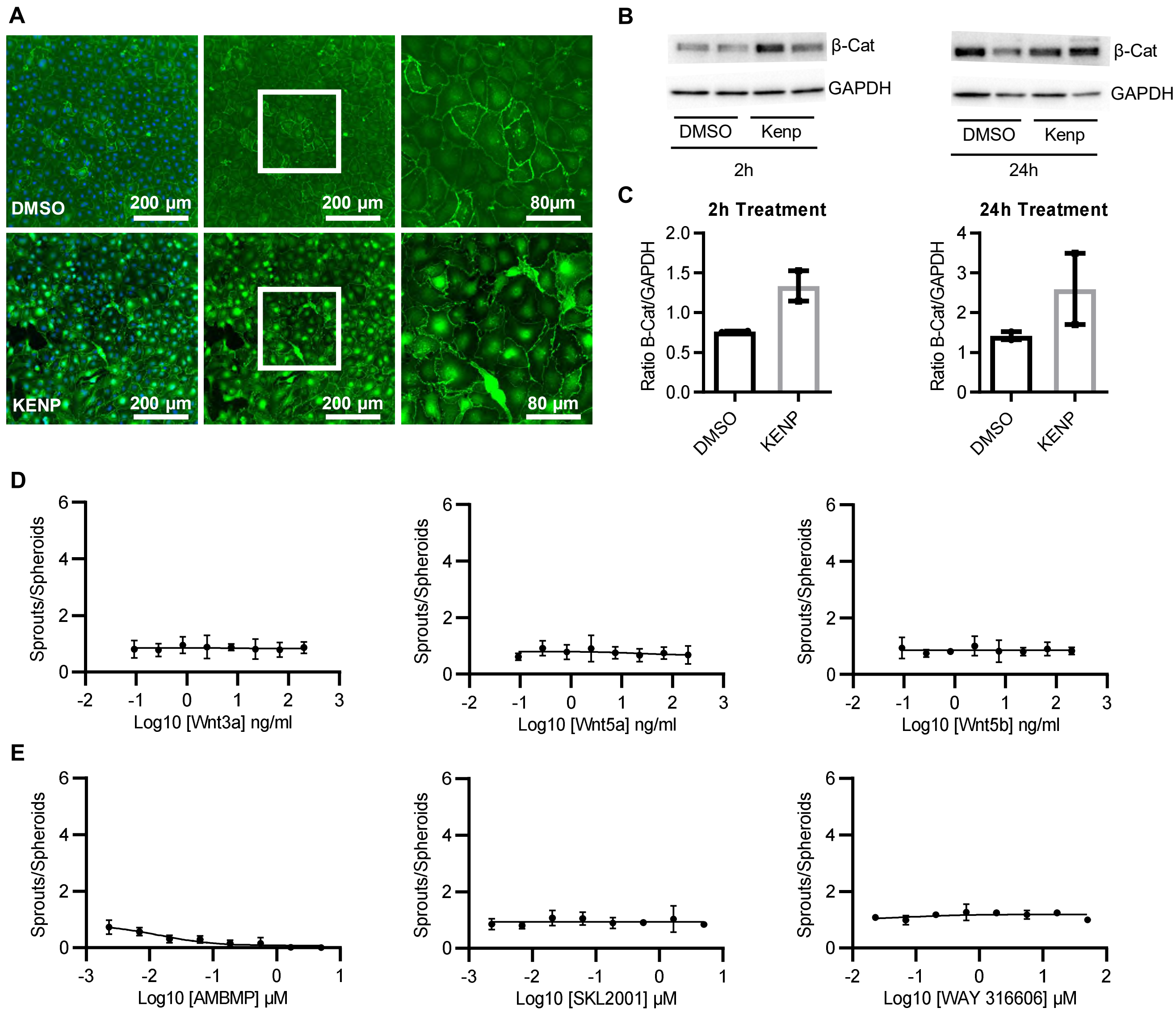


**Fig. S4. Wnt pathway activation is not responsible for Kenpaullone-induced LEC sprouting** (A) Left panel: Co staining β-catenin (green) and DAPI (cell nuclei – blue) of LECs treated with vehicle or Kenpaullone (10 µM) for 24h. Central panel is β-catenin only and right panel a magnified view of the corresponding image. (B) Representative Western Blot of total β-catenin levels at 2h and 24h following Kenpaullone treatment. (C) Quantification analysis of β-catenin expression normalised to GAPDH. Data are shown as mean ± SEM, n = 2 independent experiments. (D-E) Dose response curves of various human recombinant Wnt proteins (D) and pharmacological Wnt agonists (E) on LEC spheroid sprouting. Data are shown as mean ± SEM, n = 3

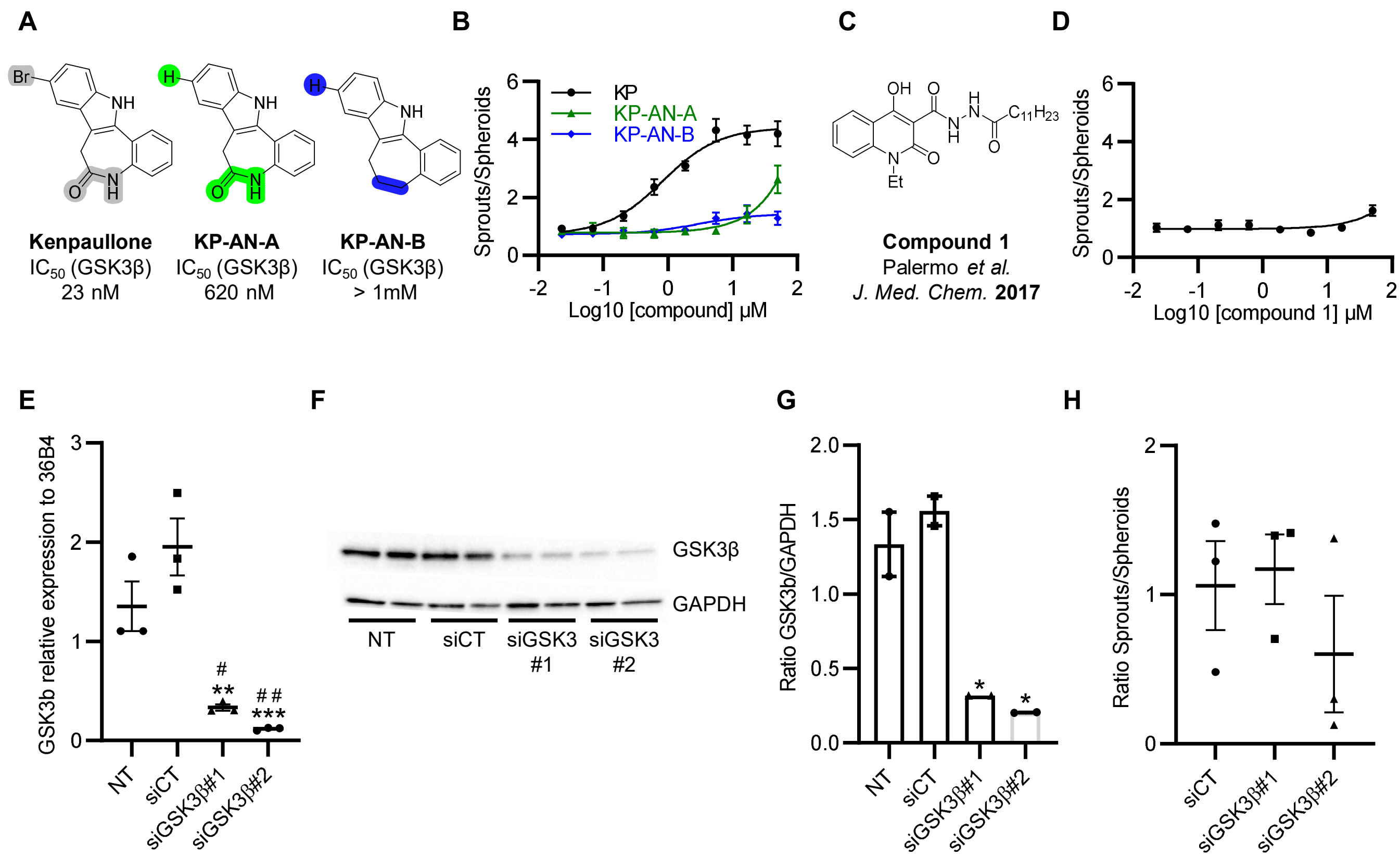


**Fig. S5. GSK3β inhibition alone is not responsible for Kenpaullone-induced LEC sprouting** (A) Structures of parent compound kenpaullone and analogues KP-AN-A and KP-AN-B with their respective IC50's for GSK3β kinase binding activity. (B) Dose response curves of Kenpaullone and synthesized analogues on LEC spheroid sprouting. (C) GSK3β allosteric inhibitor chemical structure. (D) Dose response curves of compound 1 on LEC spheroid sprouting. Data are shown as mean ± SEM, n=6. (E) qPCR analysis of LEC transfected with siRNA targeting GSK3β. One way ANOVA followed by multiple comparison test (n=3; $^{\#}p < 0.05$, $^{\#\#}p < 0.01$ to NT; $^{**}p < 0.01$, $^{***}p < 0.001$ to siCT) (G) Representative western blot of GSK3β levels 2 days post transfection. (G) Quantification of GSK3β expression normalised to GAPDH. Data are shown as mean ± SEM, n=2. One-way ANOVA followed by a Dunnett multiple comparison test versus NT $^{*}p < 0.05$. (H) Sprouting assay on transfected LEC. Data are shown as mean ± SEM, n=3. NT: Non-Transfected; siCT: scramble siRNA

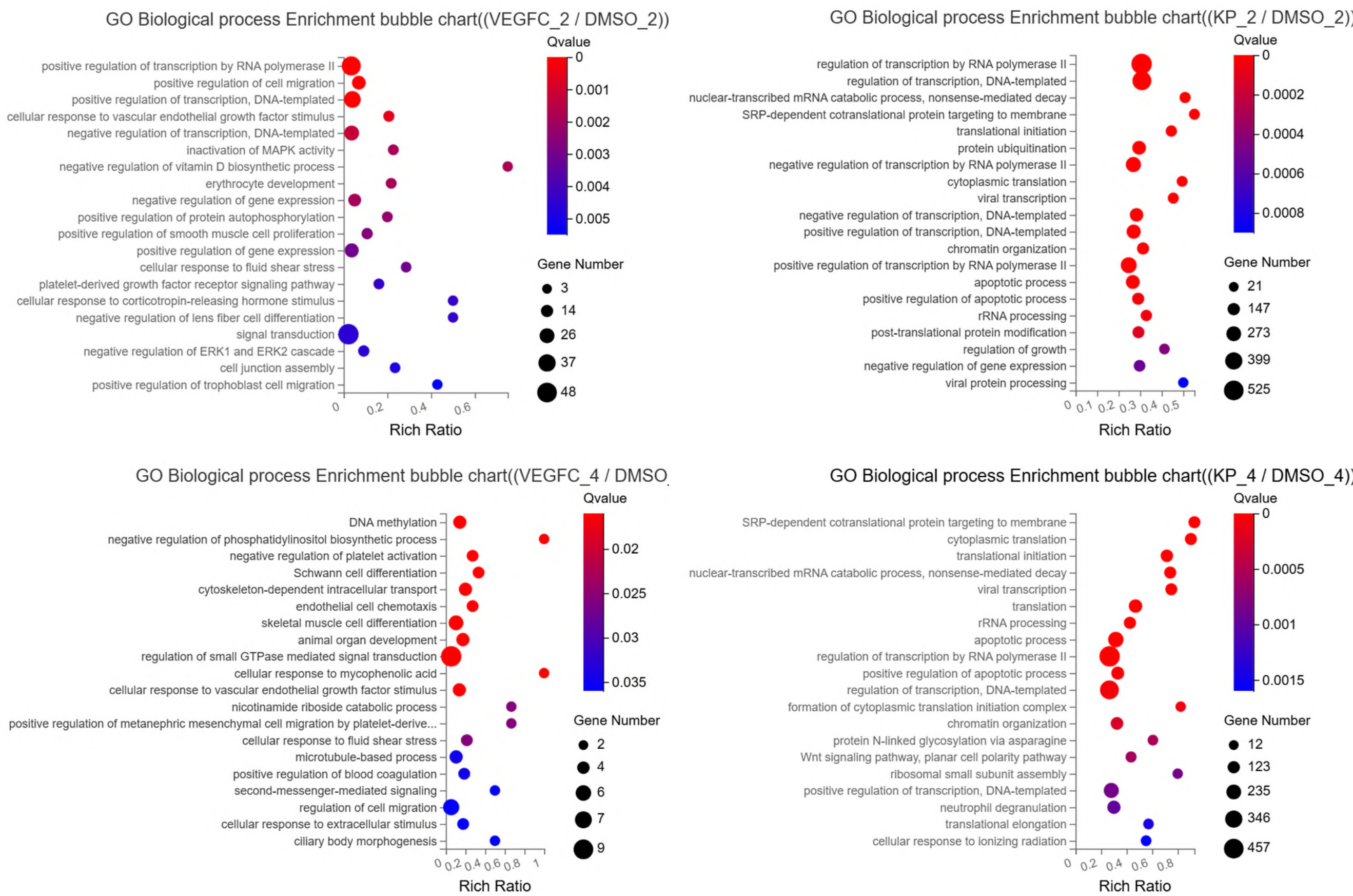


**Fig. S6. Gene Ontology (GO) analysis of differentially expressed genes from bulk RNA-seq of spheroids treated with VEGF-C or kenpaullone for 2 or 4 hours**

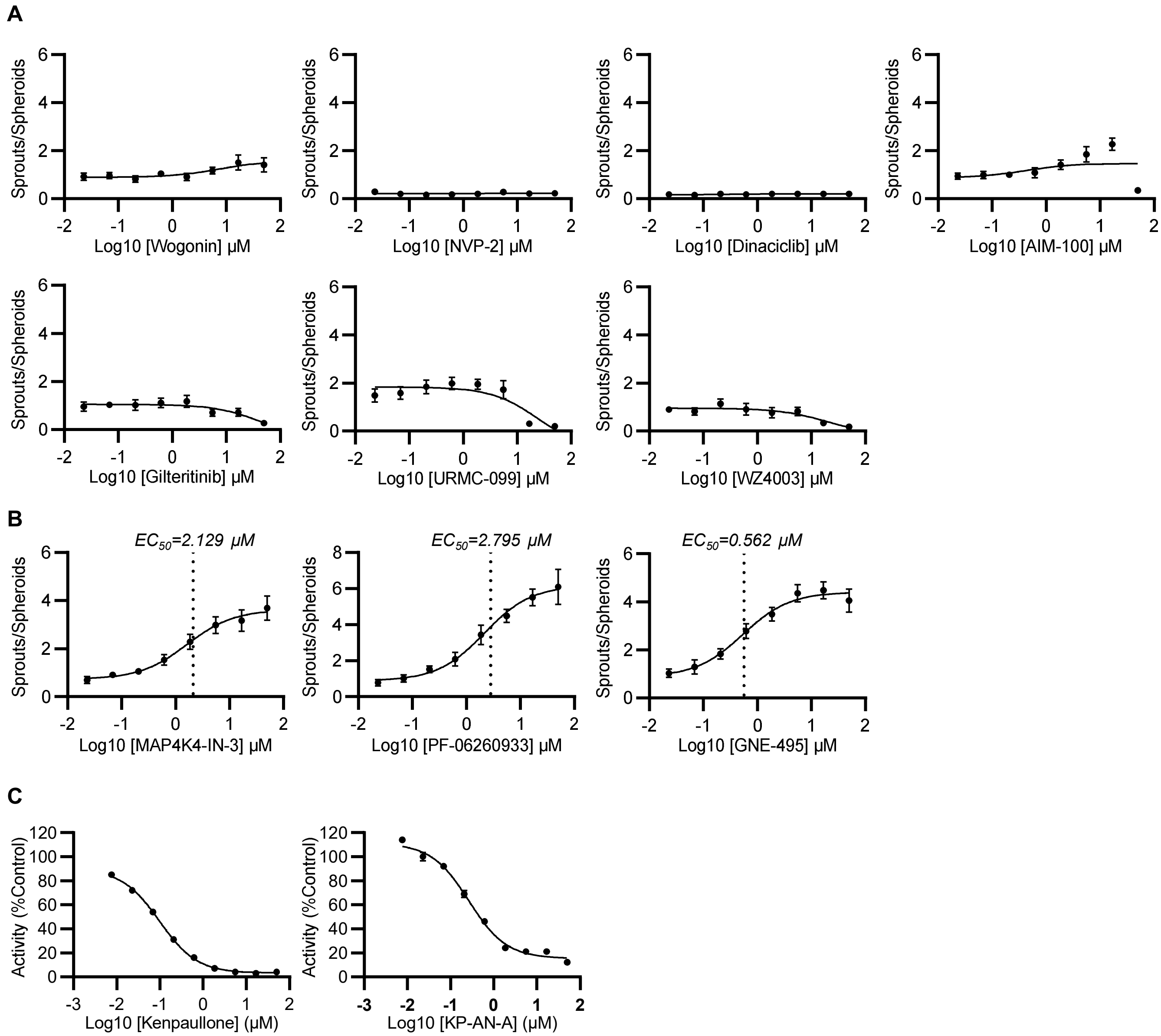


**Fig. S7. Effect of small molecule inhibitors of Kenpaullone's targeted kinases on LEC spouting** (A) Dose response analysis of representative small molecule inhibitors targeting kinases reported to be inhibited by Kenpaullone. Data are shown as mean ± SEM, n=3 independent experiments. (B) Dose response analysis of various MAP4K4 small molecule inhibitors with their respective EC50. Data are shown as mean ± SEM, n=4 independent experiments. (C-D) In vitro kinase radiometric assay measuring kenpaullone (C) and KP-AN-A (D) potency to inhibit MAP4K4 activity, with their respective IC50. Data shown as mean ± SEM, n=2.

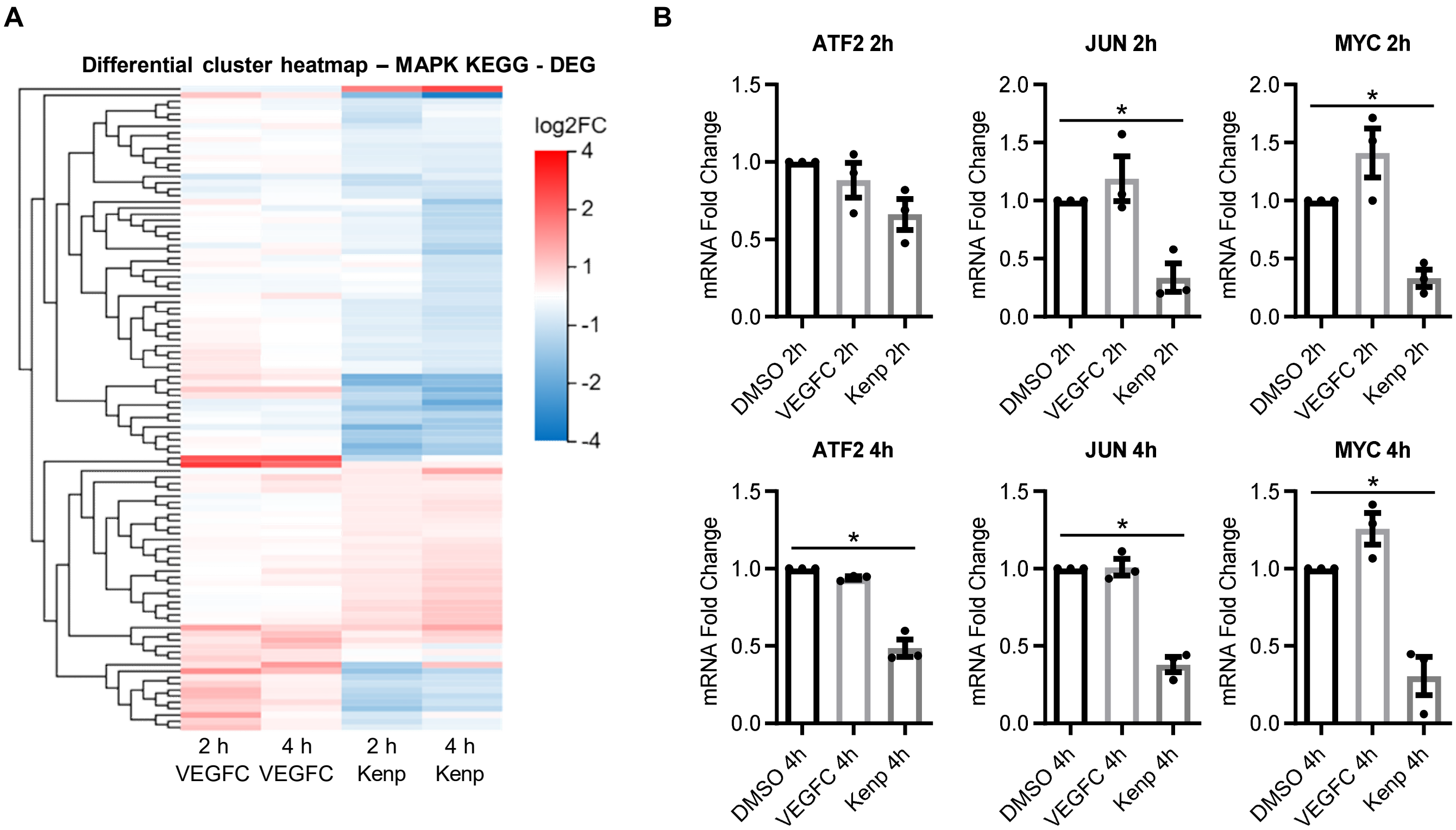


**Fig. S8. Kenpaullone modulates the MAPK pathway and MAP4K4-related genes expression**(A) Heatmap of DEGs associated with the KEGG MAP signalling pathway in LEC spheroids treated with VEGFC or Kenpaullone compared to vehicle. Log2 fold change is shown, with red indicating upregulation and blue indicating downregulation. (B) qPCR validation of selected MAP4K4-related genes identified as differentially expression in the RNA-seq analysis. Data are shown as mean ± SEM, n=3, one way ANOVA followed by a Dunnett's multiple comparison test versus DMSO *p<0.05.

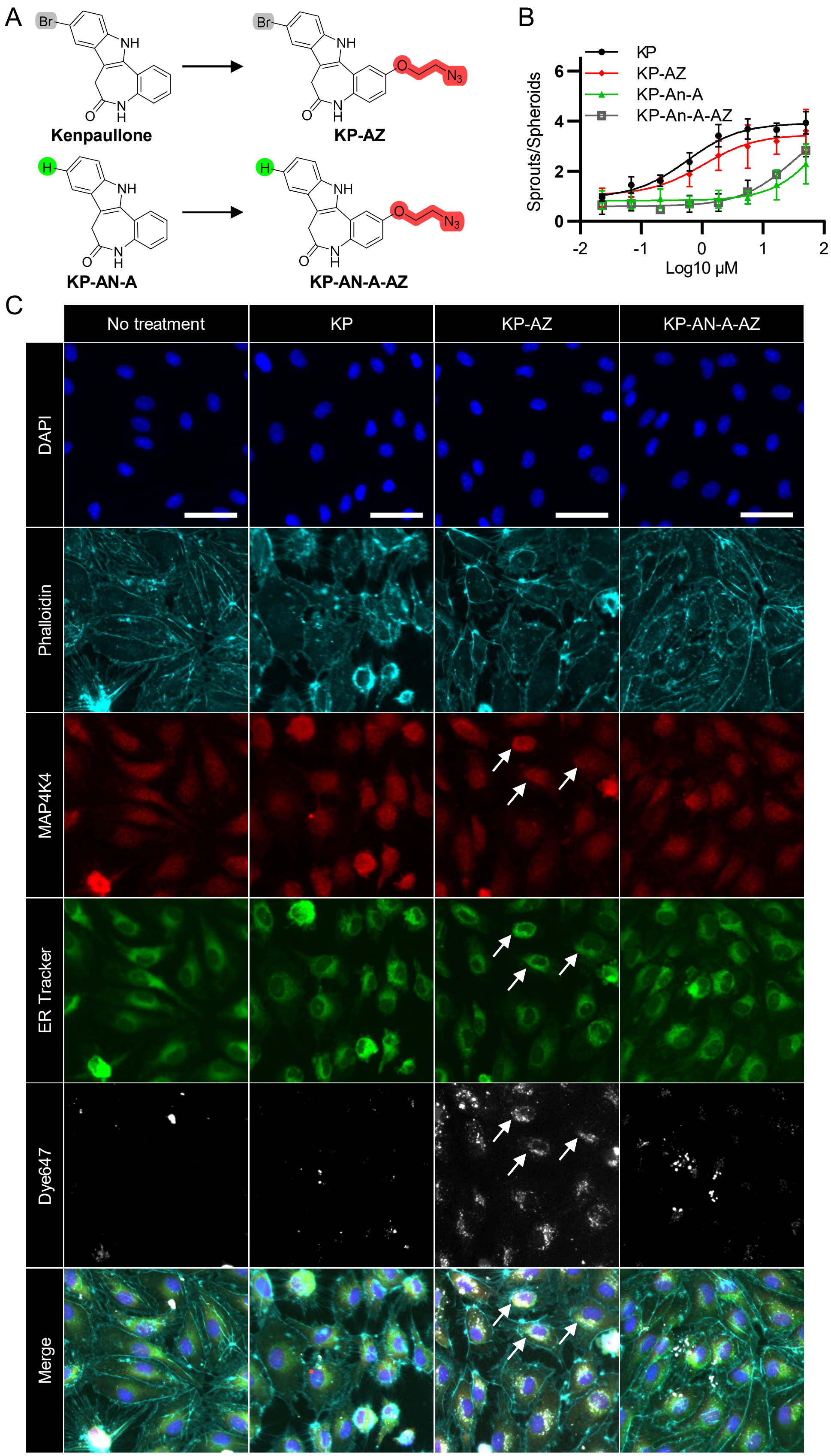
A
Br
NH
O
H
N
Kenpaullone
KP-AZ
N3
H
KP-AN-A
KP-AN-A-AZ
B
KP
KP-AZ
KP-An-A
KP-An-A-AZ
Sprouts/Spheroids
Log10 μM
C
No treatment
KP
KP-AZ
KP-AN-A-AZ
DAPI
Phalloidin
MAP4K4
ER Tracker
Dye647
Merge

**Fig. S9. Kenpaullone co-localises with MAP4K4** (A) Structures of Kenpaullone (KP) and Kenpaullone Analog A (KP-AN-A), along with their corresponding azide-functionalized derivatives (KP-AZ and KP-AN-A-AZ). Azide groups were introduced to enable subcellular localization studies in live cells using strain-promoted azide–alkyne click chemistry (SPAAC). (B) Dose response curves of Kenpaullone and synthesized analogues on LEC spheroid sprouting. Data are represented as mean ± SEM, n = 4-7 independent experiments (C) Immunofluorescence of LECs treated for 2 h with 10 μM of the indicated analogues. Azide-functionalized probes were detected by strain-promoted azide–alkyne click chemistry (SPAAC) using 10 μM BP Fluor 647 DBCO. The endoplasmic reticulum (ER) was labelled with ER-Tracker Green, and MAP4K4 was detected by immunostaining. White arrows indicate regions of colocalization between MAP4K4, ER, and KP-AZ. Scale bar = 50 μm

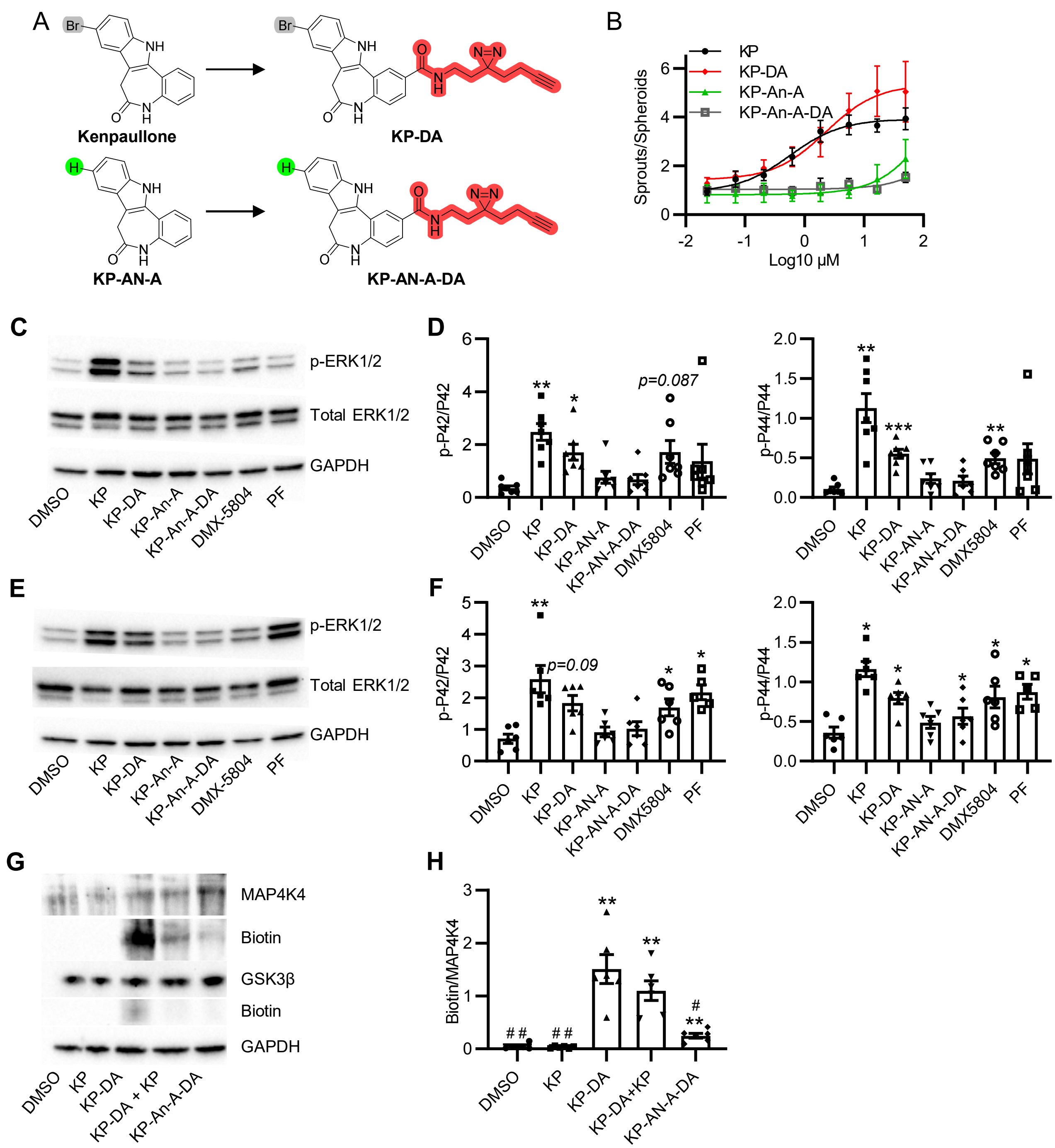


**Fig. S10. Kenpaullone directly targets MAP4K4** (A) Structures of Kenpaullone (KP) and Kenpaullone Analog A (KP-AN-A) modified with a diazirine group (KP-DA and KP-AN-A-DA) to enable photoaffinity labelling. (B) Dose response curves of Kenpaullone and synthesized analogues on LEC spheroid sprouting. Data are represented as mean ± SEM, n = 4-7 independent experiments (C) Representative Western Blot of p-ERK1/2 and ERK1/2 two hours post treatment of LEC monolayer with 10 µM of KP, its analogues or MAP4K4 inhibitors (DMX5804 and PF-06260933). GAPDH was used as a loading control. (D) Quantification analysis of p-ERK1/2 normalized to total ERK. Data are shown as mean ± SEM, n=6. One-way ANOVA followed by Dunnett's multiple comparison test versus the DMSO control *p < 0.05, **p<0.01, ***p<0.001. (E) Representative Western Blot of p-ERK1/2 and ERK1/2 two hours post treatment of LEC spheroids

with 10 μM of KP, its analogues or MAP4K4 inhibitors (DMX5804 and PF-06260933). GAPDH was used as a loading control. (F) Quantification analysis of p-ERK1/2 normalized to total ERK. Data are shown as mean ± SEM, n=6. One-way ANOVA followed by Dunnett's multiple comparison test versus the DMSO control *$p < 0.05$. (G) Representative Western Blot showing MAP4K4 and GSK3b, with biotin signals detected at the matching bands in pre-pull-down lysates from DMSO, KP, KP-DA, KP-DA + KP (competition with 50 μM KP) and KP-AN-A-DA treatments (10 μM). GAPDH was used as a loading control. (H) Quantification of MAP4K4 biotinylation shown as the MAP4K4/biotin ratio for each treatment condition. Data are shown as mean ± SEM, n=6. One-way ANOVA followed by Dunnett's multiple comparison test versus either the DMSO control **$p < 0.01$ or KP-DA #$p<0.05$, ##$p < 0.01$.

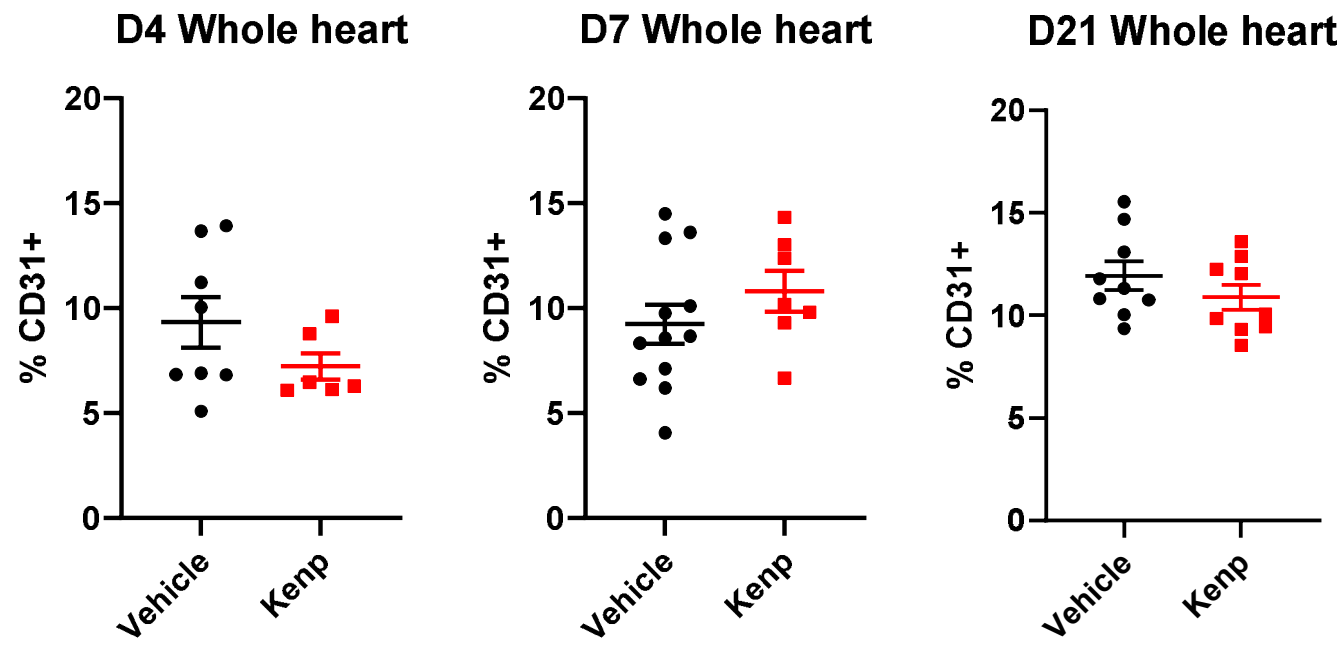


**Fig. S11. Kenpaullone does not induce a global increase in vascular density.** Quantification of the % of CD31⁺ vasculature in the whole heart at 4, 7 and 21 dpi. Data are presented as mean ± SEM; n=6-12.

## Tables

**Table S1. Compounds with no significant effect on LEC sprouting assay at 1 μM**

List of compounds tested at 1 μM that did not meet the predefined hit criteria (fold-change > 2.5 and $p < 0.05$). Data are presented as mean fold change from three independent experiments

| Compounds – 1 µM | Libraries | Fold Average | pValue |
|---|---|---|---|
| Wnt Agonist I | Custom Library | 0 | 0.158555544 |
| PD 173074 | Tocris StemCells Library | 0.048226704 | 0.141232949 |
| b-AP15 | Custom Library | 0.128783129 | 0.22007681 |
| Triptolide | Tocris Epigenetic Library | 0.344262408 | 0.325478945 |
| WIKI4 | Tocris StemCells Library | 0.378555349 | 0.300110384 |
| Ryuvidine | Tocris Kinase Library | 0.396437169 | 0.335211302 |
| UNC 0646 | Tocris Epigenetic Library | 0.428899013 | 0.333853429 |
| JK 184 | Tocris StemCells Library | 0.447209404 | 0.412534781 |
| PD 0325901 | Tocris StemCells Library | 0.448584887 | 0.391388119 |
| CI 994 | Tocris Epigenetic Library | 0.451863912 | 0.348459628 |
| Fluoxetine hydrochloride | Tocris StemCells Library | 0.453973037 | 0.36818582 |
| AP 24534 | Tocris StemCells Library | 0.456409827 | 0.371626779 |
| IQ 1 | Tocris StemCells Library | 0.503701378 | 0.429727966 |
| Ro 08-2750 | Tocris Kinase Library | 0.51649861 | 0.393030218 |
| SAHA | Tocris StemCells Library | 0.540652508 | 0.440144252 |
| PD 98059 | Tocris Kinase Library | 0.570205272 | 0.457152227 |
| API-2 | Tocris Kinase Library | 0.574966471 | 0.497787786 |
| IPA 3 | Tocris StemCells Library | 0.577791113 | 0.539361885 |
| WAY-262611 | Custom Library | 0.581684987 | 0.564563923 |
| Sodium 4-Phenylbutyrate | Tocris StemCells Library | 0.581971717 | 0.521480283 |
| IKK 16 | Tocris Kinase Library | 0.59335497 | 0.452796533 |
| NU 7026 | Tocris Kinase Library | 0.608316565 | 0.467908697 |
| GSK J5 | Tocris Epigenetic Library | 0.615875684 | 0.492254137 |
| LY 294002 hydrochloride | Tocris Kinase Library | 0.63658462 | 0.522079134 |
| Olomoucine | Tocris Kinase Library | 0.642330517 | 0.534669041 |
| SRT 1720 | Custom Library | 0.662632317 | 0.645994309 |
| TC-H 106 | Tocris Epigenetic Library | 0.66605241 | 0.56361615 |
| GSK 650394 | Tocris Kinase Library | 0.671389084 | 0.536725944 |
| KU 55933 | Tocris Epigenetic Library | 0.678690059 | 0.552946661 |
| KU-60019 | Custom Library | 0.679787577 | 0.663868009 |
| XAV 939 | Tocris StemCells Library | 0.686226225 | 0.561025717 |
| 1,2,3,4,5,6-Hexabromocyclohexane | Tocris Kinase Library | 0.690243262 | 0.582225259 |
| SL 327 | Tocris Kinase Library | 0.696782331 | 0.669988308 |
| PP 2 | Tocris Kinase Library | 0.718780349 | 0.656312642 |
| SP 600125 | Tocris StemCells Library | 0.719159611 | 0.562786583 |
| BMS 453 | Tocris StemCells Library | 0.724966884 | 0.589444889 |
| IDE 1 | Tocris StemCells Library | 0.72589947 | 0.605293321 |
| Lomeguatrib | Tocris Epigenetic Library | 0.72698244 | 0.596208503 |
| MC 1568 | Tocris Epigenetic Library | 0.733933273 | 0.594292682 |
| Sodium 4-Phenylbutyrate | Tocris Epigenetic Library | 0.737299243 | 0.637294222 |

| Compounds – 1 µM | Libraries | Fold Average | pValue |
|---|---|---|---|
| Ceramide | Tocris StemCells Library | 0.748320494 | 0.634481687 |
| SC79 | Custom Library | 0.761042491 | 0.751032882 |
| IBMX | Tocris StemCells Library | 0.773560922 | 0.695016764 |
| UNC 0224 | Tocris Epigenetic Library | 0.782634251 | 0.686861199 |
| FK-506 | Custom Library | 0.783209223 | 0.775441207 |
| FPA 124 | Tocris Kinase Library | 0.791081495 | 0.702599937 |
| FTY720, Fingolimod | Custom Library | 0.791895527 | 0.785069538 |
| LY2228820 | Custom Library | 0.791899609 | 0.78507398 |
| Sirtinol | Tocris Epigenetic Library | 0.796372482 | 0.680805914 |
| UNC 0638 | Tocris Epigenetic Library | 0.805357325 | 0.659053563 |
| DMH-1 | Tocris StemCells Library | 0.817805232 | 0.647933719 |
| CGP 57380 | Tocris Kinase Library | 0.821641021 | 0.699449263 |
| LY 294002 hydrochloride | Tocris StemCells Library | 0.824191136 | 0.741181206 |
| ZM 449829 | Tocris Kinase Library | 0.825632738 | 0.706098246 |
| Decitabine | Tocris Epigenetic Library | 0.83677731 | 0.772575902 |
| ER 27319 maleate | Tocris Kinase Library | 0.843562032 | 0.784872248 |
| ID 8 | Tocris StemCells Library | 0.843649045 | 0.795839058 |
| CP-466722 | Custom Library | 0.843906655 | 0.843370614 |
| TPCA-1 | Tocris Kinase Library | 0.84953443 | 0.824545526 |
| PS 48 | Tocris StemCells Library | 0.849601819 | 0.754920248 |
| nultin-3A | Custom Library | 0.852960258 | 0.853618596 |
| ZM 39923 hydrochloride | Tocris Kinase Library | 0.854141396 | 0.775674188 |
| SirReal2 | Custom Library | 0.854532111 | 0.855401177 |
| IG-4D-44 | Custom Library | 0.855980754 | 0.857043856 |
| SP 600125 | Tocris Kinase Library | 0.857320356 | 0.689575718 |
| SNS 314 mesylate | Tocris Epigenetic Library | 0.857864399 | 0.79706453 |
| SGI 1027 | Tocris Epigenetic Library | 0.858159178 | 0.76131199 |
| 4SC-101, Vidofludimus | Custom Library | 0.861547897 | 0.863362931 |
| 5-Azacytidine | Tocris StemCells Library | 0.864542848 | 0.80070436 |
| AM 580 | Tocris StemCells Library | 0.86538506 | 0.720114896 |
| Afuresertib | Custom Library | 0.870116548 | 0.873106223 |
| IG-4D-51 | Custom Library | 0.870469934 | 0.873508621 |
| ZM 447439 | Tocris Epigenetic Library | 0.875665212 | 0.856624533 |
| SB 415286 | Tocris Kinase Library | 0.88716089 | 0.761624205 |
| Forskolin | Tocris Epigenetic Library | 0.887328083 | 0.71931025 |
| A 366 | Tocris Epigenetic Library | 0.889507393 | 0.828934778 |
| UPF 1069 (IG-4D-49) | Custom Library | 0.896980533 | 0.903773542 |
| PCI 34051 | Tocris Epigenetic Library | 0.906147646 | 0.76345925 |
| Gallocyanine, IIIC3 | Custom Library | 0.908400759 | 0.916857417 |
| Retinoic acid | Tocris StemCells Library | 0.910030255 | 0.809346386 |
| TC-E 5003 | Tocris Epigenetic Library | 0.925314076 | 0.788260396 |

| Compounds – 1 µM | Libraries | Fold Average | pValue |
|---|---|---|---|
| IMD 0354 | Tocris Kinase Library | 0.931944132 | 0.896678837 |
| Prostaglandin E2 | Tocris StemCells Library | 0.940032992 | 0.799200421 |
| SC-10 | Custom Library | 0.941648661 | 0.955067578 |
| Tenovin -1 | Tocris Epigenetic Library | 0.947739203 | 0.937895519 |
| GW 583340 2HCl | Tocris Kinase Library | 0.948272335 | 0.896922013 |
| 7-Nitroindazole | Custom Library | 0.949074246 | 0.963618714 |
| UNC 926 hydrochloride | Tocris Epigenetic Library | 0.951112037 | 0.845421099 |
| PQ 401 | Tocris Kinase Library | 0.963389581 | 0.8901978 |
| CH 223191 | Tocris StemCells Library | 0.965411676 | 0.963268382 |
| Ro 31-8220 mesylate | Tocris Kinase Library | 0.969446054 | 0.829214277 |
| 20(S)-Hydroxycholesterol | Custom Library | 0.971627927 | 0.989613021 |
| Exo1 | Custom Library | 0.972658982 | 0.990801794 |
| ZM 323881 hydrochloride | Tocris Kinase Library | 0.973650348 | 0.863707019 |
| Bromosporine | Tocris Epigenetic Library | 0.97435302 | 0.933129085 |
| AZ1366 | Custom Library | 0.974924098 | 0.993413472 |
| Memantine (hydrochloride) | Custom Library | 0.976525395 | 0.995260141 |
| PI 828 | Tocris Kinase Library | 0.981206504 | 0.978380946 |
| NSC 693868 | Tocris Kinase Library | 0.982122843 | 0.869751935 |
| MLR 1023 | Custom Library | 0.989599724 | 0.989664331 |
| SB 239063 | Tocris Kinase Library | 0.991987179 | 0.862941892 |
| RG 108 | Tocris StemCells Library | 0.994214376 | 0.947965115 |
| DCP-LA, FR 236924 | Custom Library | 0.996143755 | 0.98211956 |
| PFI 1 | Tocris Epigenetic Library | 0.996280051 | 0.857509223 |
| Zebularine | Tocris Epigenetic Library | 0.997337314 | 0.882753722 |
| NH 125 | Tocris Kinase Library | 0.999463557 | 0.925760169 |
| TCS HDAC6 20b | Tocris Epigenetic Library | 1.001446364 | 0.954696517 |
| KU 55933 | Tocris Kinase Library | 1.003474252 | 0.984183268 |
| CGP 53353 | Tocris Kinase Library | 1.005224476 | 0.935084264 |
| BIBX 1382 dihydrochloride | Tocris Kinase Library | 1.008584014 | 0.960124818 |
| ZM 447439 | Tocris Kinase Library | 1.011919907 | 0.924254581 |
| PluriSIn 1 | Tocris StemCells Library | 1.014309546 | 0.921081538 |
| Kaempferol | Tocris Epigenetic Library | 1.018487516 | 0.941359722 |
| BIX 01294 | Tocris StemCells Library | 1.021473275 | 0.96967971 |
| NVP DPP 728 2HCl | Tocris StemCells Library | 1.022271349 | 0.969883927 |
| KU 60019 | Tocris Epigenetic Library | 1.025981345 | 0.975071665 |
| WHI-P 154 | Tocris StemCells Library | 1.027165644 | 0.964044157 |
| PD 198306 | Tocris Kinase Library | 1.037682626 | 0.911123352 |
| Y-27632 dihydrochloride | Tocris Kinase Library | 1.040487741 | 0.992070809 |
| UNC1999 | Custom Library | 1.042827242 | 0.928384439 |
| IOX 1 | Tocris Epigenetic Library | 1.043293668 | 0.928690721 |
| Cardiogenol C HCl | Tocris StemCells Library | 1.045472819 | 0.924539402 |

| Compounds – 1 µM | Libraries | Fold Average | pValue |
|---|---|---|---|
| 1-EBIO | Tocris StemCells Library | 1.046110783 | 0.972498074 |
| Lisinopril | Custom Library | 1.050836751 | 0.919191421 |
| Y-27632 dihydrochloride | Tocris StemCells Library | 1.053237426 | 0.906088523 |
| LY 303511 | Tocris Epigenetic Library | 1.053806755 | 0.900854694 |
| EC 23 | Tocris StemCells Library | 1.054022839 | 0.953349962 |
| PF 03814735 | Tocris Epigenetic Library | 1.056372932 | 0.963390871 |
| (±)-Bay K 8644 | Tocris StemCells Library | 1.057910361 | 0.910595335 |
| AG 490 | Tocris Kinase Library | 1.059156473 | 0.928568595 |
| PD 98059 | Tocris StemCells Library | 1.060321338 | 0.992479518 |
| m-3M3FBS | Custom Library | 1.060769445 | 0.907806033 |
| Retinoic acid | Tocris Epigenetic Library | 1.060844941 | 0.936224967 |
| Aminopurvalanol A | Tocris Kinase Library | 1.062102293 | 0.963064237 |
| PFI 3 | Tocris Epigenetic Library | 1.06331521 | 0.991294268 |
| SAG | Custom Library | 1.066934933 | 0.900748533 |
| Daminozide | Tocris Epigenetic Library | 1.069171263 | 0.984264998 |
| VR23 | Custom Library | 1.069873336 | 0.897387669 |
| L-Ascorbic acid | Tocris StemCells Library | 1.07124395 | 0.979892721 |
| SL 327 | Tocris Epigenetic Library | 1.082535528 | 0.979484126 |
| WP1130, Degrasyn | Custom Library | 1.082938162 | 0.882468808 |
| (-)-JQ1 | Custom Library | 1.082954775 | 0.882449422 |
| Troglitazone | Tocris StemCells Library | 1.086075982 | 0.949270323 |
| 6,2',4'-Trimethoxyflavone | Custom Library | 1.08639513 | 0.878527974 |
| LFM-A13 | Tocris Kinase Library | 1.094616987 | 0.871365218 |
| UNC0642 | Custom Library | 1.104833808 | 0.857562817 |
| GANT 61 | Tocris StemCells Library | 1.106446101 | 0.965881653 |
| OTSSP167 | Custom Library | 1.108227092 | 0.853714933 |
| C646 | Custom Library | 1.116003938 | 0.844910071 |
| Thioridazine hydrochloride | Tocris StemCells Library | 1.119021172 | 0.920959522 |
| Doxycycline hyclate | Tocris StemCells Library | 1.121998671 | 0.814868942 |
| PR-619 | Custom Library | 1.122737059 | 0.837303008 |
| Splitomicin | Tocris Epigenetic Library | 1.122879534 | 0.893761488 |
| U0126 | Tocris Epigenetic Library | 1.126960664 | 0.94131896 |
| AZD9496 | Custom Library | 1.133115526 | 0.825607634 |
| Iressa | Tocris Kinase Library | 1.136166285 | 0.881177212 |
| Barasertib-hQPA | Custom Library | 1.137859203 | 0.820275146 |
| PP 1 | Tocris Kinase Library | 1.143340377 | 0.892870361 |
| GSK2656157 | Custom Library | 1.148865259 | 0.807934857 |
| U0126 | Tocris Kinase Library | 1.149127383 | 0.762795356 |
| IG-4D-55 | Custom Library | 1.152152674 | 0.804258771 |
| Resveratrol | Tocris Epigenetic Library | 1.153051247 | 0.766429377 |
| EX 527, Selisistat | Custom Library | 1.15664718 | 0.799239373 |

| Compounds – 1 µM | Libraries | Fold Average | pValue |
|---|---|---|---|
| 3BDO | Custom Library | 1.158090464 | 0.797629067 |
| SBHA | Tocris Epigenetic Library | 1.1627208 | 0.823947415 |
| CGK 733 | Tocris Kinase Library | 1.170119378 | 0.795088212 |
| (-) MK 801 | Custom Library | 1.17415239 | 0.779771463 |
| Fasudil hydrochloride | Tocris Kinase Library | 1.17767468 | 0.919210843 |
| ISX 9 | Tocris StemCells Library | 1.178907406 | 0.94091135 |
| IOX 2 | Tocris Epigenetic Library | 1.182589665 | 0.885746326 |
| H 89 dihydrochloride | Tocris Epigenetic Library | 1.183769748 | 0.98017874 |
| Golgicide A | Custom Library | 1.190153144 | 0.762098247 |
| SB 203580 hydrochloride | Tocris Kinase Library | 1.191515434 | 0.784675388 |
| GSA-10 | Custom Library | 1.193882059 | 0.757997747 |
| BI 78D3 | Tocris Kinase Library | 1.193966245 | 0.854413601 |
| BIX 01294 | Tocris Epigenetic Library | 1.195646059 | 0.781881469 |
| Niraparib, MK -4827 | Custom Library | 1.198810299 | 0.75258868 |
| 2-ethylhexyl phthalate, MEHP | Custom Library | 1.205815292 | 0.744920555 |
| EPZ5676, pinometostat | Custom Library | 1.207119213 | 0.743496563 |
| SD 208 | Tocris Kinase Library | 1.214323924 | 0.725602387 |
| endo-IWR 1 | Tocris StemCells Library | 1.217912876 | 0.666782312 |
| VH 298 | Custom Library | 1.218938355 | 0.730626249 |
| ML 9 hydrochloride | Tocris Kinase Library | 1.221155309 | 0.868018262 |
| RN 1 dihydrochloride | Tocris Epigenetic Library | 1.221742904 | 0.7752818 |
| A 769662 | Tocris StemCells Library | 1.222813598 | 0.797183151 |
| Kartogenin | Tocris StemCells Library | 1.223536745 | 0.822193547 |
| GW 9662 | Custom Library | 1.223958021 | 0.725182687 |
| EO 1428 | Tocris Kinase Library | 1.224940397 | 0.931907303 |
| SAHA | Tocris Epigenetic Library | 1.228860996 | 0.819206059 |
| SB 202190 | Tocris Kinase Library | 1.237083265 | 0.738474063 |
| EX 527 | Tocris Epigenetic Library | 1.239301764 | 0.882228167 |
| L002 | Tocris Epigenetic Library | 1.242946397 | 0.820599895 |
| SB 203580 | Tocris StemCells Library | 1.24397278 | 0.76041459 |
| Cyclosporin A | Tocris StemCells Library | 1.244465608 | 0.794811632 |
| Zebularine | Tocris StemCells Library | 1.244500822 | 0.784950996 |
| Forskolin | Tocris StemCells Library | 1.245772642 | 0.866930001 |
| DSM265 | Custom Library | 1.24693257 | 0.700453037 |
| SC 514 | Tocris Kinase Library | 1.251322196 | 0.66031445 |
| TBB | Tocris Kinase Library | 1.256066751 | 0.710937675 |
| Compound 401 | Tocris Kinase Library | 1.256572305 | 0.80883037 |
| AMG 900 | Custom Library | 1.259478013 | 0.687082012 |
| SB 216763 | Tocris Kinase Library | 1.263616533 | 0.865157083 |
| Metformin hydrochloride | Tocris StemCells Library | 1.270776155 | 0.694146641 |
| Wnt Agonist II, SKL2001 | Custom Library | 1.27360077 | 0.672148464 |

| Compounds – 1 µM | Libraries | Fold Average | pValue |
|---|---|---|---|
| Tranylcypromine HCl | Tocris Epigenetic Library | 1.277953098 | 0.724718821 |
| NSC 3852 | Tocris Epigenetic Library | 1.293005208 | 0.703701987 |
| CTBP | Custom Library | 1.296884856 | 0.647814024 |
| Amiodarone hydrochloride | Tocris StemCells Library | 1.297667642 | 0.752935975 |
| 3-Aminobenzamide | Tocris Epigenetic Library | 1.301775832 | 0.791849908 |
| Purvalanol A | Tocris Kinase Library | 1.304497783 | 0.71854421 |
| 8-Bromo-cAMP, sodium salt | Tocris StemCells Library | 1.304732334 | 0.843977726 |
| ZM 306416 hydrochloride | Tocris Kinase Library | 1.312828769 | 0.683849812 |
| Mitoxantrone dihydrochloride | Tocris Epigenetic Library | 1.314236855 | 0.677617297 |
| Molidustat | Custom Library | 1.31700169 | 0.627091664 |
| Valproic acid, sodium salt | Tocris Epigenetic Library | 1.319574247 | 0.680693276 |
| UNC 1215 | Tocris Epigenetic Library | 1.328843565 | 0.791415774 |
| NSC 228155 | Custom Library | 1.333638519 | 0.610175985 |
| Tranylcypromine HCl | Tocris StemCells Library | 1.335865072 | 0.677692257 |
| GSK J4 | Tocris Epigenetic Library | 1.343340111 | 0.626438811 |
| NBI-31772 | Custom Library | 1.343714366 | 0.600032793 |
| Zebularine | Custom Library | 1.344758421 | 0.598985721 |
| Dorsomorphin 2HCl | Tocris StemCells Library | 1.345897235 | 0.495939554 |
| UNC 0642 | Tocris Epigenetic Library | 1.34712199 | 0.702865892 |
| Ki 8751 | Tocris Kinase Library | 1.351580971 | 0.606616664 |
| ZM 336372 | Tocris Kinase Library | 1.351626696 | 0.702713913 |
| SGC 0946 | Tocris Epigenetic Library | 1.352801767 | 0.5847914 |
| Dexamethasone | Tocris StemCells Library | 1.357455998 | 0.679229642 |
| MS 436 | Tocris Epigenetic Library | 1.357670627 | 0.70412017 |
| TCS 359 | Tocris Kinase Library | 1.360793411 | 0.625604303 |
| SU 5416 | Tocris Kinase Library | 1.361855754 | 0.565138557 |
| 5-Azacytidine | Tocris Epigenetic Library | 1.366657259 | 0.626874398 |
| Salermide | Tocris Epigenetic Library | 1.367820259 | 0.705413301 |
| Demethylasterriquinone B1 | Custom Library | 1.370459865 | 0.573490374 |
| GF 109203X | Tocris Kinase Library | 1.37494525 | 0.748506476 |
| MK-8617 | Custom Library | 1.379439568 | 0.56470764 |
| SU 4312 | Tocris Kinase Library | 1.381414589 | 0.694108819 |
| AICAR | Tocris StemCells Library | 1.38418874 | 0.6607597 |
| Neurodazine | Tocris StemCells Library | 1.388819884 | 0.626729188 |
| Fisetin | Tocris Epigenetic Library | 1.398185319 | 0.495777604 |
| GW 5074 | Tocris Kinase Library | 1.401422881 | 0.599301024 |
| Pifithrin-α hydrobromide | Tocris StemCells Library | 1.404005858 | 0.437995036 |
| Pyroxamide | Tocris Epigenetic Library | 1.416658914 | 0.53268338 |
| GSK J2 | Tocris Epigenetic Library | 1.417246018 | 0.645358845 |

| Compounds – 1 μM | Libraries | Fold Average | pValue |
|---|---|---|---|
| (-)-Terreic acid | Tocris Kinase Library | 1.418900929 | 0.500746078 |
| Valproic acid, sodium salt | Tocris StemCells Library | 1.425622093 | 0.642458031 |
| H 89 dihydrochloride | Tocris Kinase Library | 1.427219345 | 0.634846654 |
| RG 108 | Tocris Epigenetic Library | 1.429052206 | 0.676568194 |
| (R)-(+)-Bay K 8644 | Tocris StemCells Library | 1.433843357 | 0.415400878 |
| GW 441756 | Tocris Kinase Library | 1.442474864 | 0.528656329 |
| Rucaparib, AG-014699, PF-01367338 | Custom Library | 1.447408345 | 0.500441925 |
| KY 02111 | Tocris StemCells Library | 1.448419452 | 0.416544742 |
| PRT 4165 | Tocris Epigenetic Library | 1.449879889 | 0.699411334 |
| PF 670462 | Custom Library | 1.454014088 | 0.49441203 |
| Silmitasertib | Custom Library | 1.465387148 | 0.484125194 |
| I-CBP 112 | Tocris Epigenetic Library | 1.468053582 | 0.488551372 |
| Nicotinamide | Tocris StemCells Library | 1.468260186 | 0.590787698 |
| Arctigenin | Tocris Kinase Library | 1.478051233 | 0.505315487 |
| Pifithrin-μ | Tocris StemCells Library | 1.490469546 | 0.471651268 |
| GANT 58 | Tocris StemCells Library | 1.508515668 | 0.609554089 |
| A01 | Custom Library | 1.510010142 | 0.444923215 |
| Temozolomide | Tocris Epigenetic Library | 1.511371037 | 0.529695159 |
| PJ 34 hydrochloride | Tocris Epigenetic Library | 1.515915454 | 0.54393941 |
| Scriptaid | Tocris Epigenetic Library | 1.517776374 | 0.443303328 |
| Arcyriaflavin A | Tocris Kinase Library | 1.519991595 | 0.377046635 |
| N18-32-CG | Custom Library | 1.522692305 | 0.434126726 |
| Roxadustat | Custom Library | 1.525480141 | 0.431774433 |
| Purvalanol B | Tocris Kinase Library | 1.534006515 | 0.430535595 |
| KD 5170 | Tocris Epigenetic Library | 1.534104658 | 0.367695883 |
| SB 747651A 2HCl | Tocris Epigenetic Library | 1.544882545 | 0.515081624 |
| SB 431542 | Tocris StemCells Library | 1.549093998 | 0.428413724 |
| CD 437 | Tocris StemCells Library | 1.552088971 | 0.455759553 |
| Genistein | Tocris Kinase Library | 1.563230384 | 0.38154001 |
| P7C3 | Tocris StemCells Library | 1.580305296 | 0.48925856 |
| M 344 | Tocris Epigenetic Library | 1.601585917 | 0.378291499 |
| HA 1100 hydrochloride | Tocris Kinase Library | 1.604872715 | 0.391359844 |
| GW856553X, Losmapimod | Custom Library | 1.612034412 | 0.362532161 |
| anacardic acid | Custom Library | 1.613482865 | 0.36143684 |
| LY 364947 | Tocris Kinase Library | 1.616266138 | 0.39014518 |
| (±)-Blebbistatin | Tocris StemCells Library | 1.637702238 | 0.303150057 |
| GSK J1 | Tocris Epigenetic Library | 1.638486686 | 0.441995598 |
| EGCG | Tocris Epigenetic Library | 1.638722618 | 0.621909245 |
| PFI-1 | Custom Library | 1.647403607 | 0.336390893 |
| OXFBD02 | Custom Library | 1.659339965 | 0.327854081 |
| PHA 665752 | Tocris Kinase Library | 1.674904668 | 0.366092838 |

| Compounds – 1 μM | Libraries | Fold Average | pValue |
|---|---|---|---|
| AK 7 | Tocris Epigenetic Library | 1.707451118 | 0.258594429 |
| SR 3677 dihydrochloride | Tocris StemCells Library | 1.707612635 | 0.382961385 |
| Heclin | Custom Library | 1.712642191 | 0.291488217 |
| SB 218078 | Tocris Kinase Library | 1.725971297 | 0.255333319 |
| DAPT | Tocris StemCells Library | 1.734555105 | 0.167237046 |
| RepSox | Tocris StemCells Library | 1.744955678 | 0.37349209 |
| Cyclic Pifithrin-α hydrobromide | Tocris StemCells Library | 1.758481421 | 0.184748535 |
| P 22077 | Tocris Epigenetic Library | 1.787032635 | 0.228888167 |
| IG-4D-48 | Custom Library | 1.810628905 | 0.232052311 |
| AR-A014418 | Custom Library | 1.822415081 | 0.225538225 |
| D 4476 | Tocris Kinase Library | 1.830654141 | 0.259695077 |
| 10-DEBC hydrochloride | Tocris Kinase Library | 1.869223298 | 0.141136354 |
| Bithionoloxide | Custom Library | 1.887484861 | 0.191953188 |
| CAY10591,SirtAct | Custom Library | 1.927984789 | 0.173023892 |
| UC2288 | Custom Library | 1.932711258 | 0.17091069 |
| PD 407824 | Tocris Kinase Library | 1.95159385 | 0.074740107 |
| Bromosporine | Custom Library | 1.986711097 | 0.148139654 |
| GW 788388 | Tocris StemCells Library | 2.038311163 | 0.108811283 |
| Thiazovivin | Tocris StemCells Library | 2.085159961 | 0.177367254 |
| GSK621 | Custom Library | 2.180033033 | 0.085408554 |
| GW 843682X | Tocris Kinase Library | 2.324109571 | 0.06894118 |
| A 83-01 | Tocris StemCells Library | 2.337097563 | 0.032237358 |
| BAF312, Siponimod | Custom Library | 2.344484386 | 0.050978882 |
| XMU-MP-1 | Custom Library | 2.353663896 | 0.049468146 |
| ME0328 | Custom Library | 2.377332344 | 0.045747499 |
| SB 431542 | Tocris Kinase Library | 2.470182119 | 0.02937795 |
| C 646 | Tocris Epigenetic Library | 2.483932093 | 0.032037105 |

**Table S2. Compounds with no significant effect on LEC sprouting assay at 10 μM**

List of compounds tested at 10 μM that did not meet the predefined hit criteria (fold-change > 2.5 and $p < 0.05$). Data are presented as mean fold change from three independent experiments

| Compounds – 10 µM | Libraries | Fold Average | pValue |
|---|---|---|---|
| Wnt Agonist I | Custom Library | 0 | 0.278165656 |
| FTY720, Fingolimod | Custom Library | 0 | 0.278165656 |
| VR23 | Custom Library | 0 | 0.278165656 |
| WAY-262611 | Custom Library | 0 | 0.278165656 |
| b-AP15 | Custom Library | 0 | 0.278165656 |
| WP1130, Degrasyn | Custom Library | 0 | 0.278165656 |
| BI 78D3 | Tocris Kinase Library | 0 | 0.225447875 |
| Ro 08-2750 | Tocris Kinase Library | 0 | 0.225447875 |
| CLEFMA | Custom Library | 0.013504476 | 0.284668226 |
| UC2288 | Custom Library | 0.054674556 | 0.305132832 |
| Br-DIF-1 | Custom Library | 0.060770141 | 0.308245005 |
| AP 24534 | Tocris StemCells Library | 0.099088387 | 0.274746875 |
| Golgicide A | Custom Library | 0.127598725 | 0.343760328 |
| nultin-3A | Custom Library | 0.138083568 | 0.349564429 |
| PR-619 | Custom Library | 0.200851221 | 0.38562009 |
| SGI 1027 | Tocris Epigenetic Library | 0.202552157 | 0.333606825 |
| OTSSP167 | Custom Library | 0.204266285 | 0.387645528 |
| API-2 | Tocris Kinase Library | 0.234489411 | 0.353314381 |
| PF 03814735 | Tocris Epigenetic Library | 0.250936487 | 0.363745269 |
| TC-E 5003 | Tocris Epigenetic Library | 0.251538567 | 0.364130916 |
| ID 8 | Tocris StemCells Library | 0.277663378 | 0.381103483 |
| Afuresertib | Custom Library | 0.280771167 | 0.434736384 |
| Silmitasertib | Custom Library | 0.285284318 | 0.437615635 |
| PP 1 | Tocris Kinase Library | 0.313670608 | 0.405279989 |
| Sodium 4-Phenylbutyrate | Tocris StemCells Library | 0.344198818 | 0.426481277 |
| Triptolide | Tocris Epigenetic Library | 0.359195402 | 0.43712908 |
| AR-A014418 | Custom Library | 0.360064087 | 0.486914126 |
| JK 184 | Tocris StemCells Library | 0.383629983 | 0.454803166 |
| TBB | Tocris Kinase Library | 0.385813633 | 0.456402076 |
| PJ 34 hydrochloride | Tocris Epigenetic Library | 0.395181803 | 0.463297305 |
| KU-60019 | Custom Library | 0.419623729 | 0.528263349 |
| CP-466722 | Custom Library | 0.422324624 | 0.530180678 |
| SRT 1720 | Custom Library | 0.428099681 | 0.534291765 |
| Ro 31-8220 mesylate | Tocris Kinase Library | 0.436552404 | 0.494433765 |
| KU 55933 | Tocris Epigenetic Library | 0.464637479 | 0.516194244 |
| ME0328 | Custom Library | 0.492708752 | 0.581375918 |
| IMD 0354 | Tocris Kinase Library | 0.499941222 | 0.544238081 |
| XAV 939 | Tocris StemCells Library | 0.499941222 | 0.544238081 |
| SB 218078 | Tocris Kinase Library | 0.51589726 | 0.557157886 |
| GSK J4 | Tocris Epigenetic Library | 0.520834998 | 0.561186047 |
| Metformin hydrochloride | Tocris StemCells Library | 0.529937105 | 0.568648593 |

| Compounds – 10 µM | Libraries | Fold Average | pValue |
|---|---|---|---|
| Dorsomorphin dihydrochloride | Tocris StemCells Library | 0.554504383 | 0.589023927 |
| PI 828 | Tocris Kinase Library | 0.558630253 | 0.592478818 |
| SC 514 | Tocris Kinase Library | 0.560446904 | 0.594002948 |
| IG-4D-51 | Custom Library | 0.560707934 | 0.632962228 |
| LY 294002 hydrochloride | Tocris Kinase Library | 0.563323935 | 0.596420339 |
| PD 98059 | Tocris Kinase Library | 0.575319384 | 0.606547468 |
| LY 294002 hydrochloride | Tocris StemCells Library | 0.578509213 | 0.609253286 |
| PD 198306 | Tocris Kinase Library | 0.584004092 | 0.6139276 |
| P7C3 | Tocris StemCells Library | 0.593794066 | 0.622293684 |
| 5-Azacytidine | Tocris StemCells Library | 0.597012454 | 0.625055099 |
| SP 600125 | Tocris Kinase Library | 0.601372992 | 0.628804422 |
| IQ 1 | Tocris StemCells Library | 0.60941073 | 0.635740947 |
| TC-H 106 | Tocris Epigenetic Library | 0.613061188 | 0.638901858 |
| PD 173074 | Tocris StemCells Library | 0.620517819 | 0.645379202 |
| Retinoic acid | Tocris StemCells Library | 0.627472572 | 0.651445199 |
| TCS HDAC6 20b | Tocris Epigenetic Library | 0.65251012 | 0.673472059 |
| CI 994 | Tocris Epigenetic Library | 0.658720134 | 0.678980365 |
| BIX 01294 | Tocris StemCells Library | 0.662729673 | 0.682545656 |
| PD 0325901 | Tocris StemCells Library | 0.666472024 | 0.685879648 |
| TPCA-1 | Tocris Kinase Library | 0.69028446 | 0.70723774 |
| IKK 16 | Tocris Kinase Library | 0.693394774 | 0.710044709 |
| ZM 39923 hydrochloride | Tocris Kinase Library | 0.706853961 | 0.722237713 |
| L002 | Tocris Epigenetic Library | 0.70849007 | 0.723724698 |
| KU 55933 | Tocris Kinase Library | 0.715763502 | 0.730348141 |
| U0126 | Tocris Epigenetic Library | 0.717185274 | 0.731645461 |
| 4SC-101, Vidofludimus | Custom Library | 0.725571415 | 0.765364937 |
| NSC 228155 | Custom Library | 0.7319824 | 0.770688621 |
| ZM 447439 | Tocris Epigenetic Library | 0.732015852 | 0.745220728 |
| CD 437 | Tocris StemCells Library | 0.739888201 | 0.752459836 |
| Wnt Agonist II, SKL2001 | Custom Library | 0.751838636 | 0.787245691 |
| ER 27319 maleate | Tocris Kinase Library | 0.761937873 | 0.772849711 |
| EX 527 | Tocris Epigenetic Library | 0.769207289 | 0.77960725 |
| PD 98059 | Tocris StemCells Library | 0.770601755 | 0.780905345 |
| KU 60019 | Tocris Epigenetic Library | 0.771236382 | 0.7814961 |
| IBMX | Tocris StemCells Library | 0.776179233 | 0.78610413 |
| Ceramide | Tocris StemCells Library | 0.788246077 | 0.79738363 |
| A 769662 | Tocris StemCells Library | 0.790148814 | 0.799166123 |
| ZM 336372 | Tocris Kinase Library | 0.793032345 | 0.801869654 |
| LY 303511 | Tocris Epigenetic Library | 0.802212347 | 0.810491912 |
| Thioridazine hydrochloride | Tocris StemCells Library | 0.802493824 | 0.810756523 |
| GANT 61 | Tocris StemCells Library | 0.806606448 | 0.814627196 |

| Compounds – 10 µM | Libraries | Fold Average | pValue |
|---|---|---|---|
| BIX 01294 | Tocris Epigenetic Library | 0.811599345 | 0.819332816 |
| NU 7026 | Tocris Kinase Library | 0.821222964 | 0.828420264 |
| SP 600125 | Tocris StemCells Library | 0.82247598 | 0.829604857 |
| SAG | Custom Library | 0.851889199 | 0.872016477 |
| ISX 9 | Tocris StemCells Library | 0.854455932 | 0.859971002 |
| Temozolomide | Tocris Epigenetic Library | 0.85926058 | 0.864552734 |
| ZM 306416 hydrochloride | Tocris Kinase Library | 0.865408803 | 0.870421711 |
| Memantine (hydrochloride) | Custom Library | 0.867269912 | 0.885207967 |
| ZM 447439 | Tocris Kinase Library | 0.872403042 | 0.877107308 |
| SL 327 | Tocris Kinase Library | 0.874001347 | 0.878636342 |
| Prostaglandin E2 | Tocris StemCells Library | 0.880315279 | 0.884680974 |
| BMS 453 | Tocris StemCells Library | 0.881087945 | 0.885420827 |
| DCP-LA, FR 236924 | Custom Library | 0.886992686 | 0.902170379 |
| GSK J1 | Tocris Epigenetic Library | 0.888729216 | 0.892746111 |
| Demethylasterriquinone B1 | Custom Library | 0.889400061 | 0.904244229 |
| GW856553X, Losmapimod | Custom Library | 0.898190259 | 0.911822004 |
| Cyclic Pifithrin-α hydrobromide | Tocris StemCells Library | 0.899568268 | 0.903152739 |
| PCI 34051 | Tocris Epigenetic Library | 0.903028069 | 0.906477901 |
| PFI 3 | Tocris Epigenetic Library | 0.9066747 | 0.909984572 |
| 10-DEBC hydrochloride | Tocris Kinase Library | 0.915849774 | 0.918814976 |
| SU 5416 | Tocris Kinase Library | 0.923609718 | 0.926291739 |
| 1-EBIO | Tocris StemCells Library | 0.939106708 | 0.941241041 |
| PQ 401 | Tocris Kinase Library | 0.940232629 | 0.942328143 |
| IOX 1 | Tocris Epigenetic Library | 0.946611652 | 0.948488754 |
| ZM 449829 | Tocris Kinase Library | 0.953295705 | 0.954947554 |
| AM 580 | Tocris StemCells Library | 0.954881722 | 0.956480171 |
| Tranylcypromine HCl | Tocris StemCells Library | 0.960371412 | 0.961787388 |
| RN 1 dihydrochloride | Tocris Epigenetic Library | 0.962053646 | 0.963413837 |
| ML 9 hydrochloride | Tocris Kinase Library | 0.962546552 | 0.963890907 |
| PS 48 | Tocris StemCells Library | 0.966979493 | 0.968178325 |
| PRT 4165 | Tocris Epigenetic Library | 0.973106505 | 0.974105281 |
| Barasertib-hQPA | Custom Library | 0.975362767 | 0.978619568 |
| Compound 401 | Tocris Kinase Library | 0.976266512 | 0.977163179 |
| UNC 0646 | Tocris Epigenetic Library | 0.980219464 | 0.980988197 |
| Olomoucine | Tocris Kinase Library | 0.985833045 | 0.986421307 |
| WIKI4 | Tocris StemCells Library | 0.998384574 | 0.998571173 |
| Daminozide | Tocris Epigenetic Library | 1.007751029 | 0.992361954 |
| Cardiogenol C hydrochloride | Tocris StemCells Library | 1.008679156 | 0.991463336 |
| BIBX 1382 dihydrochloride | Tocris Kinase Library | 1.020689288 | 0.979839158 |
| SGC 0946 | Tocris Epigenetic Library | 1.021608963 | 0.978949181 |
| H 89 dihydrochloride | Tocris Epigenetic Library | 1.026856894 | 0.973871463 |

| Compounds – 10 µM | Libraries | Fold Average | pValue |
|---|---|---|---|
| SL 327 | Tocris Epigenetic Library | 1.035598283 | 0.965415359 |
| FPA 124 | Tocris Kinase Library | 1.040657392 | 0.960523019 |
| FK-506 | Custom Library | 1.040902746 | 0.964511936 |
| ZM 323881 hydrochloride | Tocris Kinase Library | 1.044912799 | 0.956409288 |
| LFM-A13 | Tocris Kinase Library | 1.063662842 | 0.938298891 |
| Sodium 4-Phenylbutyrate | Tocris Epigenetic Library | 1.072774963 | 0.929508207 |
| AZD9496 | Custom Library | 1.07647762 | 0.933702389 |
| Nicotinamide | Tocris StemCells Library | 1.089033235 | 0.913845865 |
| Tenovin -1 | Tocris Epigenetic Library | 1.100158545 | 0.903147294 |
| Niraparib, MK-4827 | Custom Library | 1.102234865 | 0.911455012 |
| RG 108 | Tocris StemCells Library | 1.104119495 | 0.899342388 |
| VH 298 | Custom Library | 1.1070511 | 0.907302158 |
| UNC0642 | Custom Library | 1.11462823 | 0.90077441 |
| SB 415286 | Tocris Kinase Library | 1.132331642 | 0.872314347 |
| Splitomicin | Tocris Epigenetic Library | 1.140531138 | 0.864485318 |
| SC79 | Custom Library | 1.140537658 | 0.878507381 |
| Retinoic acid | Tocris Epigenetic Library | 1.144468086 | 0.860731281 |
| (-) MK 801 | Custom Library | 1.145623779 | 0.874146742 |
| CGK 733 | Tocris Kinase Library | 1.146588695 | 0.858710299 |
| CGP 57380 | Tocris Kinase Library | 1.146642462 | 0.858658849 |
| Decitabine | Tocris Epigenetic Library | 1.151938935 | 0.853615618 |
| Troglitazone | Tocris StemCells Library | 1.155005752 | 0.850698229 |
| DAPT | Tocris StemCells Library | 1.155294446 | 0.850423718 |
| IG-4D-44 | Custom Library | 1.158715217 | 0.862942025 |
| N18-32-CG | Custom Library | 1.164141924 | 0.858305064 |
| GSK J5 | Tocris Epigenetic Library | 1.172163462 | 0.834414333 |
| SBHA | Tocris Epigenetic Library | 1.17229728 | 0.834287745 |
| PP 2 | Tocris Kinase Library | 1.174264492 | 0.832425421 |
| UNC 1215 | Tocris Epigenetic Library | 1.176631352 | 0.830185948 |
| Lisinopril | Custom Library | 1.195352543 | 0.831739165 |
| Kartogenin | Tocris StemCells Library | 1.197543356 | 0.810459621 |
| UNC 0638 | Tocris Epigenetic Library | 1.202436271 | 0.805861005 |
| A 366 | Tocris Epigenetic Library | 1.20526743 | 0.803203392 |
| NBI-31772 | Custom Library | 1.212701604 | 0.817053885 |
| anacardic acid | Custom Library | 1.215088127 | 0.81503877 |
| SU 4312 | Tocris Kinase Library | 1.21980462 | 0.789592969 |
| 1,2,3,4,5,6-Hexabromocyclohexane | Tocris Kinase Library | 1.224960388 | 0.784781535 |
| 5-Azacytidine | Tocris Epigenetic Library | 1.228697 | 0.78129896 |
| Purvalanol B | Tocris Kinase Library | 1.2286988 | 0.781297098 |
| AG 490 | Tocris Kinase Library | 1.228822085 | 0.781182546 |
| (±)-Blebbistatin | Tocris StemCells Library | 1.231578424 | 0.778616483 |

| Compounds – 10 µM | Libraries | Fold Average | pValue |
|---|---|---|---|
| Bithionoloxide | Custom Library | 1.239565801 | 0.794444008 |
| RG 108 | Tocris Epigenetic Library | 1.247422513 | 0.763915995 |
| GANT 58 | Tocris StemCells Library | 1.249271413 | 0.762205843 |
| NSC 3852 | Tocris Epigenetic Library | 1.2544208 | 0.757449042 |
| Y-27632 dihydrochloride | Tocris StemCells Library | 1.255883968 | 0.756099016 |
| UNC 926 hydrochloride | Tocris Epigenetic Library | 1.274921939 | 0.738604884 |
| (±)-Bay K 8644 | Tocris StemCells Library | 1.276748254 | 0.736933577 |
| GW 9662 | Custom Library | 1.287943862 | 0.754178775 |
| Aminopurvalanol A | Tocris Kinase Library | 1.290766704 | 0.724147232 |
| IOX 2 | Tocris Epigenetic Library | 1.298814074 | 0.716841595 |
| Iressa | Tocris Kinase Library | 1.306121808 | 0.710229873 |
| GSK 650394 | Tocris Kinase Library | 1.312408961 | 0.704558749 |
| GF 109203X | Tocris Kinase Library | 1.313157503 | 0.703884847 |
| Exo1 | Custom Library | 1.315740403 | 0.731339161 |
| Cyclosporin A | Tocris StemCells Library | 1.31837842 | 0.699189356 |
| EGCG | Tocris Epigenetic Library | 1.33241131 | 0.892309986 |
| Amiodarone hydrochloride | Tocris StemCells Library | 1.334274859 | 0.684965498 |
| Kaempferol | Tocris Epigenetic Library | 1.337178066 | 0.682379505 |
| Dexamethasone | Tocris StemCells Library | 1.354432026 | 0.667089663 |
| Resveratrol | Tocris Epigenetic Library | 1.357083906 | 0.664751566 |
| CTBP | Custom Library | 1.358557117 | 0.696632627 |
| CH 223191 | Tocris StemCells Library | 1.361796457 | 0.660605078 |
| SB 216763 | Tocris Kinase Library | 1.365214549 | 0.657603727 |
| Genistein | Tocris Kinase Library | 1.368718798 | 0.654532897 |
| Fasudil hydrochloride | Tocris Kinase Library | 1.368759769 | 0.654497 |
| 8-Bromo-cAMP, sodium salt | Tocris StemCells Library | 1.371922306 | 0.651730685 |
| Tranylcypromine HCl | Tocris Epigenetic Library | 1.37245245 | 0.651266943 |
| PD 407824 | Tocris Kinase Library | 1.377818367 | 0.646585299 |
| endo-IWR 1 | Tocris StemCells Library | 1.379411266 | 0.64519815 |
| NSC 693868 | Tocris Kinase Library | 1.397489082 | 0.629544476 |
| Lomeguatrib | Tocris Epigenetic Library | 1.406299621 | 0.621974588 |
| NVP DPP 728 2HCl | Tocris StemCells Library | 1.411549131 | 0.617483428 |
| Roxadustat | Custom Library | 1.411956453 | 0.654242231 |
| PHA 665752 | Tocris Kinase Library | 1.41220735 | 0.616921185 |
| GW 5074 | Tocris Kinase Library | 1.424880073 | 0.606142442 |
| (R)-(+)-Bay K 8644 | Tocris StemCells Library | 1.426115088 | 0.605096546 |
| Ki 8751 | Tocris Kinase Library | 1.438221075 | 0.594887206 |
| GSK J2 | Tocris Epigenetic Library | 1.456261473 | 0.579822201 |
| IPA 3 | Tocris StemCells Library | 1.461375579 | 0.57558416 |
| UNC 0642 | Tocris Epigenetic Library | 1.461617786 | 0.575383989 |
| BIO | Tocris StemCells Library | 1.484108996 | 0.556925814 |

| Compounds – 10 µM | Libraries | Fold Average | pValue |
|---|---|---|---|
| PluriSIn 1 | Tocris StemCells Library | 1.488760314 | 0.553145255 |
| KY 02111 | Tocris StemCells Library | 1.498870435 | 0.544970601 |
| Fluoxetine hydrochloride | Tocris StemCells Library | 1.499363011 | 0.544574083 |
| IDE 1 | Tocris StemCells Library | 1.505966538 | 0.539269519 |
| EX 527, Selisistat | Custom Library | 1.509930085 | 0.579414717 |
| Valproic acid, sodium salt | Tocris StemCells Library | 1.511270428 | 0.535028364 |
| NH 125 | Tocris Kinase Library | 1.512133575 | 0.534339277 |
| Arcyriaflavin A | Tocris Kinase Library | 1.527101887 | 0.522468707 |
| GSK2656157 | Custom Library | 1.527898041 | 0.566146144 |
| P 22077 | Tocris Epigenetic Library | 1.533606908 | 0.517352268 |
| Zebularine | Custom Library | 1.533647154 | 0.56193188 |
| Neurodazine | Tocris StemCells Library | 1.561734312 | 0.495533305 |
| 20(S)-Hydroxycholesterol | Custom Library | 1.565488406 | 0.538876143 |
| GSK 429286 | Tocris StemCells Library | 1.579786899 | 0.481793776 |
| Valproic acid, sodium salt | Tocris Epigenetic Library | 1.596530307 | 0.469238388 |
| GW 441756 | Tocris Kinase Library | 1.604349114 | 0.463438245 |
| UNC1999 | Custom Library | 1.60820292 | 0.50872105 |
| H 89 dihydrochloride | Tocris Kinase Library | 1.609906361 | 0.459340117 |
| Ryuvidine | Tocris Kinase Library | 1.620623315 | 0.451494898 |
| 2-ethylhexyl phthalate, MEHP | Custom Library | 1.630427638 | 0.493394085 |
| C 646 | Tocris Epigenetic Library | 1.631198878 | 0.443828226 |
| AICAR | Tocris StemCells Library | 1.64285986 | 0.435461858 |
| MLR 1023 | Custom Library | 1.645929849 | 0.482853383 |
| MC 1568 | Tocris Epigenetic Library | 1.65190659 | 0.429034525 |
| GW 583340 dihydrochloride | Tocris Kinase Library | 1.653076459 | 0.428207576 |
| DMH-1 | Tocris StemCells Library | 1.672967206 | 0.41428779 |
| Y-27632 dihydrochloride | Tocris Kinase Library | 1.678013258 | 0.410799748 |
| Sirtinol | Tocris Epigenetic Library | 1.685109029 | 0.405924357 |
| U0126 | Tocris Kinase Library | 1.69801853 | 0.397144238 |
| GSA-10 | Custom Library | 1.700830655 | 0.446542387 |
| AZ1366 | Custom Library | 1.704510998 | 0.44416621 |
| Pifithrin-α hydrobromide | Tocris StemCells Library | 1.708835491 | 0.389876635 |
| L-Ascorbic acid | Tocris StemCells Library | 1.713858058 | 0.386529717 |
| Purvalanol A | Tocris Kinase Library | 1.750135976 | 0.362881217 |
| I-CBP 112 | Tocris Epigenetic Library | 1.755221138 | 0.359640386 |
| 7-Nitroindazole | Custom Library | 1.774153844 | 0.400598262 |
| PFI 1 | Tocris Epigenetic Library | 1.789914731 | 0.338019773 |
| UNC 0224 | Tocris Epigenetic Library | 1.793292567 | 0.335960308 |
| RepSox | Tocris StemCells Library | 1.821115827 | 0.319306442 |
| MS 436 | Tocris Epigenetic Library | 1.828393126 | 0.315041454 |
| GW 843682X | Tocris Kinase Library | 1.829617543 | 0.314327622 |

| Compounds – 10 µM | Libraries | Fold Average | pValue |
|---|---|---|---|
| Salermide | Tocris Epigenetic Library | 1.83981359 | 0.308424255 |
| 3-Aminobenzamide | Tocris Epigenetic Library | 1.85891597 | 0.297563794 |
| SB 239063 | Tocris Kinase Library | 1.862541117 | 0.295531946 |
| IG-4D-55 | Custom Library | 1.864790167 | 0.347967518 |
| TCS 359 | Tocris Kinase Library | 1.877526649 | 0.287232343 |
| SR 3677 dihydrochloride | Tocris StemCells Library | 1.878650912 | 0.286616242 |
| Gallocyanine, IIIC3 | Custom Library | 1.889079273 | 0.334657555 |
| Fisetin | Tocris Epigenetic Library | 1.928747428 | 0.260063297 |
| SNS 314 mesylate | Tocris Epigenetic Library | 1.940280816 | 0.254200232 |
| BAF312, Siponimod | Custom Library | 1.966771049 | 0.294352171 |
| Zebularine | Tocris StemCells Library | 1.969781711 | 0.239625169 |
| MLN9708 | Custom Library | 1.986926665 | 0.284459095 |
| EO 1428 | Tocris Kinase Library | 1.988237536 | 0.230813004 |
| DSM265 | Custom Library | 1.995431142 | 0.280354093 |
| EC 23 | Tocris StemCells Library | 1.996416352 | 0.226982722 |
| LY 364947 | Tocris Kinase Library | 1.997951423 | 0.226268784 |
| Molidustat | Custom Library | 2.010058654 | 0.273389584 |
| SD 208 | Tocris Kinase Library | 2.029610626 | 0.211904006 |
| SB 203580 | Tocris StemCells Library | 2.061848357 | 0.197967912 |
| BIO | Tocris Kinase Library | 2.062422144 | 0.197726304 |
| Zebularine | Tocris Epigenetic Library | 2.072579185 | 0.193481729 |
| PFI-1 | Custom Library | 2.074173085 | 0.244284904 |
| Arctigenin | Tocris Kinase Library | 2.079830764 | 0.190492847 |
| Pyroxamide | Tocris Epigenetic Library | 2.13468181 | 0.168981047 |
| Forskolin | Tocris Epigenetic Library | 2.208821037 | 0.142878786 |
| SC-10 | Custom Library | 2.223629104 | 0.185181244 |
| GSK621 | Custom Library | 2.232307326 | 0.182111769 |
| IG-4D-48 | Custom Library | 2.248378696 | 0.176529098 |
| Rucaparib, AG-014699, PF-01367338 | Custom Library | 2.250705592 | 0.175731435 |
| Doxycycline hyclate | Tocris StemCells Library | 2.289066513 | 0.11824856 |
| (-)-Terreic acid | Tocris Kinase Library | 2.295842795 | 0.116331935 |
| HA 1100 hydrochloride | Tocris Kinase Library | 2.352639166 | 0.101215001 |
| SB 202190 | Tocris Kinase Library | 2.378386233 | 0.094900156 |
| 3BDO | Custom Library | 2.389673969 | 0.132890049 |
| 1-Naphthyl PP1 | Tocris Kinase Library | 2.391979771 | 0.0916961 |
| EPZ5676, pinometostat | Custom Library | 2.50572474 | 0.103809159 |
| Forskolin | Tocris StemCells Library | 2.57486328 | 0.056482639 |
| (-)-JQ1 | Custom Library | 2.621113649 | 0.080216735 |
| m-3M3FBS | Custom Library | 2.653167449 | 0.074510969 |
| LY2228820 | Custom Library | 2.65498528 | 0.074197797 |
| 6,2',4'-Trimethoxyflavone | Custom Library | 2.721096731 | 0.063527691 |

**Table S3. Kinase targets of Kenpaullone according to the IUPHAR database** List of kinases reported to be inhibited by Kenpaullone. The percentage of residual kinase activity remaining at 0.5 μM and 10 μM kenpaullone is reported as well as reference small-molecule inhibitors for each kinase.

| Target | % Activity remaining at 0.5μM | % Activity remaining at 10μM | Small Molecule inhibitor |
|---|---|---|---|
| CDK9 | 31.3 | 1 | Wogonin and NVP-2 |
| CDK2 | NA | 2 | Dinaciclib |
| NUAK1 | 14.6 | 2 | WZ4003 |
| ACK1 | 38.8 | 3 | Aim-100 |
| MAP4K4 | 6.6 | NA | DMX-5804 |
| FLT3 | 47.7 | 34 | Giltertinib |
| MLK3 | 49.4 | NA | URMC-099 |

**Table S4. MAP4K4 inhibitors potency** Reported MAP4K4 IC50 in presence of selected known MAP4K4 inhibitors and kenpaullone

| | Structure | Reported MAP4K4 $IC_{50}$ (nM) | Source reference |
|---|---|---|---|
| DMX- 5804 | 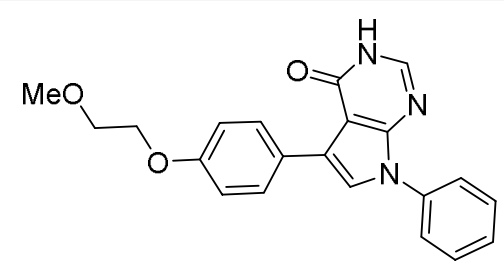 | 3 | Fiedler LR, *et al. Cell Stem Cell* **2019** [10.1016/j.stem.2019.01.013] |
| MAP4K4-IN-3 | 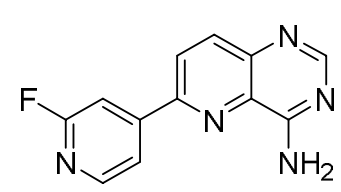 | 3.7 | Read J, *et al. Bioorg. Med. Chem. Lett.* **2019** [10.1016/j.bmcl.2019.05.032] |
| PF-06260933 | 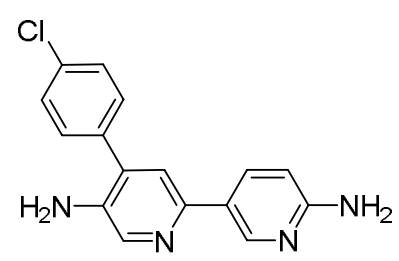 | 3.7 | Ammirati M, *et al. ACS Med. Chem. Lett.* **2015** [10.1021/acsmedchemlett.5b00215] |
| GNE-495 | 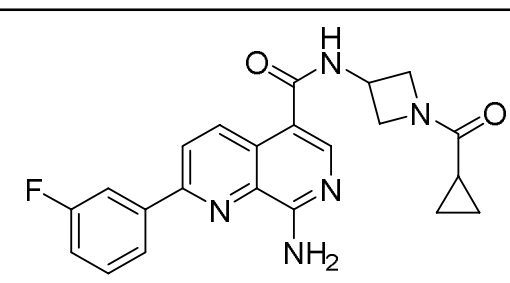 | 14.9 | Ndubaku CO *et al. ACS Med. Chem. Lett.* **2015** [10.1021/acsmedchemlett.5b00174] |
| Kenpaullone | 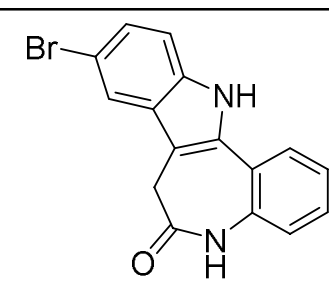 | 30 | Fiedler LR, *et al. Cell Stem Cell* **2019** [10.1016/j.stem.2019.01.013] |

**Table S5. List of qPCR primers**

| Gene | Forward Primer 5'-3' | Reverse Primer 5'-3' |
|---|---|---|
| 36B4 | CTACAACCCTGAAGAAGTGCTTG | CAATCTGCAGACAGACACTGG |
| ATF2 | GGTAGCGGATTGGTTAGGACTC | TGCTCTTCTCCGACGACCACTT |
| GSK3β | CCTCTGGCTACCATCCTTATTC | TTATTGGTCTGTCCACGGTCTC |
| JUN | CCTTGAAAGCTCAGAACTCGGAG | TGCTGCGTTAGCATGAGTTGGC |
| MAP4K4 | TCTGGCTTGTTATGGAGTTCTGTG | TGTTCTTCACAAGGTCTGTAATGGA |
| MYC | CCTGGTGCTCCATGAGGAGAC | CAGACTCTGACCTTTTGCCAGG |
| VEGFD | GACTGGAAGCTGTGGAGATGCA | GGCTGCACTGAGTTCTTTGCCA |

## Table S6. List of antibodies

| Target | Provider | Reference | Technique | Dilution/Final conc |
|---|---|---|---|---|
| GAPDH | Abcam | ab8245 | WesternBlot | 1/2000 |
| β-Catenin | Abcam | ab6302 | WesternBlot | 1/1000 |
| GSK3β | Cell Signaling | 9315 | WesternBlot | 1/1000 |
| p44/42 MAPK (Erk1/2) | Cell Signaling | 9102 | WesternBlot | 1/1000 |
| Phospho-p44/42 MAPK (Erk1/2) | Cell Signaling | 9101 | WesternBlot | 1/1000 |
| Akt (pan) | Cell Signaling | 4691 | WesternBlot | 1/1000 |
| Phospho-Akt (Ser473) | Cell Signaling | 4058 | WesternBlot | 1/1000 |
| Biotin | Cell Signaling | 5597 | WesternBlot | 1/1000 |
| MAP4K4 | Proteintech | 55247-1-AP | WesternBlot | 1/1000 |
| MAP4K4 | Proteintech | 55247-1-AP | Immunostaining | 1/100 |
| CD31 | R&D Systems | AF3628 | Immunostaining | 1/100 |
| Podoplanin | LS Bio | LS-C179446-LSP | Immunostaining | 1/100 |
| Lyve1 | Angiobio | 11-034 | Immunostaining | 1/100 |
| Phalloidin-488 | Invitrogen | A12379 | Immunostaining | 1/1000 |
| ER-Tracker Green | Invitrogen | E34251 | Immunostaining | 10 µM |
| BP Fluor 647 DBCO | BroadPharma | BP-25584 | Immunostaining | 10 µM |
| β-Catenin | Abcam | ab6302 | Immunostaining | 1/100 |
| Dapi | Sigma | D9542 | Immunostaining | 1/5000 |
| 7-AAD Viability Staining Solution | BioLegend | 420403 | Flow Cytometry | 1/100 |
| CD45-AlexaFluor 647 | BioLegend | 103124 | Flow Cytometry | 1/100 |
| CD11b-FITC | BioLegend | 101206 | Flow Cytometry | 1/100 |
| Ly6G-Brillant Violet 421 | BioLegend | 127636 | Flow Cytometry | 1/100 |
| F4/80-PE | BioLegend | 123110 | Flow Cytometry | 1/100 |
| CD11c-APC/Cy7 | BioLegend | 117323 | Flow Cytometry | 1/100 |

**Detailed Materials and Methods**

**Cell Culture**

Human Primary Lymphatic Endothelial Cells (LEC) (Lonza Cat#CC-2543, Batch No: 0000649177) were cultured at 37°C, 5% $CO_2$ in EGM-2 MV Endothelial Cell Growth Medium-2 (Lonza Cat#CC-3202) following manufacturer's instructions. Media was changed every other day. Cells were dissociated with 0.025% trypsin/EDTA (Lonza Cat#CC-2543) when they reached 80% confluency and cell number was determined using a Countess™ Automated Cell Counter in presence of trypan blue (ThermoFisher Cat#C10228). Cells were plated in flasks with a minimum density of 8,000 cells/cm² and were used for experiments from passage 6 to 9.

**Sprouting assay and Screening**

After dissociation, cells were neutralized, resuspended and plated in an incomplete EGM-2 Endothelial Cell Growth Medium-2 (EGM-I) (Lonza Cat#CC-3162). EGM-I consisted of EBM-2 Basal Medium supplemented with 10 mL FBS, 0.20 mL Hydrocortisone, 0.5 mL R3-IGF-1, 0.5 mL Ascorbic Acid, 0.5 mL GA-1000, 0.5 mL Heparin. LEC-spheroids were generated using AggreWell™400 plates (Stem Cell Technologies #34425) following manufacturer instructions. Briefly, AggreWell™400 plates were pre-treated with anti-adherence solution (Stem Cell Technologies #07010) and centrifuged 5 min, 1000 x g. The anti-adherence solution was removed and wells rinsed with EGM-I media. The media was then replaced with fresh EGM-I and top up v/v with fresh EGM-I containing cells (400 cells/microwell) by swirling stripette tip while distributing. Plates were centrifuged 3 min, 100 x g and incubated overnight at 37°C, 5% CO2. Spheroids were then harvested by removing and dispensing firmly back the media to dislodge them. Spheroids were then centrifuged 3 min at 200 x g and resuspended in EGM-I at the desired density before embedding in Collagen type1. Collagen type 1 solution was prepared on ice by using VitroCol® collagen (Advanced Biomatrix Cat#5007; 3 mg/mL stock) diluted to a final concentration of 1 mg/mL. After pH neutralisation and isotonicity maintenance by adding NaOH and 10X PBS respectively, spheroids resuspended in EGM-I medium were added, such that the final mixture contained 1 mg/mL collagen and 600 spheroids/mL. 25 µL of the mix was dispensed into each well of a 384-well CellCarrier Ultra microplate (Perkin Elmer #6057302) and plates were incubated at 37°C for 1 hour to allow the collagen matrix to set. After polymerisation, 40 µL of compound in

EGM-I was added to each well and plates were returned to the incubator overnight. The 2 first and last columns of each plate were treated with positive and negative controls to enable plate quality assessment and Z'-factor calculation. Spheroids seeding and treatment were performed using a 16 Channel VIAFLO Pipettes (Integra). Stem cell Toolbox (Tocris #5060), Epigenetics Toolbox (Tocris #5268), Kinase Inhibitor Toolbox (Tocris #3514) and custom-made compound set were used for the screening.

For the dose response assays, an 8-point serial dilution was prepared in EGM-I, starting from a top concentration of 50 μM for small compounds or 200 ng/mL for human recombinant proteins and diluted 1:3 across the range. Each condition was plated in duplicate.

**Spheroid staining and imaging**

24h after treatment, spheroids were fixed with direct addition of pre-warmed 4% PFA (ChemCruz Cat#sc-281692) for 15 minutes at room temperature. PFA and media were then replaced with 0.1% triton-X in PBS solution for 15 min. This was then changed by the staining solution composed of 0.1% triton-X in PBS, 1:1000 Alexa Fluor™ 488 Phalloidin (ThermoFisher Cat#A12379) and 1:10000 DAPI (Sigma Cat#D9542) for 40 min. Wells were then washed twice with PBS for 5 min each and plates were stored at 4°C until ready to image. All these steps were performed using a 16 Channel VIAFLO Pipettes (Integra) paired with an Assist Plus pipetting robot (Integra). Wells were imaged using an ImageXpress Pico Automated Cell Imaging System (Molecular Devices) on a 4X objective, using FITC and DAPI channels.

**Protein extraction and Immunoblot**

*Spheroids*: 10,000 spheroids per conditions were seeded and embedded in 3 mL of collagen solution as described above in 6 cm tissue culture dishes. Collagen was allowed to solidify for 1 hour in incubator, and then plates were treated with 3 mL of compound in EGM-I. Spheroids were treated for the indicated time, and then media was aspirated and plate washed with PBS. 100 μL collagenase (10,000 units/mL - StemCell Technologies Cat#07419) was added to each plate and incubated at 37°C while shaking for 10 min. Dissolved collagen was collected and remaining cells were scraped in PBS to be added to tubes and then centrifuged 500 x g, 5 min. Pellets were washed twice in PBS and then resuspended and lysed for 30 min at 4°C in 1X RIPA buffer (ThermoFisher Cat#89900) with 1:20 dilution protease inhibitor (ThermoFisher Cat#A32961) and 1:100 dilution

phosphatase inhibitor cocktail (ThermoFisher Cat#78441). Samples were then centrifuged 13,000 x g for 30 minutes at 4°C. Protein concentrations were measured by BCA assay (ThermoFisher Cat#23227) and protein extract was then supplemented with 4X Laemmli solution and 2-Mercaptoethanol (5 µL/100 µL protein lysate – Sigma Cat#M6250).

*Monolayer:* Cells were washed once with ice-cold PBS and lysed for 30 min at 4°C in 1X RIPA buffer with 1:20 dilution protease inhibitor and 1:100 dilution phosphatase inhibitor cocktail. Samples were then centrifuged 13,000 x g for 30 minutes at 4°C. Protein concentrations were measured by BCA assay (ThermoFisher Cat#23227) and protein extract was then supplemented with 4X Laemmli solution and 2-Mercaptoethanol (5 µL/100 µL protein lysate – Sigma Cat#M6250). For signalling pathway time-course experiements, LECs were serum-starved overnight in EGM2-MV media lacking supplements. Cells were then stimulated with VEGF-C or kenpaullone for the indicated time points before being washed with ice-cold PBS and lysed for proteins extraction as described above.

*Immunoblot:* Samples were heated to 95°C for 10 minutes and 15 µg protein were loaded on a 4 – 20% Tris- Glycine gel (ThermoFisher Cat#XP04200BOX). Gel was transferred onto a PVDF membrane (Bio-Rad Cat#1704156) using Trans-Blot Turbo Transfer System. Membranes were blocked in 5 % skim milk in TBS + 0.1% Tween–20 (TBST) for 1 hour followed by primary antibody incubation in 5 % skim milk in TBST at 4°C overnight. Membranes were then washed three time in TBST and then incubated in secondary antibody (1:2500 dilution anti-rabbit HRP) in 5 % skim milk in TBST for 1h. After three TBST washes, membranes were coated in Immobilon Crescendo Western HRP Substrate (Sigma Cat#WBLUR0100) for 1 minute and imaged using the High Sensitivity setting on BioRad ChemiDoc. For multiple proteins detections, membranes were stripped using the Restore Plus Western Blot Stripping Buffer (ThermoFisher Cat#10016433) for 15 minutes. Protocol was repeated beginning at blocking step for supplemental antibodies.

**RNA Extraction**

*Spheroids:* 3000 spheroids per condition were seeded and embedded in 1,5 mL of Collagen as described above per well of a 6 well plate. Following treatment, media was aspirated and 100 µL collagenase (10,000 units/mL) was added to each well and incubated 37°C while shaking for 10 min. Dissolved collagen was collected and remaining cells were scrapped in PBS and then added

to each tube. Samples were centrifuged 500 x g, 5 min and the supernatants were aspirated. Each well was washed with PBS, and wash was added to Eppendorf tube and used to resuspend each pellet. Samples were centrifuged again 500 x g, 5 min and pellets RNA was extracted with Quick-RNA Miniprep Kit (Zymo Cat#R1054) following manufacturer instructions. Final RNA samples were eluted with 20 µL DNase/RNase-Free water and quantified with NanoDrop.

*Monolayer*: Cells were washed with PBS and RNA extraction extraction was performed using TRIzol™ Reagent (Thermofisher) following manufacturer instructions. Final RNA samples were resuspended with 20 µL DNase/RNase-Free water and quantified with NanoDrop.

**Bulk RNA sequencing**

Bulk RNA sequencing was performed by BGI. For each sample, ≥ 500 ng of total RNA was submitted for library preparation. RNA integrity and quality control were assessed by the service provider prior library construction, and all samples passed quality thresholds. Sequencing libraries were generated according to standard protocols and sequenced on the DNBseq™ platform (BGI) using paired-end 100 bp reads (PE100). Approximately 20 million read pairs were generated per sample, corresponding to ~ 4.0 GB of clean data per sample. Raw sequencing reads were processed and analysed using the Dr. Tom online analysis platform provided by BGI. Three independent biological replicates were included per experimental condition.

**Reverse Transcription and qPCR**

Total RNA was reversed transcribed using dNTPs (Promega Cat#U1515), random primers (Promega Cat#C1181) and SuperScript™ III (Invitrogen Cat#11766050). qPCR was performed in an optical 384 well plate (ThermoFisher Cat#4483285) using Fast SYBR Green Master Mix (Invitrogen Cat#4385617). RT-qPCR run and analysis was performed on a QuantStudio 5 instrument (Applied Biosystems). Data was normalised to 36B4 housekeeping gene expression. Fold change in gene expression was determined by the $2^{-\Delta\Delta CT}$ method. Primer sequences are listed in Supplementary Table 5.

**siRNA Transfection**

siRNA transfection was performed using Lipofectamine™ RNAiMAX Transfection Reagent (Thermo Fisher) according to manufacturer's instructions. siRNAs targeting GSK3β, MAP4K4 and a Silencer™ Negative Control were all purchased from Thermo Fisher and used at a final concentration of 10 nM. For the sprouting assay in siRNA-transfected LECS, cells were dissociated

48 hours post-transfection, and spheroids were generated using the hanging drop technique as described previously (1). Briefly, cells were resuspended in a 20% (v/v) Methocel solution in EGM-I. 25μL drops containing 400 cells each were placed on the inside lid of a Petri dish and incubated upside-down in a humidified incubator at 37°C with 5% $CO_2$. Newly formed spheroids were then collected and embedded in Type I Collagen as described above.

**Photoaffinity Labelling**

Confluent LEC were fed overnight with EGM-I. Then, cells were treated with labelling probes for 2 hours at 37°C, 5% $CO_2$ in EGM-I. Cells were then washed twice in PBS and UV irradiated on ice for 10 minutes without lids. Cells were scrapped in presence of lysis buffer (1% NP-40, 0.1% SDS in PBS supplemented with 1:20 dilution protease inhibitor and 1:100 dilution phosphatase inhibitor cocktail) and lysed at 4°C for 30 minutes. Following lysis, samples were centrifuged 13,000 x g for 30 minutes at 4°C. The resulting supernatants were used to measure protein concentrations by BCA assay. 300 μg of proteins were used for click chemistry. A click chemistry master mix was pre-mixed in the following order: 3 μL 50 mM copper (II) sulfate, 3 μL 50mM TCEP, 1.5 μL 10mM TBTA, 1.5 μL 10mM biotin azide (volumes per 100 μL protein solution). 9 μL mastermix was added per 100 μL of protein solution to each sample, vortexed, and samples were put on shaker overnight at 4°C. The following day, 3 μL 0.5M EDTA were added per 100 μL of protein solution to quench click chemistry and proteins were precipitated using Amicon Ultra Centrifugal Filter (Sigma Cat#UFC501024) following manufacturer instructions. Precipitated proteins were then used for western blot.

**Flow cytometry**

Post-MI hearts were harvested, placed in cold HBSS (Life Technologies), finely minced into small pieces with a blade and digested with collagenase type II (StemCell Technologies) and DNase I solution (New England Biolabs) in HBSS at 37°C for 10 minutes. Supernatant was passed through 40 μm filter into a new tube in presence of FBS (Sigma-Aldrich) to deactivate enzyme activity and keep on ice. Remaining hearts pieces were further digested with a fresh collagenase type II + DNase I solution for another 10 minutes at 37°C. Hearts pieces were further dissociated using a gentleMACS Dissociator (Miltenyi Biotec), passed through a cell strainer and combined with previous cell suspension in HBSS + FBS. Single cell suspension was then spun and the pellet

resuspended with Red Blood Lysis (BioLegend) solution following manufacturer instructions. After centrifugation, Red Blood Lysis solution was replaced by 2% FBS-PBS and cells were ready to proceed to staining protocol.

Conjugated antibodies (Supplementary Table 5) against CD45 (pan-leukocyte marker), CD11b ($CD45^+CD11b^+$, myeloid cells), Ly6G ($CD45^+CD11b^+Ly6G^+$, neutrophils), F4/80 ($CD45^+CD11b^+Ly6G^-F4/80^+$, macrophages) and CD11c ($CD45^+CD11b^+Ly6G^-F4/80^-CD11c^+$, dendritic cells) were used in 2%FBS-PBS for 30 minutes at room temperatures. Cells were washed twice and 7-aminoactinomycin D high fluorescence dye (7AAD) was added 5 minutes prior to cell analyses to determine cell viability and exclude dead cells. Single staining, fluorescence minus one controls and compensation calculation were performed. The Flow cytometric analyses were performed using a BD FACSAriaIII flow cytometer (BD Biosciences) and FlowJo software.

**Vibratome**

4-, 7- and 21-dpi hearts were first cut in half (long axis) and both halves embedded in 6% Low Melting Point Agarose (Thermofisher) in PBS. Block of Agarose containing halved-heart was then mounted on a vibratome (Leica) in PBS + 0.1% Tween-20 and 200 µM slices were sectioned at the speed of 0.65 mm/s and a frequency of 70 Hz. Slices were collected and kept in PBS-Tween 0.1% until staining.

Sections were blocked and permeabilised in 2x blocking buffer (PBS + 0.5% Triton-X100, 0.5% Tween-20, 1% BSA and 3% donkey serum) for 1 hour at room temperature. This buffer was replaced by 1x blocking buffer (diluted in PBS) in presence of antibodies (1/100 - Supplementary table 6) for 4 days at 4°C. After 3 x 20 minutes washes with PBS + 0.3% Triton at room temperature, secondary antibodies (1/200 - Supplementary table 6) in 1x blocking buffer were added for 48h, protected from the light, at 4°C. Sections were then washed 3 x 20 minutes with PBS + 0.3% Triton and 2 x 10 minutes PBS + 0.1% Tween-20, transferred on µ-Dish with glass bottom (Ibidi) and cleared overnight with Ce3D solution (2). Sections were then imaged on a Zeiss 980 Confocal microscope with an Airyscan detector.

For quantification, image analysis was performed using ImageJ/FIJI software. The percentage of lymphatic vessel coverage was determined by the percentage of area covered by $Lyve1^+$ across three distinct regions of interest (ROIs): the whole heart (excluding the atria), the left ventricle and

the suture area. Thresholding was applied uniformly to isolate specific Lyve1⁺ signals and a particle size threshold was applied to exclude small objects, such as single macrophages. The remaining positive area was then measured and expressed as a percentage of the total area for each ROI. Global vascular density was measured in the same way using CD31⁺ staining with the ImageJ/FIJI software.

**Echocardiography**

Cardiac function was assessed by transthoracic echocardiography using the Vevo F2 imaging system (FUJIFILM VisualSonics). Imaging was performed at baseline (D0) and at 7, 14 and 21 dpi. Anaesthesia was induced with 4% isofluorane in medical oxygen and maintained at 1,5% on a nose-cone. Mice were placed supine on a heated platform to maintain body temperature at 37°C. Heart rate was continuously monitored and maintained within the physiological range during image acquisition. Two-dimensional B-mode images were acquired in both long-axis and short-axis views at the level of the papillary muscles. At least two cardiac cycles were recorded for each measurement. Data analysis and cardiac function parameters, including ejection fraction (EF), fractional shortening (FS), stroke volume (SV) and cardiac output (CO), were calculated using the Simpson method with the Vevo Lab analysis software (FUJIFILM VisualSonics) according to manufacturer guidelines.

1. F. Tetzlaff, A. Fischer, Human Endothelial Cell Spheroid-based Sprouting Angiogenesis Assay in Collagen. *Bio Protoc* **8**, e2995 (2018).
2. W. Li, R. N. Germain, M. Y. Gerner, High-dimensional cell-level analysis of tissues with Ce3D multiplex volume imaging. *Nat Protoc* **14**, 1708–1733 (2019).

**Chemistry Methods**

*General Synthetic Procedures:*

All compounds and reagents were purchased from Merck Life Sciences, Fluorochem, Alfa Aesar, Enamine, Thermo Fisher or Aurum Pharmatech unless otherwise noted and were used without further purification. Thin later chromatography with Merck Supelco Silica Gel 60 $F_{254}$ Aluminum sheets (Cat #105570), using 254 nM UV light as the visualizing agent, was used to monitor reaction progress. Flash column chromatography was carried out using a Biotage Selekt with Biotage Sfar Silica HC D columns ($SiO_2$, 20 μM). Final products were purified by reverse-phase chromatography on a Biotage Isolera with a Biotage Sfar 18 Duo column (30 μM). $^1$H and $^{13}$C NMR spectra were recorded on a Bruker AVIIIHD 400 Nanobay or a Bruker NEO 600 spectrometer. Chemical shifts are reported in δ units (ppm) relative to residual solvent peak. Characterization data are reported in the following order: chemical shift, multiplicity (s = singlet, d = doublet, t = triplet, q = quartet, m = multiplet, br = broad), coupling constants, number of protons. Each compounds mass was confirmed on a Waters RDa bench-top TOF with an Aquity LC system in direct infusion mode.

*Compound Synthesis and Characterization:*

**Synthesis of Kenpaullone**

*Methyl 2-(4-methoxy-4-oxobutanamido)benzoate, S1*

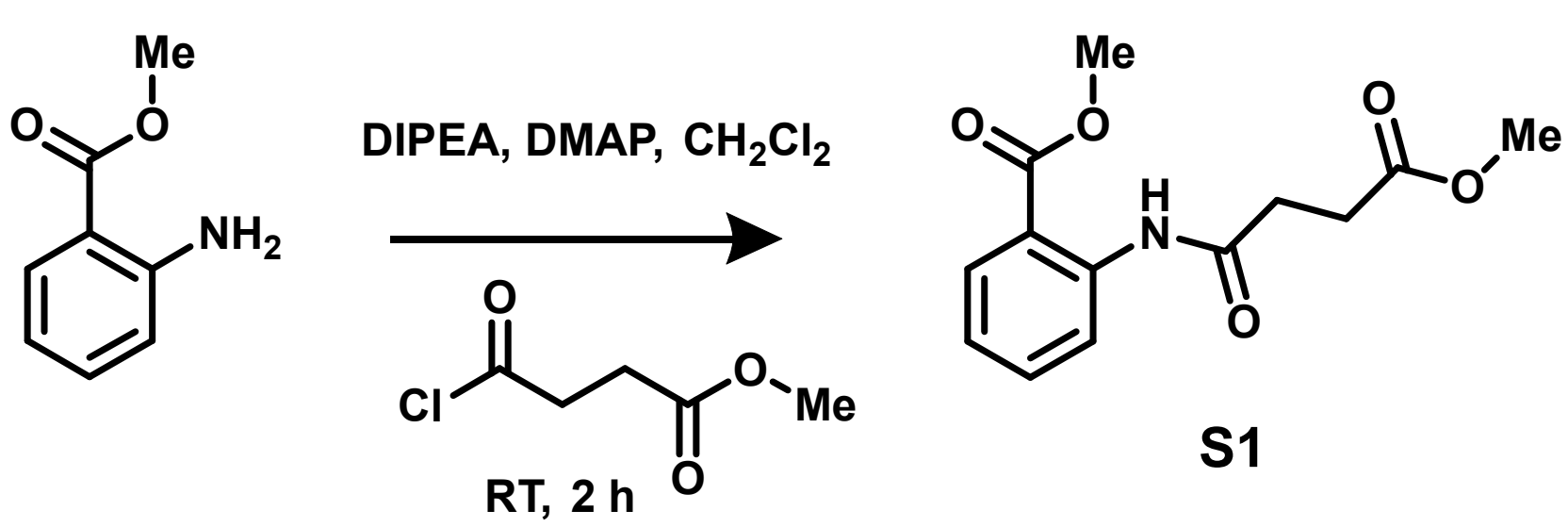


S1 was prepared according to Huang et al.[1] To a mixture of methyl-2-aminobenzoate (5.00 g, 33 mmol, 1 eq) in $CH_2Cl_2$ (100 mL) was added DIPEA (6.0 mL, 34.7 mmol, 1.05 eq) and catalytic DMAP followed by the dropwise addition of methyl succinyl chloride (5.47 g, 36.3 mmol, 1.1 eq). The reaction mixture was stirred at RT for 2 h under $N_2$. Upon reaction completion, 40 mL of $H_2O$ was added to reaction and stirred for 30 min. Product was extracted with $CH_2Cl_2$ (3 X 50 mL),

organic layers were combined, washed with brine (1 X 100 mL), and concentrated in vacuo. The resulting concentrate was purified on Biotage Selekt (0 – 40% EtOAc in Pentane) to afford S1 as a light yellow oil (8.578 g, 32.34 mmol, 98%). $^{1}$H NMR (400 MHz, $CDCl_3$) δ 11.10 (s, 1H), 8.64 (dd, J = 8.5, 1.2 Hz, 1H), 7.97 (dd, J = 8.0, 1.7 Hz, 1H), 7.47 (td, J = 8.8, 7.3, 1.7 Hz, 1H), 7.02 (td, J = 8.3, 7.3, 1.2 Hz, 1H), 3.80 (s, 3H), 3.66 (s, 3H), 2.79 – 2.65 (m, 4H). $^{13}$C NMR (101 MHz, $CDCl_3$) δ 173.0, 170.2, 168.7, 141.4, 134.61, 130.8, 122.4, 120.3, 114.8, 52.3, 51.8, 32.7, 28.9. HRMS: calculated for $C_{13}H_{15}NO_5$ [M + H]$^+$ 266.10, found 266.10. All data were in accordance with the literature.[1]

*Methyl 5-hydroxy-2-oxo-2,3-dihydro-1H-benzo[b]azepine-4-carboxylate, S2*

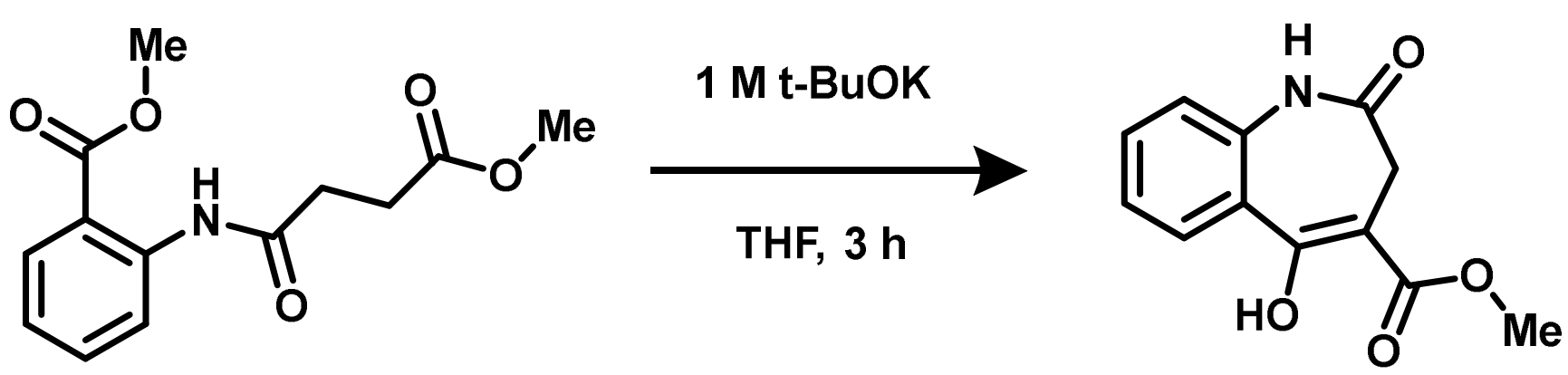


**S1** **S2**

S2 was prepared according to Duffey et al.[2] 1 M t-BuOK (22.5 mL, 22.5 mmol, 3 eq) was added dropwise at 10°C over 30 min to a stirred solution of S1 (2.0 g, 7.5 mmol, 1 eq) in dry THF (43 mL) under $N_2$. After 3 h of stirring at RT, the reaction was quenched with $H_2O$ (15 mL) and 1 M HCl was added until the mixture reached pH ~ 4. It was then stirred for an additional 40 min. The product was extracted with EtOAc (3 X 40 mL), organic layers were combined, washed with brine (1 X 75 mL) and concentrated in vacuo. The resulting concentrate was purified on Biotage Selekt (0 – 50% EtOAc in Pentane) to afford S2 as a white solid (1.41 g, 6.03 mmol, 80%). $^{1}$H NMR (400 MHz, DMSO-$d_6$) δ 12.45 (s, 1H), 10.34 (s, 1H), 7.80 (dd, J = 8.0, 1.6 Hz, 1H), 7.54 (td, J = 8.1, 7.2, 1.6 Hz, 1H), 7.26 (td, J = 8.1, 7.3, 1.2 Hz, 1H), 7.18 (dd, J = 8.3, 1.2 Hz, 1H), 3.84 (s, 3H), 2.93 (s, 2H). $^{13}$C NMR (101 MHz, DMSO-$d_6$) δ 172.0, 170.8, 166.2, 137.9, 131.9, 128.2, 124.7, 123.5, 121.6, 95.8, 52.5, 30.7. HRMS: calculated for $C_{12}H_{11}NO_4$ [M + H]$^+$ 234.07, found 234.07. All data were in accordance with the literature.[2]

*3,4-dihydro-1H-benzo[b]azepine-2,5-dione, S3*

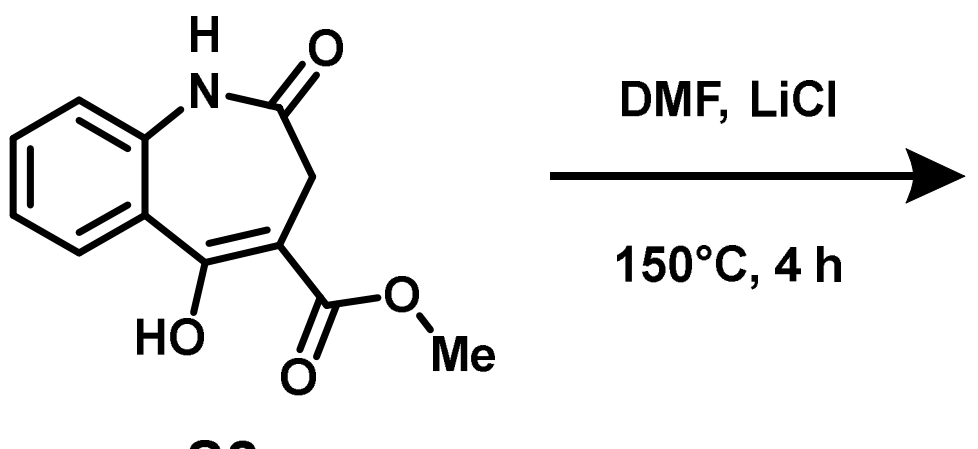


S2 (1 g, 4.29 mmol, 1 eq) and LiCl (36 mg, 0.86 mmol, 0.2 eq) were stirred under $N_2$ in dry DMF (15 mL) at 150°C for 4 h. The reaction was cooled to RT and $H_2O$ (15 mL) was added. Product was extracted with EtOAc (3 X 20 mL), organic layers were combined, washed with brine (3 X 15 mL), and concentrated in vacuo. The resulting concentrate was purified on Biotage Selekt (0 – 50% EtOAc in Pentane) to afford S3 as a white solid (532 mg, 3.04 mmol, 71%). $^1$H NMR (400 MHz, DMSO-$d_6$) $\delta$ 10.08 (s, 1H), 7.85 – 7.78 (m, 1H), 7.59 – 7.49 (m, 1H), 7.17 (t, J = 7.6 Hz, 2H), 2.95 – 2.86 (m, 2H), 2.70 – 2.62 (m, 2H). $^{13}$C NMR (101 MHz, DMSO-$d_6$) $\delta$ 198.7, 173.5, 139.3, 134.1, 130.3, 126.7, 123.4, 121.7, 38.3, 29.3. HRMS: calculated for $C_{10}H_9NO_2$ $[M + H]^+$ 176.06, found 175.956. All data were in accordance with the literature.[3]

*9-bromo-7,12-dihydrobenzo[2,3]azepino[4,5-b]indol-6(5H)-one, kenpaullone*

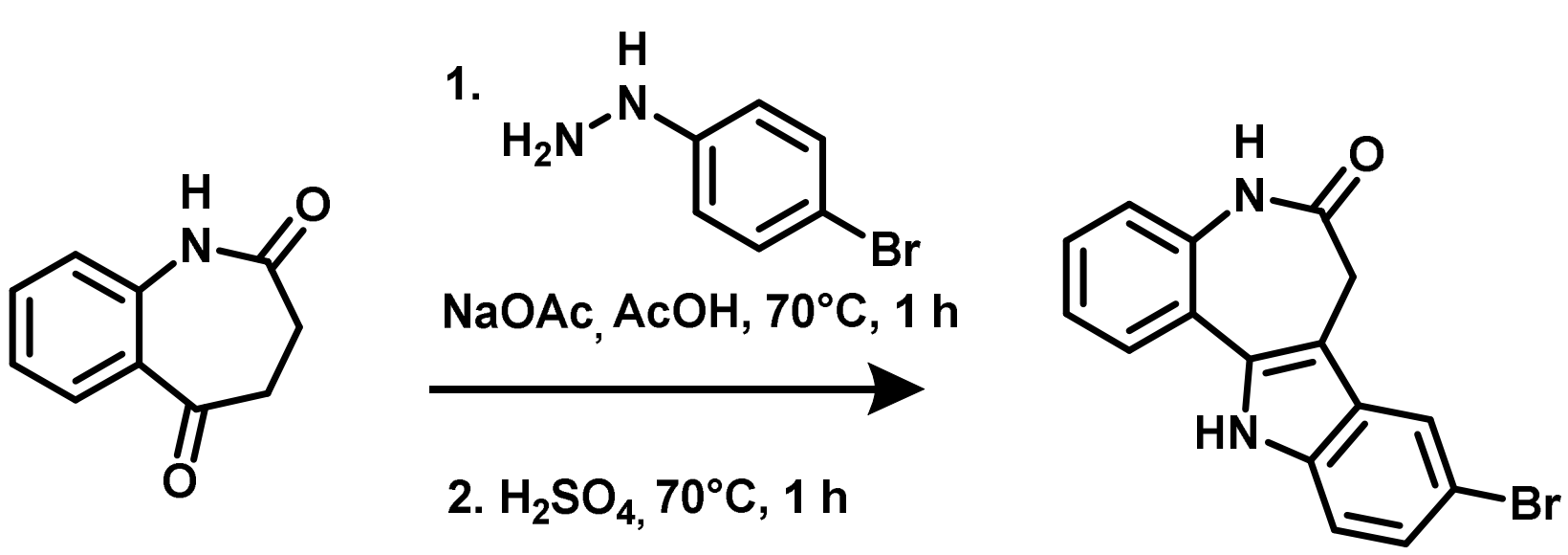


Kenpaullone was synthesized from S3 according to Lee et al.[4] S3 (532 mg, 3.04 mmol, 1 eq), 4-bromophenylhydrazine (1.02 g, 4.56 mmol, 1.5 eq), and sodium acetate (374 mg, 4.56 mmol, 1.5 eq) were stirred in glacial acetic acid (20 mL) at 70°C for 1 h. $H_2SO_4$ (0.20 mL) was then added and reaction was stirred for 1 h at 70°C. The reaction was then cooled to RT and poured into a 5% aqueous solution of sodium acetate to precipitate product. The solids were filtered, washed with

water, and further purified on Biotage Isolera (0 – 100% ACN + 0.1% FA in $H_2O$ + 0.1% FA) to afford Kenpaullone as a yellow powder (421 mg, 1.28 mmol, 42%). $^1$H NMR (600 MHz, DMSO-d$_6$) δ 11.80 (s, 1H), 10.10 (s, 1H), 7.91 (d, J = 1.9 Hz, 1H), 7.74 (dd, J = 7.8, 1.5 Hz, 1H), 7.46 – 7.35 (m, 2H), 7.33 – 7.24 (m, 3H), 3.52 (s, 2H). $^{13}$C NMR (151 MHz, DMSO-d$_6$) δ 171.4, 136.0, 135.7, 134.0, 128.4, 128.3, 126.7, 124.5, 123.7, 122.3, 122.3, 120.4, 113.4, 111.7, 107.2, 31.4. HRMS: calculated for $C_{16}H_{11}BrN_2O$ $[M + H]^+$ 327.01, found 327.07. All data were in accordance with the literature.[4]

**Synthesis of KP-AN-A**

*7,12-dihydrobenzo[2,3]azepino[4,5-b]indol-6(5H)-one, KP-AN-A*

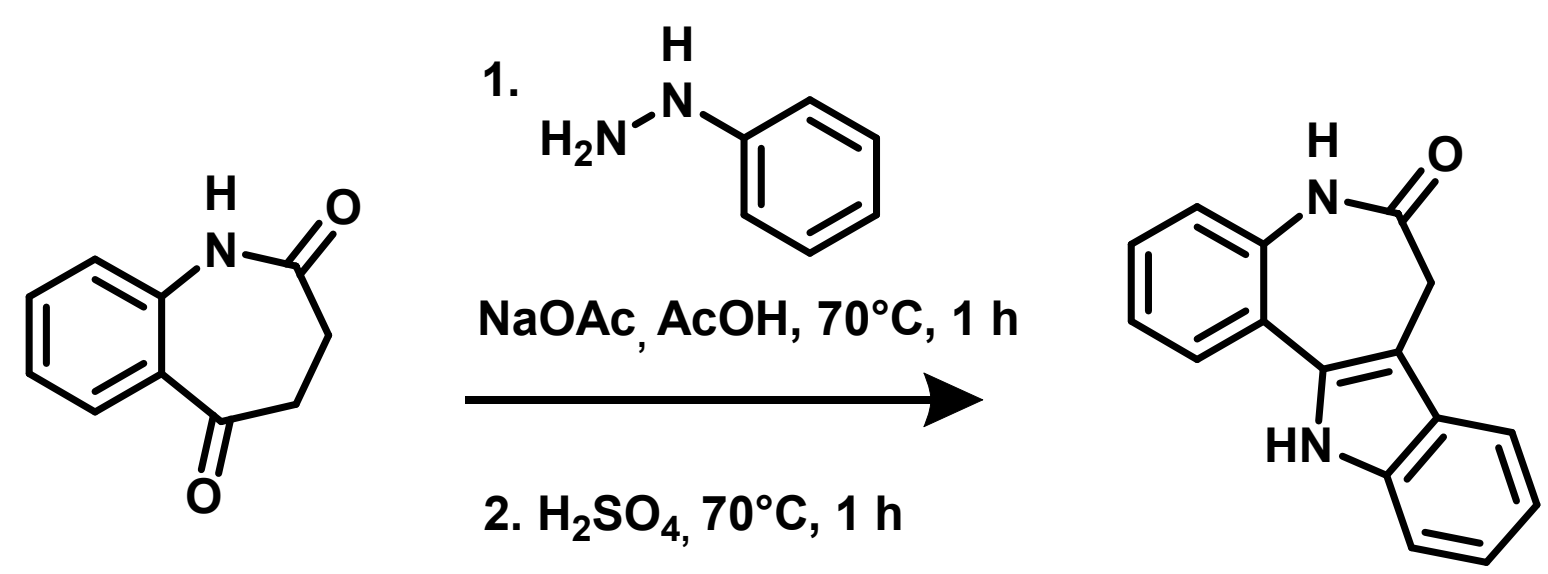


S3 KP-An-A

KP-An-A was synthesized from S3 according to Lee et al.[4] S3 (200 mg, 1.14 mmol, 1 eq), phenylhydrazine (168.8 μL, 1.72 mmol, 1.5 eq), and sodium acetate (141 mg, 1.71 mmol, 1.5 eq) were stirred in glacial acetic acid (20 mL) at 70°C for 1 h. $H_2SO_4$ (0.20 mL) was then added and reaction was stirred for 1 h at 70°C. The reaction was then cooled to RT and poured into a 5% aqueous solution of sodium acetate to precipitate product. The solids were filtered, washed with water, and further purified on Biotage Isolera (0 – 100% ACN + 0.1% FA in $H_2O$ + 0.1% FA) to afford KP-An-A as a yellow solid (125 mg, 0.50 mmol, 44%). $^1$H NMR (600 MHz, DMSO-d$_6$) δ 11.57 (s, 1H), 10.07 (s, 1H), 7.74 (dd, J = 7.7, 1.5 Hz, 1H), 7.66 (dd, J = 7.7, 1.0 Hz, 1H), 7.44 (dt, J = 8.1, 0.9 Hz, 1H), 7.37 (ddd, J = 8.0, 7.3, 1.5 Hz, 1H), 7.30 – 7.23 (m, 2H), 7.17 (ddd, J = 8.1, 7.0, 1.1 Hz, 1H), 7.07 (ddd, J = 8.0, 7.0, 1.0 Hz, 1H), 3.50 (s, 2H). $^{13}$C NMR (151 MHz, DMSO-d$_6$) δ 171.6, 137.4, 135.4, 132.5, 127.9, 126.9, 126.6, 123.6, 122.8, 122.2, 122.1, 119.1, 117.9, 111.4,

107.6, 31.6. HRMS: calculated for $C_{16}H_{12}N_2O$ [M + H]$^+$ 249.09, found 249.18. All data were in accordance with the literature.[4]

**Synthesis of KP-An-B**

*5,6,7,12-tetrahydrobenzo[6,7]cyclohepta[1,2-b]indole, KP-An-B*

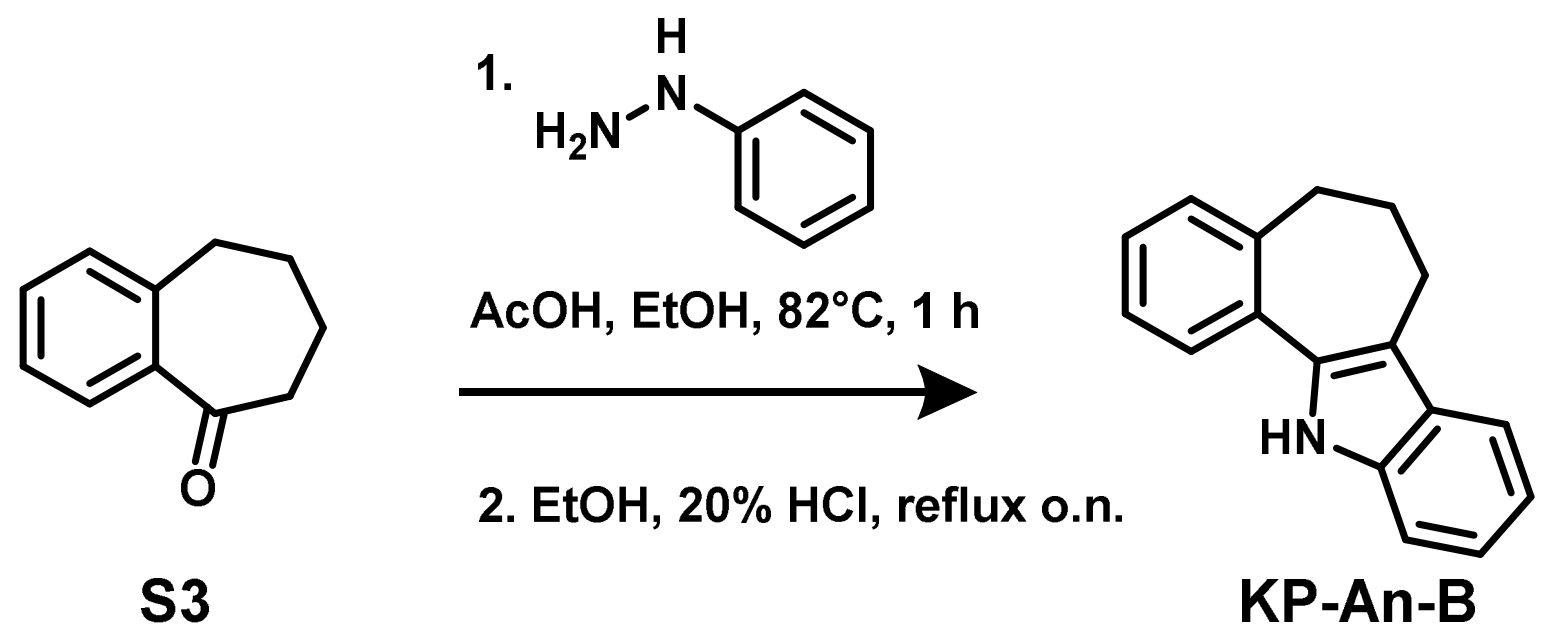


KP-An-B was prepared according to Yadav et al.[5] Phenylhyrdrazine (108 mg, 1 mmol, 1 eq), benzosuberone (169 mg, 1 mmol, 1 eq), and glacial acetic acid (50 μL) were refluxed in EtOH (8 mL) for 6 h. Reaction was cooled on ice, filtered, and crude hydrazone was resuspended in EtOH (4 mL) and 20% aq. HCl (3 mL), and refluxed for 12 h. The solution was cooler on ice and neutralized with 5% aq. NaOH. The resulting precipitate was loaded directly onto the Biotage Isolera (0 – 100% ACN + 0.1% FA in $H_2O$ + 0.1% FA) for purification to afford KP-An-B as a white solid (63 mg, 0.27 mmol, 27%). $^1$H NMR (400 MHz, $CDCl_3$) δ 8.09 (s, 1H), 7.60 (ddd, 2H), 7.29 (m, 6H), 3.15 (t, 2H), 2.95 (t, 2H). All data were in accordance with the literature.[5]

**Synthesis of KP-DA**

*Methyl 2-amino-5-methoxybenzoate, S4*

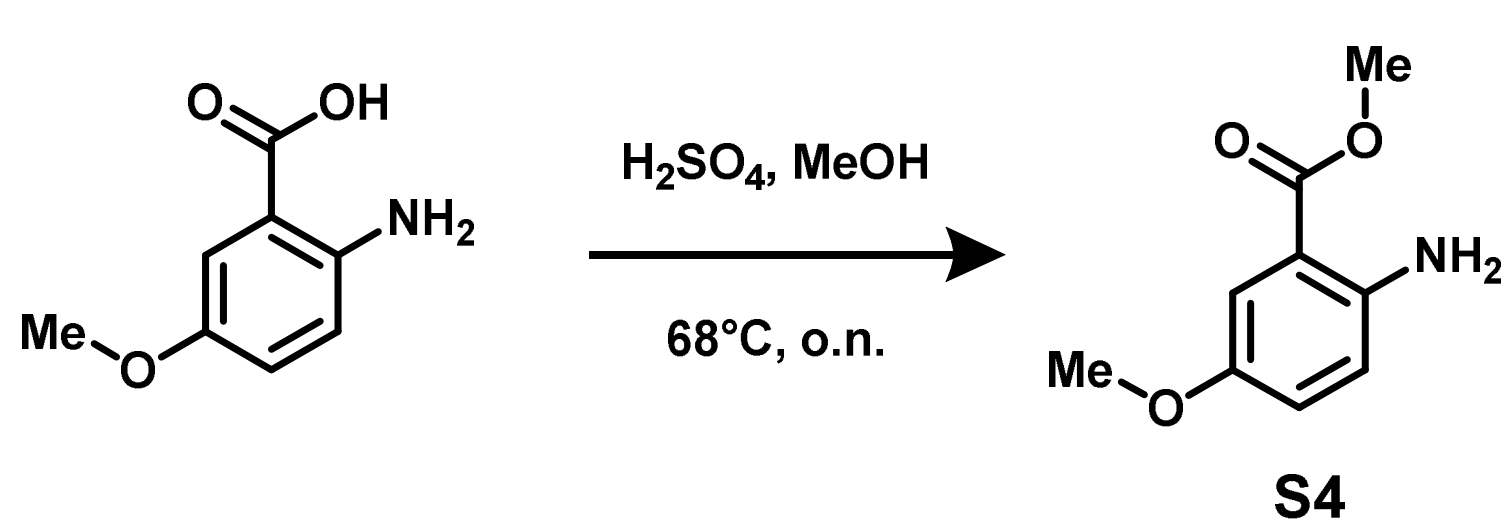

S4 was prepared according to Dang et al.[6] $H_2SO_4$ (6.8 mL) was added dropwise over 3 min to a stirred suspension of 5-methoxyanthranilic acid (3.80 g, 22.73 mmol, 1 eq) in dry MeOH (67 mL). The mixture was refluxed overnight under $N_2$. The MeOH was evaporated in vacuo and the resulting residue was diluted with saturated $NaHCO_3$ (250 mL) and extracted with EtOAc (3 X 75 mL), organic layers were combined, washed with brine (1 X 100 mL), and concentrated in vacuo. The resulting concentrate was purified on Biotage Selekt (0 – 35% EtOAc in Pentane) to afford S4 as a yellow oil (3.54 g, 19.55 mmol, 86%). $^1$H NMR (400 MHz, $CDCl_3$) $\delta$ 7.34 (d, J = 3.0 Hz, 1H), 6.94 (dd, J = 8.9, 3.0 Hz, 1H), 6.62 (dd, J = 8.9, 0.4 Hz, 1H), 5.32 (br, 2H), 3.87 (s, 3H), 3.75 (s, 3H). $^{13}$C NMR (101 MHz, $CDCl_3$) $\delta$ 168.4, 150.7, 145.2, 123.4, 118.4, 113.3, 110.8, 55.9, 51.7. HRMS: calculated for $C_9H_{11}NO_3$ $[M + H]^+$ 182.07, found 182.03. All data were in accordance with the literature.[6]

*Methyl 5-methoxy-2-(4-methoxy-4-oxobutanamido)benzoate, S5*

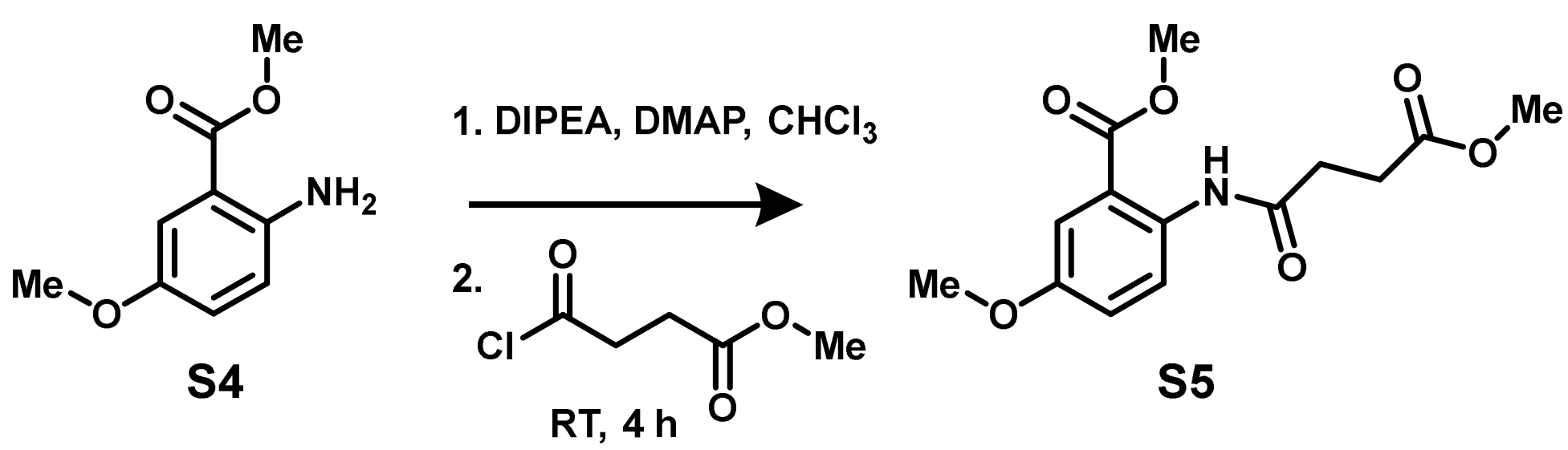


To a mixture of S4 (1.185 g, 6.54 mmol, 1 eq) in $CHCl_3$ (31 mL) was added DIPEA (1.1 mL, 6.5 mmol, 1 eq) and catalytic DMAP followed by the dropwise addition of methyl succinyl chloride (0.88 mL, 7.20 mmol, 1.1 eq) over 10 min. Reaction was stirred at RT for 4 h under $N_2$. Upon reaction completion, $H_2O$ (20 mL) was added to reaction and stirred for an additional 30 min. Product was extracted with $CH_2Cl_2$ (3 X 30 mL), organic layers were combined, washed with brine (1 X 50 mL), and concentrated in vacuo. The resulting concentrate was purified on Biotage Selekt (0 – 100 % EtOAc in Pentane) to afford S5 as a white powder (1.84 g, 6.21 mmol, 95%). $^1$H NMR (400 MHz, $CDCl_3$) $\delta$ 10.86 (s, 1H), 8.60 (d, J = 9.2 Hz, 1H), 7.50 (d, J = 3.1 Hz, 1H), 7.14 – 7.06 (m, 1H), 3.93 (s, 3H), 3.81 (s, 3H), 3.71 (s, 3H), 2.81 – 2.69 (m, 4H). $^{13}$C NMR (101 MHz, $CDCl_3$) $\delta$ 173.2, 169.9,

168.5, 154.6, 135.3, 122.2, 121.1, 116.1, 114.7, 55.8, 52.6, 52.0, 32.8, 29.2. HRMS: calculated for $C_{14}H_{17}NO_6$ $[M + H]^+$ 296.11, found 296.23. All data were in accordance with the literature.[7]

*Methyl 5-hydroxy-7-methoxy-2-oxo-2,3-dihydro-1H-benzo[b]azepine-4-carboxylate, S6*

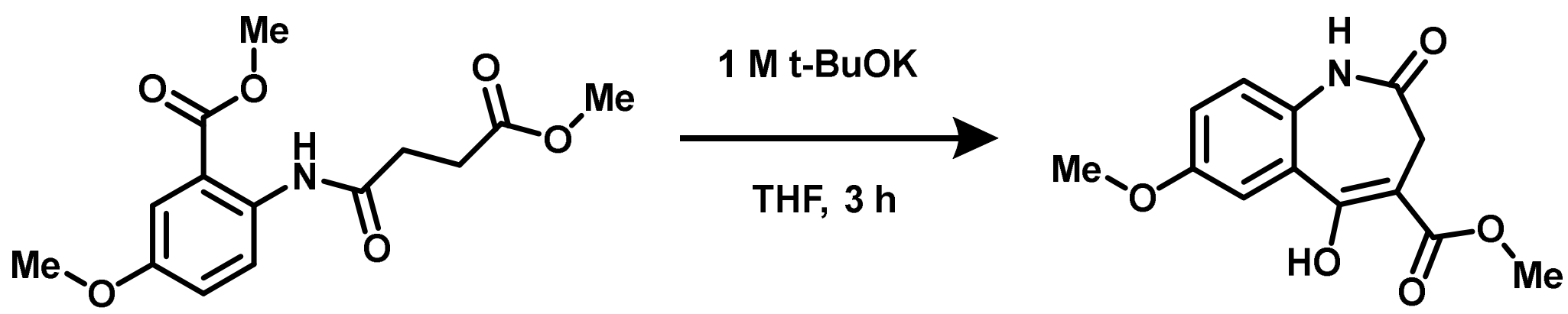


S5 S6

S6 was prepared according the following patent.[7] 1 M t-BuOK in THF (12.48 mL, 6.24 mmol, 2 eq) was added dropwise at 10°C over 30 min to a solution of S5 (1.842 g, 6.24 mmol, 1 eq) in dry THF (17 mL) under $N_2$. After 3 h of stirring at RT, the reaction was quenched with $H_2O$ (15 mL) and 1 M HCl was added until the mixture reached pH ~ 4. Reaction was stirred an additional 40 min. Product was extracted with EtOAc (3 X 30 mL), organic layers were combined, washed with brine (1 X 30 mL), and concentrated in vacuo. The resulting concentrate was purified on Biotage Selekt (0 – 100% EtOAc in Pentane) to afford S6 as a white solid (1.143 g, 4.34 mmol, 70%). $^1H$ NMR (400 MHz, DMSO-$d_6$) $\delta$ 12.46 (s, 1H), 10.14 (s, 1H), 7.24 (d, J = 2.9, 1H), 7.21 – 7.08 (m, 2H), 3.84 (s, 3H), 3.79 (s, 3H), 2.90 (s, 2H). $^{13}C$ NMR (101 MHz, DMSO-$d_6$) $\delta$ 171.8, 170.9, 165.9, 155.1, 131.6, 125.8, 123.3, 119.3, 110.9, 96.2, 55.5, 52.6, 30.7. HRMS: calculated for $C_{13}H_{13}NO_5$ $[M + H]^+$ 264.08, found 264.09. All data were in accordance with the literature.[7]

*7-methoxy-3,4-dihydro-1H-benzo[b]azepine-2,5-dione, S7*

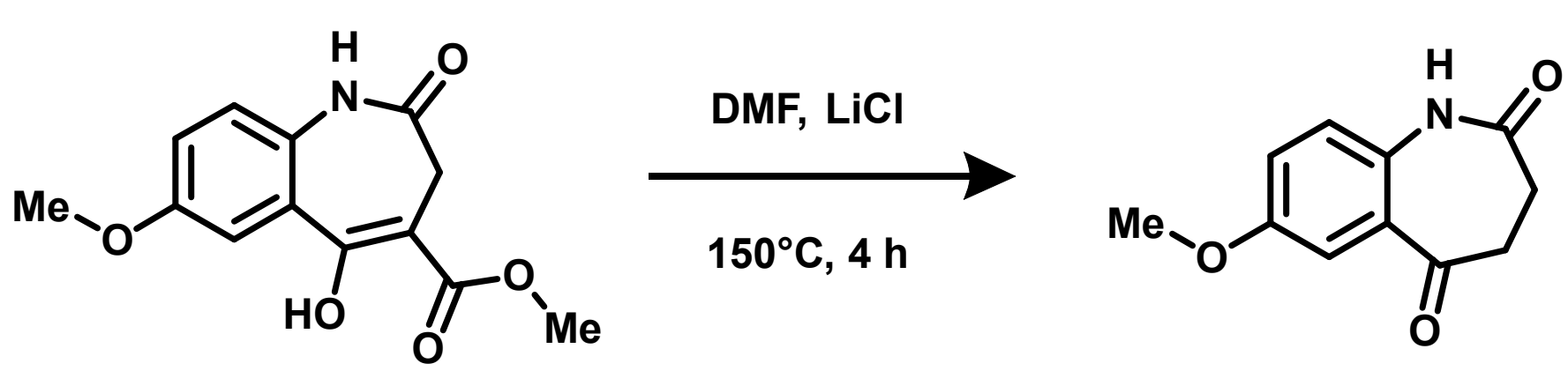


S6 S7

S6 (600 mg, 2.28 mmol, 1 eq) and LiCl (19.32 mg, 0.46 mmol, 0.2 eq) were stirred under $N_2$ in dry DMF (7 mL**)** at 150°C for 4 h. The reaction was cooled to RT and $H_2O$ (15 mL) was added. Product

was extracted with EtOAc (3 X 15 mL) , organic layers were combined, washed with brine (2 X 20 mL), and concentrated in vacuo. The resulting concentrate was purified on Biotage Selekt (0 – 100% EtOAc in Pentane) to afford S7 as a white solid (318 mg, 1.55 mmol, 68%). $^1$H NMR (400 MHz, DMSO-d$_6$) $\delta$ 9.97 (s, 1H), 7.32 (d, J = 3.1 Hz, 1H), 7.24 (dd, J = 8.8, 3.1 Hz, 1H), 7.16 (d, J = 8.8 Hz, 1H), 3.83 (s, 3H), 3.00 – 2.92 (m, 2H), 2.72 – 2.64 (m, 2H). $^{13}$C NMR (101 MHz, DMSO-d$_6$) $\delta$ 198.6, 173.3, 155.0, 132.9, 127.7, 123.5, 121.4, 112.6, 55.4, 38.5, 29.2. HRMS: calculated for $C_{11}H_{11}NO_3$ [M + H]$^+$ 206.07, found 206.108. All data were in accordance with the literature.[8]

*9-bromo-2-methoxy-7,12-dihydrobenzo[2,3]azepino[4,5-b]indol-6(5H)-one, S8*

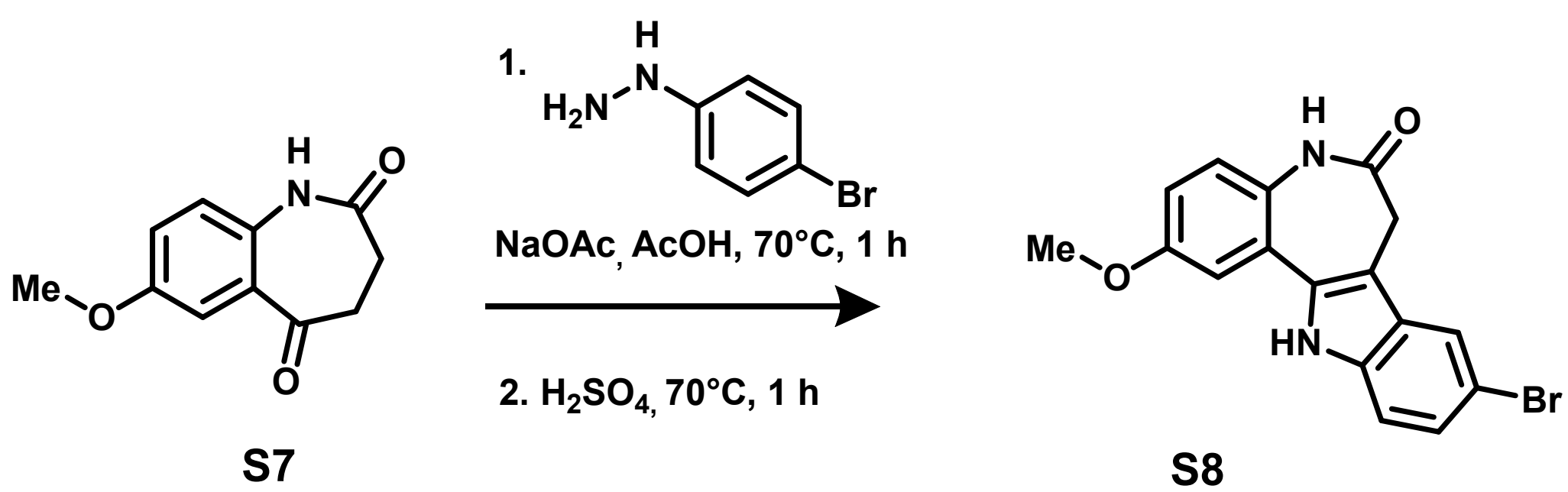


S8 was synthesized from S7 according to Wieking et al.[8] S7 (250 mg, 1.218 mmol, 1 eq), 4-bromophenylhydazine (408.4 mg, 1.83 mmol, 1.5 eq), and sodium acetate (42 mg, 1.8 mmol, 1.5 eq) were stirred in glacial acetic acid (6 mL) at 70°C for 1 h. $H_2SO_4$ (68 $\mu$L) was then added and reaction was stirred for 1 h at 70°C. The reaction was then cooled to RT and poured into a 5% aqueous solution of sodium acetate to precipitate product. The solids were filtered, washed with water, and further purified on Biotage Isolera (0 – 100% ACN + 0.1% FA in $H_2O$ + 0.1% FA) to afford S8 as a yellow solid (146 mg, 0.41 mmol, 34%). $^1$H NMR (600 MHz, DMSO-d$_6$) $\delta$ 11.79 (s, 1H), 9.90 (s, 1H), 7.90 (d, J = 1.9 Hz, 1H), 7.40 (d, J = 8.6 Hz, 1H), 7.29 – 7.26 (m, 2H), 7.18 (d, J = 8.9 Hz, 1H), 7.00 (dd, J = 8.9, 2.9 Hz, 1H), 3.84 (s, 3H), 3.47 (s, 2H). $^{13}$C NMR (151 MHz, DMSO-d$_6$) $\delta$ 171.1, 155.4, 135.9, 134.0, 129.2, 128.3, 124.6, 123.8, 123.3, 120.5, 115.3, 113.4, 111.7, 110.6, 107.4, 55.5, 31.3. HRMS: calculated for $C_{17}H_{13}BrN_2O_2$ [M + H]$^+$ 357.02, found 357.083. All data were in accordance with the literature.[8]

*9-bromo-2-hydroxy-7,12-dihydrobenzo[2,3]azepino[4,5-b]indol-6(5H)-one, S9*

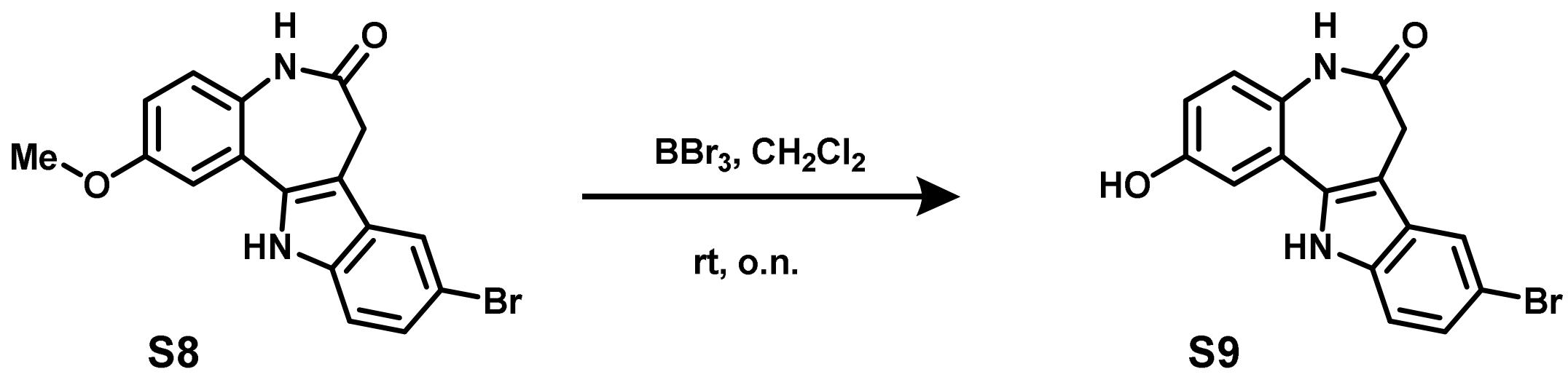


S9 was synthesized from S8 according to Wieking et al.[8] 1 M $BBr_3$ in $CH_2Cl_2$ (1.6 mL, 1.6 mmol, 4 eq) was added to a suspension of S8 (145.9 mg, 0.408 mmol, 1 eq) in dry $CH_2Cl_2$ (4.6 mL) under $N_2$. Reaction was stirred overnight at RT. The following day $H_2O$ (4.6 mL) was added to the reaction and stirred for 1 h. Product was extracted with EtOAc (3 X 15 mL), organic layers were combined, washed with brine (2 X 20 mL), and concentrated in vacuo. The resulting concentrate was purified on Biotage Selekt (0 – 100% EtOAc in Pentane) to afford S9 as a brown solid (70 mg, 0.20 mmol, 50%). $^1$H NMR (400 MHz, DMSO-d$_6$) δ 11.61 (s, 1H), 9.97 (s, 1H), 9.86 (s, 1H), 7.82 (d, J = 2.0 Hz, 1H), 7.53 (d, J = 8.6 Hz, 1H), 7.34 (d, J = 8.5 Hz, 1H), 7.21 (dd, J = 8.6, 1.9 Hz, 1H), 6.75 – 6.65 (m, 2H), 3.45 (s, 2H). $^{13}$C NMR (151 MHz, DMSO-d$_6$) δ 171.2, 153.7, 135.9, 134.2, 128.3, 127.9, 124.4, 123.8, 123.6, 120.4, 116.1, 113.4, 112.4, 111.7, 107.3, 31.3. HRMS: calculated for $C_{16}H_{11}BrN_2O_2$ $[M + H]^+$ 343.00, found 343.023. All data were in accordance with the literature.[8]

*9-bromo-2-(2-(3-(but-3-yn-1-yl)-3H-diazirin-3-yl)ethoxy)-7,12-dihydrobenzo[2,3]azepino[4,5-b]indol-6(5H)-one, KP-DA*

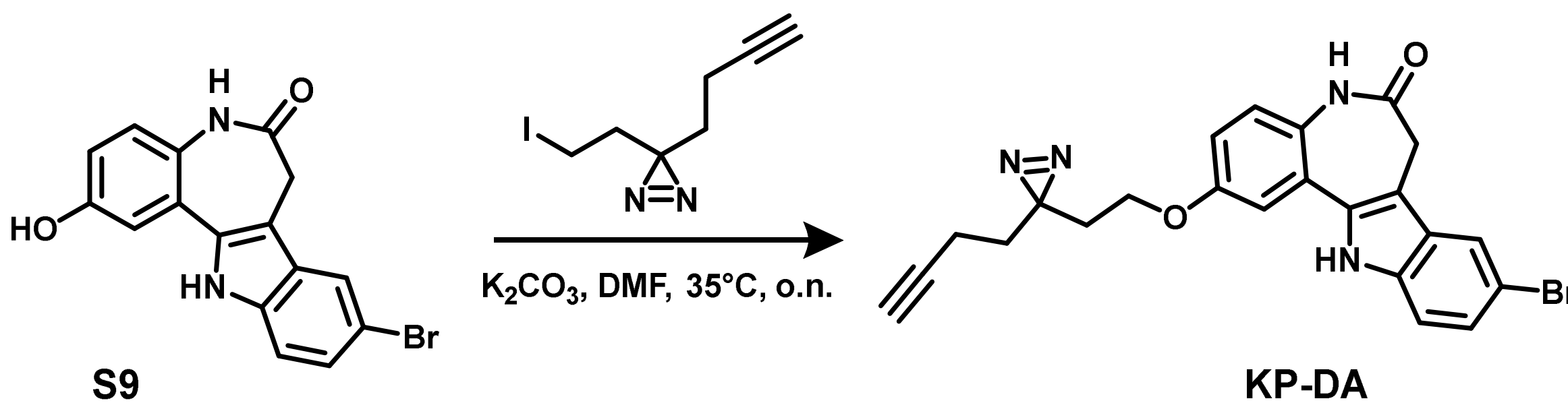


$K_2CO_3$ (30 mg, 0.27 mmol, 2.1 eq) and 3-(but-3-yn-1-yl)-3-(2-iodoethyl)-3*H*-diazirine (25 mg, 0.10 mmol, 1 eq) were added to a suspension of S9 (34.3 mg, 0.10 mmol, 1 eq) in dry DMF (1 mL) under $N_2$. Reaction was stirred overnight at 35°C. The following day the reaction was loaded directly onto

Biotage Isolera (0 – 100% ACN + 0.1% FA in $H_2O$ + 0.1% FA) for purification to afford KP-DA as a yellow solid (2.3 mg, 0.005 mmol, 5%). $^1$H NMR (600 MHz, DMSO-$d_6$) δ 11.80 (s, 1H), 9.93 (s, 1H), 7.90 (d, J = 1.9 Hz, 1H), 7.40 (d, J = 8.5 Hz, 1H), 7.25 (d, J = 2.9 Hz, 1H), 7.17 (d, J = 8.9 Hz, 1H), 6.99 (dd, J = 8.8, 2.8 Hz, 1H), 6.53 (s, 1H), 5.75 (s, 1H), 3.90 (t, J = 6.0 Hz, 2H), 3.48 (s, 2H), 2.06 (td, J = 7.3, 2.6 Hz, 2H), 1.94 (t, J = 6.0 Hz, 2 H), 1.69 (t, J = 7.4 Hz, 2H). $^{13}$C NMR (151 MHz, DMSO-$d_6$) δ 170.4, 154.3, 135.9, 133.9, 129.4, 128.3, 124.7, 123.8, 123.3, 120.5, 115.6, 113.4, 111.7, 111.4, 107.5, 83.2, 71.9, 62.8, 31.8, 31.8, 30.7, 20.8, 12.7. HRMS: calculated for $C_{23}H_{19}BrN_4O_2$ $[M + H]^+$ 463.07, found 463.117. LRMS: found 463.12.

**Synthesis of KP-An-A-DA**

*2-methoxy-7,12-dihydrobenzo[2,3]azepino[4,5-b]indol-6(5H)-one, S10*

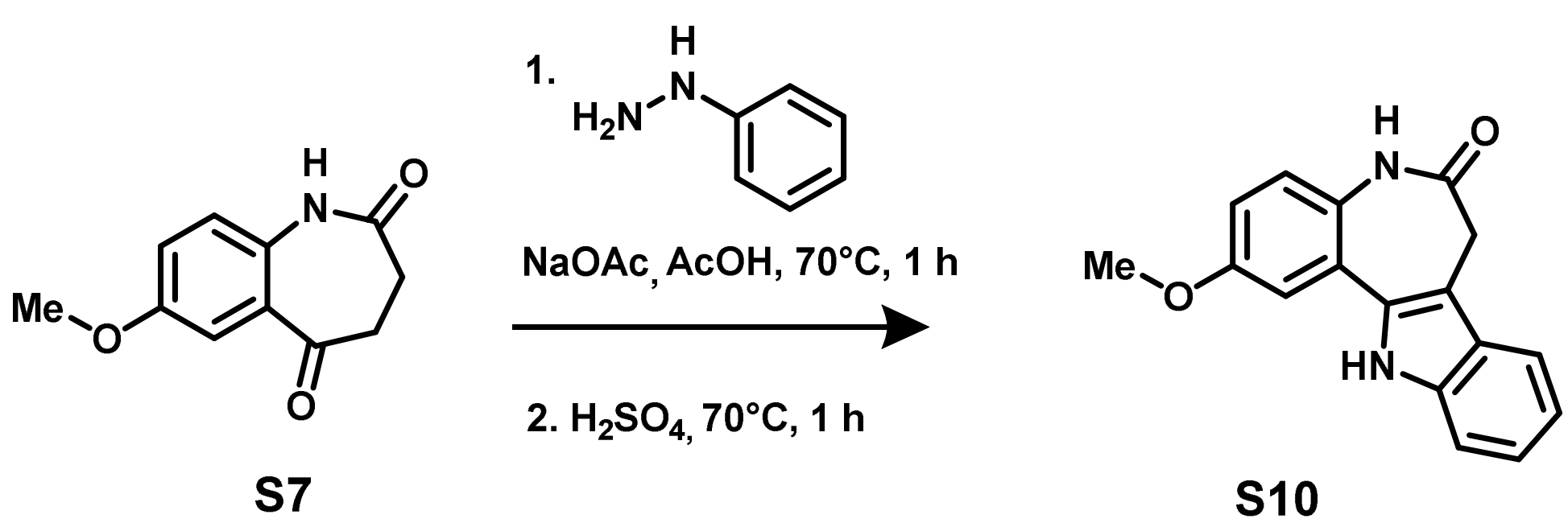


S7 (103 mg, 0.503 mmol, 1 eq), phenylhydrazine (74 μL, 0.75 mmol, 1.5 eq), and sodium acetate (18 mg, 0.75 mmol, 1.5 eq) were stirred in glacial acetic acid (2.5 mL) at 70°C for 1 h. $H_2SO_4$ (28 μL) was then added and reaction was stirred for 1 h at 70°C. The reaction was then cooled to RT and poured into a 5% aqueous solution of sodium acetate to precipitate product. The solids were filtered and washed with water to afford S10 as a yellow solid (98 mg, 0.35 mmol, 70%). $^1$H NMR (600 MHz, DMSO-$d_6$) δ 11.58 (s, 1H), 9.87 (s, 1H), 7.65 (d, J = 7.9 Hz, 1H), 7.44 (dt, J = 8.1, 0.9 Hz, 1H), 7.28 (d, J = 2.9 Hz, 1H), 7.20 – 7.15 (m, 2H), 7.07 (ddd, J = 8.0, 7.0, 1.0 Hz, 1H), 6.97 (dd, J = 8.8, 2.9 Hz, 1H), 3.85 (s, 3H), 3,46 (s, 2H). $^{13}$C NMR (151 MHz, DMSO-$d_6$) δ 171.3, 155.4, 137.3, 132.5, 128.9, 126.5, 123.9, 123.7, 122.2, 119.1, 117.9, 114.8, 111.4, 110.5, 107.8, 55.4, 31.5. HRMS: calculated for $C_{17}H_{14}N_2O_2$ $[M + H]^+$ 279.11, found 279.10.

*2-hydroxy-7,12-dihydrobenzo[2,3]azepino[4,5-b]indol-6(5H)-one, S11*

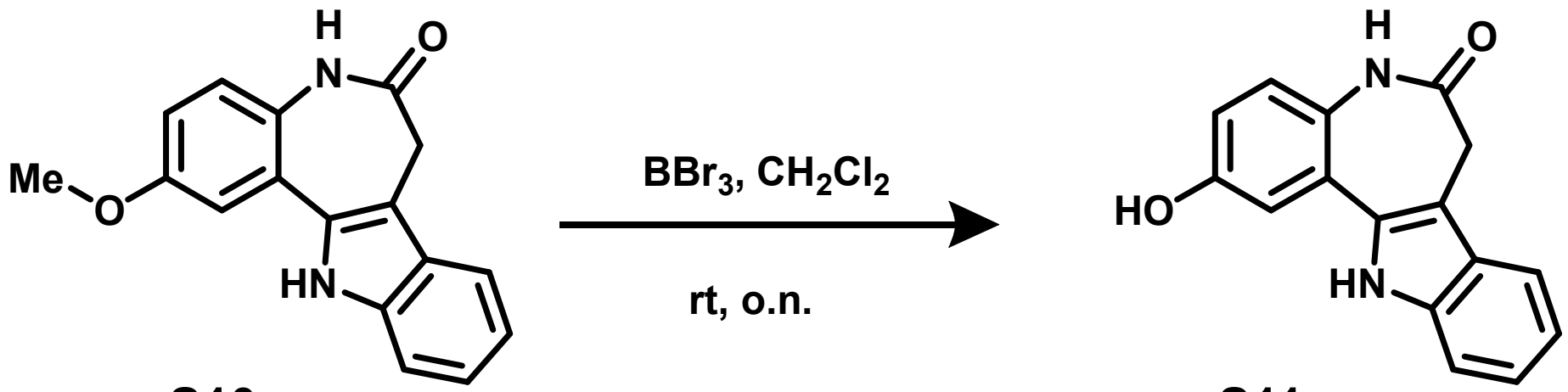

S10 S11

1 M $BBr_3$ in $CH_2Cl_2$ (1.4 mL, 1.4 mmol, 4 eq) was added to a suspension of S10 (98 mg, 0.35 mmol, 1 eq) in dry $CH_2Cl_2$ (3.9 mL) under $N_2$. Reaction was stirred overnight at RT. The following day $H_2O$ (4 mL) was added to the reaction and stirred for 1 h. Product was extracted with EtOAc (3 X 15 mL), organic layers were combined, washed with brine (2 X 20 mL), and concentrated in vacuo. The resulting concentrate was purified on Biotage Selekt (0 – 100% EtOAc in Pentane) to afford S11 as a brown solid (51 mg, 0.19 mmol, 55%). $^1$H NMR (400 MHz, DMSO-$d_6$) $\delta$ 11.38 (s, 1H), 9.95 (s, 1H), 9.78 (s, 1H), 7.58 (d, J = 7.9 Hz, 1H), 7.54, (d, J = 8.5 Hz, 1H), 7.38 (dt, J = 8.1, 1.0 Hz, 1H), 7.11 (ddd, J = 8.2, 7.0, 1.2 Hz, 1H), 7.03 (ddd, J = 8.0, 7.0, 1.1 Hz, 1H), 6.74 – 6.65 (m, 2H), 3.45 (s, 2H). $^{13}$C NMR (151 MHz, DMSO-$d_6$) $\delta$ 171.3, 153.5, 137.3, 132.6, 127.7, 126.5, 124.1, 123.7, 122.0, 119.0, 117.9, 115.6, 112.3, 111.5, 107.7, 31.5. HRMS: calculated for $C_{16}H_{12}N_2O_2$ $[M + H]^+$ 265.09, found 265.16.

*2-(2-(3-(but-3-yn-1-yl)-3H-diazirin-3-yl)ethoxy)-7,12-dihydrobenzo[2,3]azepino[4,5-b]indol-6(5H)-one, KP-An-A-DA*

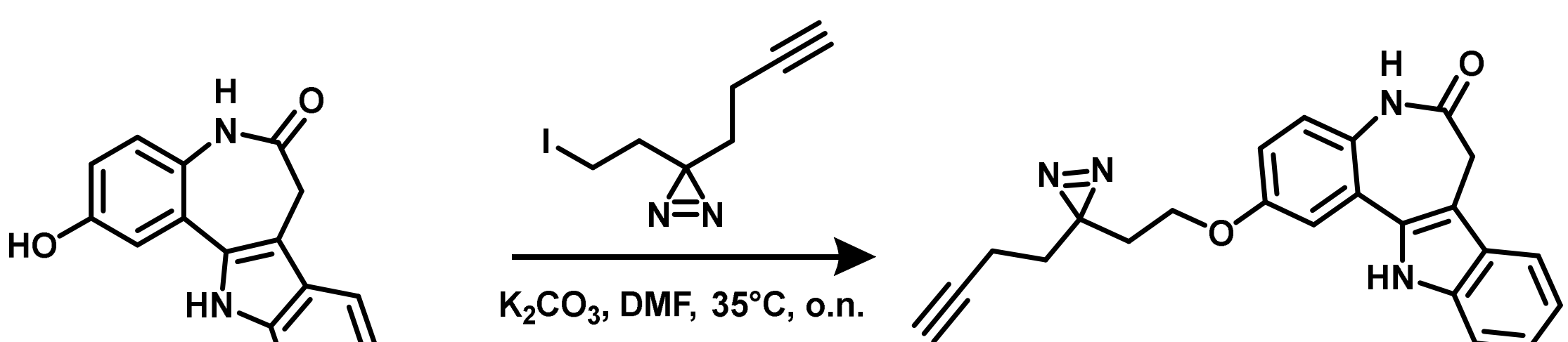

S11 KP-An-A-DA

$K_2CO_3$ (56 mg, 0.41 mmol, 2.1 eq) and 3-(but-3-yn-1-yl)-3-(2-iodoethyl)-3*H*-diazirine (48 mg, 0.20 mmol, 1 eq) were added to a suspension of S11 (51.4 mg, 0.195 mmol, 1 eq) in dry DMF (1 mL) under $N_2$. Reaction was stirred overnight at 35°C. The following day the reaction was loaded directly

onto the Biotage Isolera (0 – 100% ACN + 0.1% FA in $H_2O$ + 0.1% FA) for purification to afford KP-An-A-DA as a yellow solid (3.6 mg, 0.009 mmol, 5%). $^1H$ NMR (600 MHz, DMSO-$d_6$) δ 11.56 (s, 1H), 9.99 (s, 1H), 7.65 (d, J = 8.7 Hz, 1H), 7.61 (d, J = 7.9 Hz, 1H), 7.43 – 7.37 (m, 1H), 7.13 (ddd, J = 8.1, 7.0, 1.2 Hz, 1H), 7.05 (ddd, J = 8.1, 7.0, 1.1 Hz, 1H), 6.90 (dd, J = 8.7, 2.5 Hz, 1H), 6.80 (d, J = 2.5 Hz, 1H), 3.86 (t, J = 6.1 Hz, 2H), 3.48 (s, 2H), 2.85 (t, J = 2.6 Hz, 1H), 2.11 – 2.02 (m, 2H), 1.93 (t, J = 6.0 Hz, 2H), 1.69 (t, J = 7.4 Hz, 2H). $^{13}C$ NMR (151 MHz, DMSO-$d_6$) δ 171.3, 157.9, 137.2, 136.7, 132.6, 128.0, 126.6, 121.7, 118.9, 117.6, 116.1, 111.3, 110.6, 107.5, 105.8, 83.2, 71.8, 62.6, 31.8, 31.7, 31.6, 26.9, 12.7. HRMS: calculated for $C_{23}H_{20}N_4O_2$ $[M + H]^+$ 385.16, found 385.20. LRMS: found 385.03.

**Synthesis of KP-AZ**

*2-(2-azidoethoxy)-9-bromo-7,12-dihydrobenzo[2,3]azepino[4,5-b]indol-6(5H)-one, KP-AZ*

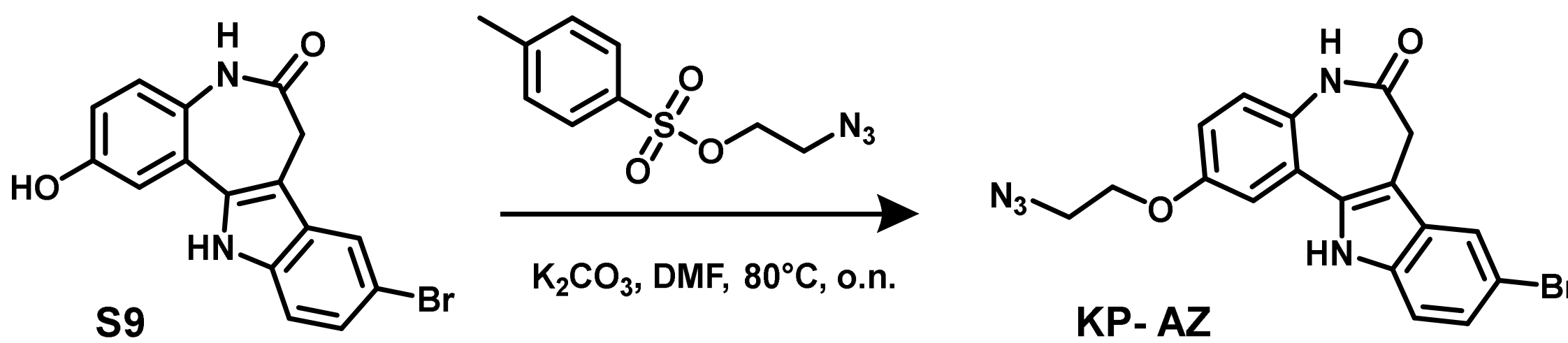


$K_2CO_3$ (12 mg, 0.08 mmol, 1.2 eq) and 0.5 M 1-[(2-azidoethoxy)sulfonyl]-4-methylbenzene (140 μL, 0.07 mmol, 1 eq) were added to a suspension of S9 (24 mg, 0.07 mmol, 1 eq) in dry DMF (500 μL) under $N_2$. Reaction was stirred overnight at 80°C. The following day the reaction was loaded directly onto the Biotage Isolera (0 – 100% ACN + 0.1% FA in $H_2O$ + 0.1% FA) for purification to afford KP-AZ as a yellow solid (1.2 mg, 0.003 mmol, 4%). $^1H$ NMR (600 MHz, DMSO-$d_6$) δ 11.70 (s, 1H), 10.03 (s, 1H), 7.86 (d, J = 1.9 Hz, 1H), 7.67 (d, J = 8.6 Hz, 1H), 7.36 (d, J = 8.6 Hz, 1H), 7.24 (dd, J = 8.5, 1.9 Hz, 1H), 6.95 (dd, J = 8.6, 2.5 Hz, 1H), 6.85 (d, J = 2.6 Hz, 1H), 4.22 (t, J = 4.3 Hz, 2H), 3.70 (t, J = 4.1 Hz, 2H), 3.50 (s, 2H). $^{13}C$ NMR (151 MHz, DMSO-$d_6$) δ 171.2, 158.1, 136.9, 135.8, 134.2, 128.4, 128.3, 124.1, 120.1, 115.8, 113.2, 111.6, 110.6, 107.6, 105.6, 66.9,

39.5, 31.4. HRMS: calculated for $C_{18}H_{14}BrN_5O_2$ $[M + H]^+$ 412.03, found 412.29. LRMS: found 412.02.

**Synthesis of KP-An-A-AZ**

*2-(2-azidoethoxy)-7,12-dihydrobenzo[2,3]azepino[4,5-b]indol-6(5H)-one, KP-An-A-AZ*

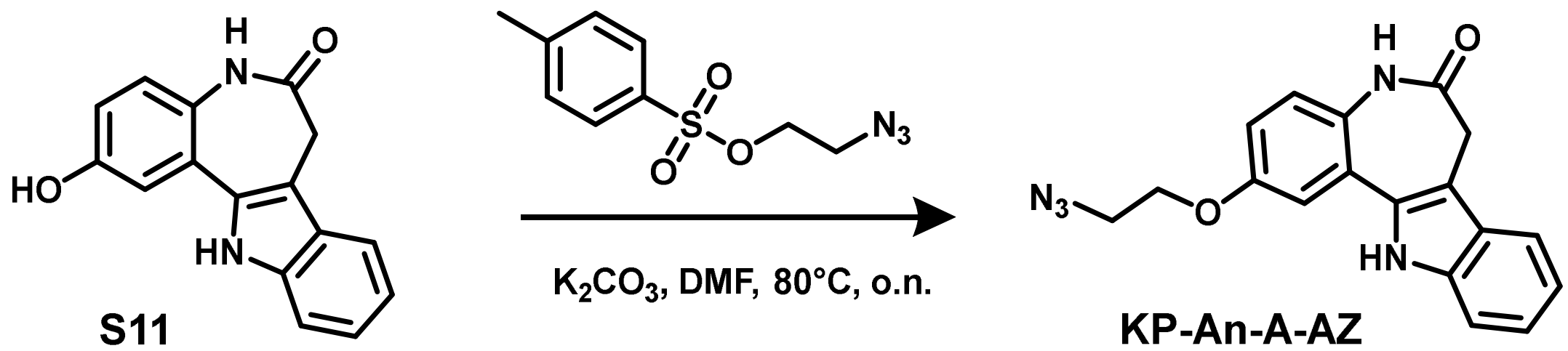


$K_2CO_3$ (18 mg, 0.13 mmol, 1.2 eq) and 0.5 M 1-[(2-azidoethoxy)sulfonyl]-4-methylbenzene (215 μL, 0.11 mmol, 1 eq) were added to a suspension of S9 (28 mg, 0.11 mmol, 1 eq) in dry DMF (0.5 mL) under $N_2$. Reaction was stirred overnight at 80°C. The following day the reaction was loaded directly onto the Biotage Isolera (0 – 100% ACN + 0.1% FA in $H_2O$ + 0.1% FA) for purification to afford KP-An-A-AZ as a yellow solid (0.83mg, 0.003 mmol, 2%). $^1H$ NMR (600 MHz, DMSO-$d_6$) δ 11.57 (s, 1H), 9.91 (s, 1H), 7.66 (d, J = 7.9 Hz, 1H), 7.44 (d, J = 8.1 Hz, 1H), 7.32 (d, J = 2.9 Hz, 1H), 7.20 – 7.16 (m, 2H), 7.08 (td, J = 7.4, 1.0 Hz, 1H), 7.00 (dd, J = 8.8, 2.9 Hz, 1H), 4.30 – 4.24 (m, 2H), 3.71 (t, J = 4.8 Hz, 2H), 3.47 (s, 2H). $^{13}C$ NMR (151 MHz, DMSO-$d_6$) δ 171.3, 154.1, 137.3, 132.4, 129.4, 126.9, 126.5, 123.9, 123.8, 122.3, 119.2, 118.1, 115.2, 111.4, 107.9, 67.1, 49.6, 30.7. HRMS: calculated for $C_{18}H_{15}N_5O_2$ $[M + H]^+$ 334.12, found 334.10. LRMS: found 334.08.

*$^{1}$H NMR for S1*

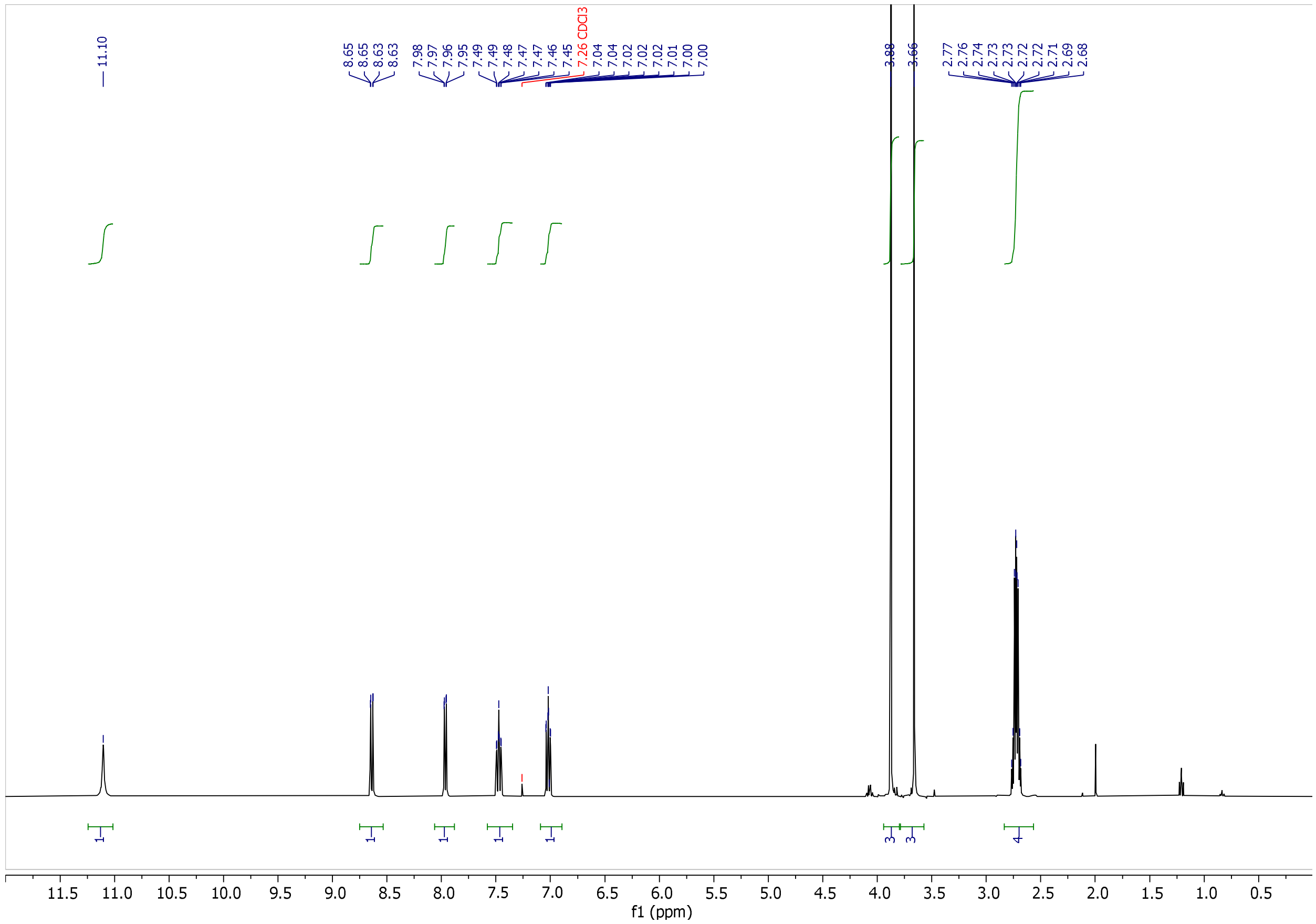

*¹³C NMR for S1*

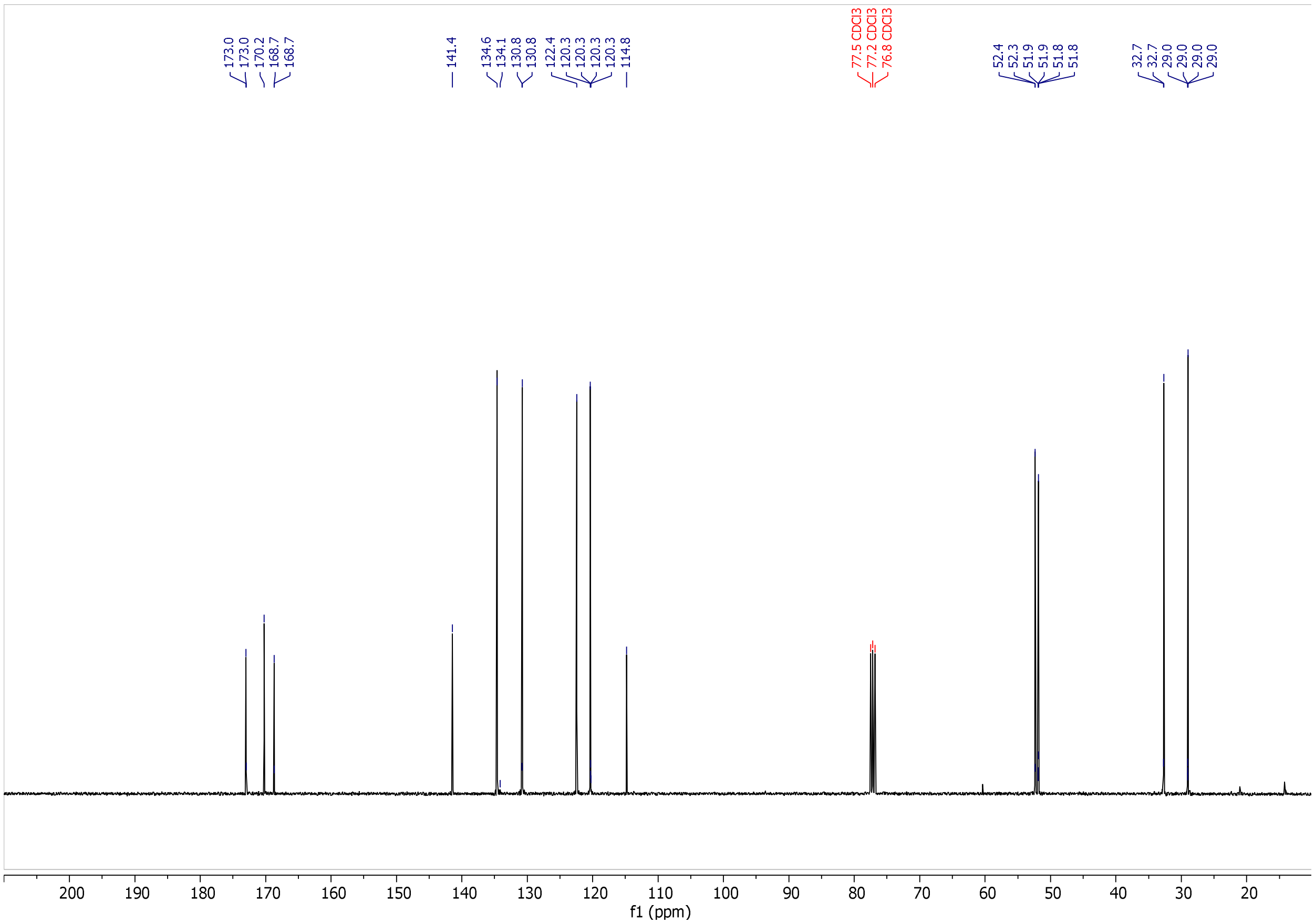

*$^1H$ NMR for S2*

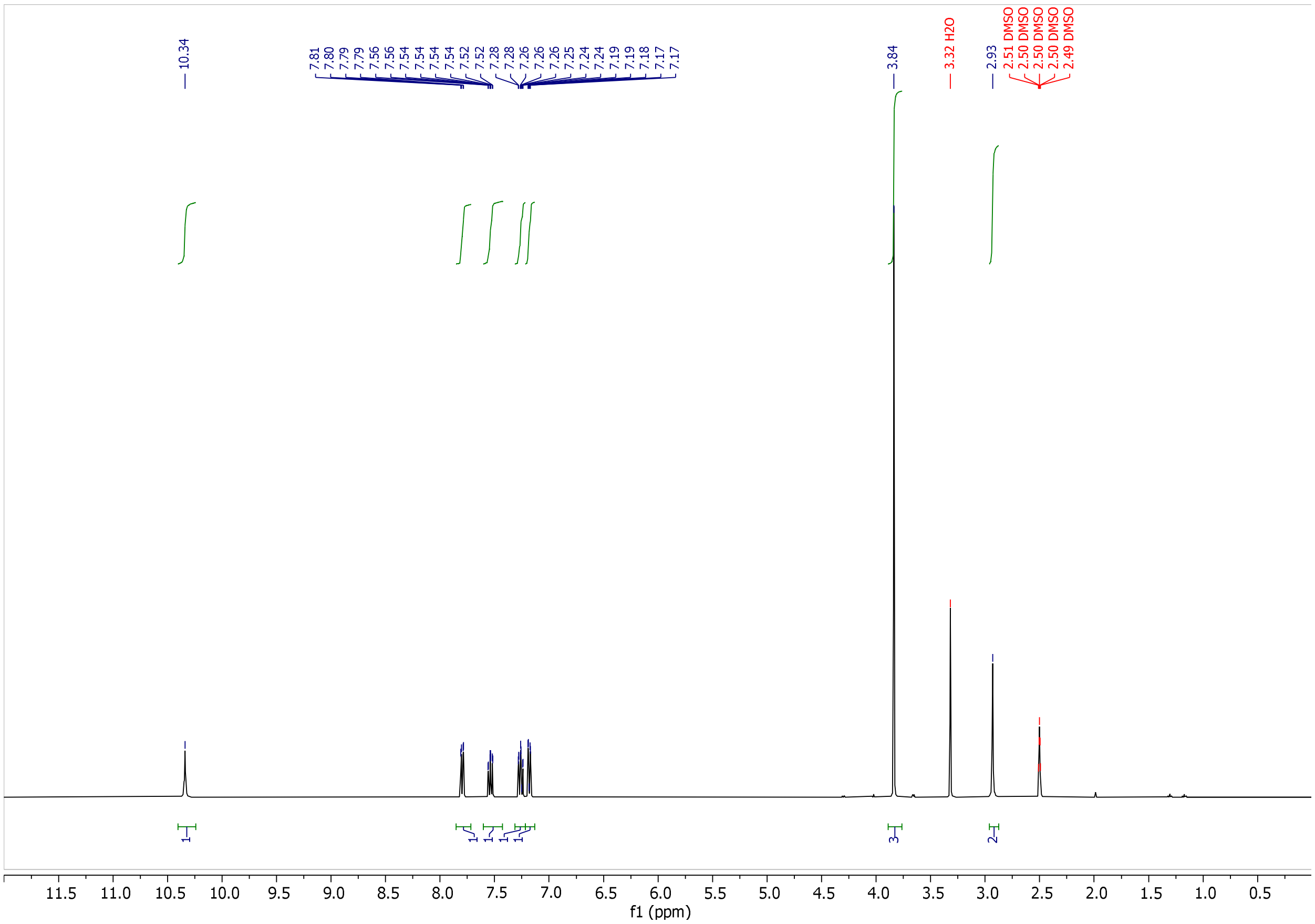
10.34
7.81
7.80
7.79
7.79
7.56
7.56
7.54
7.54
7.54
7.54
7.52
7.52
7.28
7.28
7.26
7.26
7.26
7.25
7.24
7.24
7.19
7.18
7.17
7.17
3.84
3.32 H2O
2.93
2.51 DMSO
2.50 DMSO
2.50 DMSO
2.50 DMSO
2.49 DMSO
1
1
1
1
1
3
2
11.5 11.0 10.5 10.0 9.5 9.0 8.5 8.0 7.5 7.0 6.5 6.0 5.5 5.0 4.5 4.0 3.5 3.0 2.5 2.0 1.5 1.0 0.5
f1 (ppm)

$^{13}C$ NMR for S2

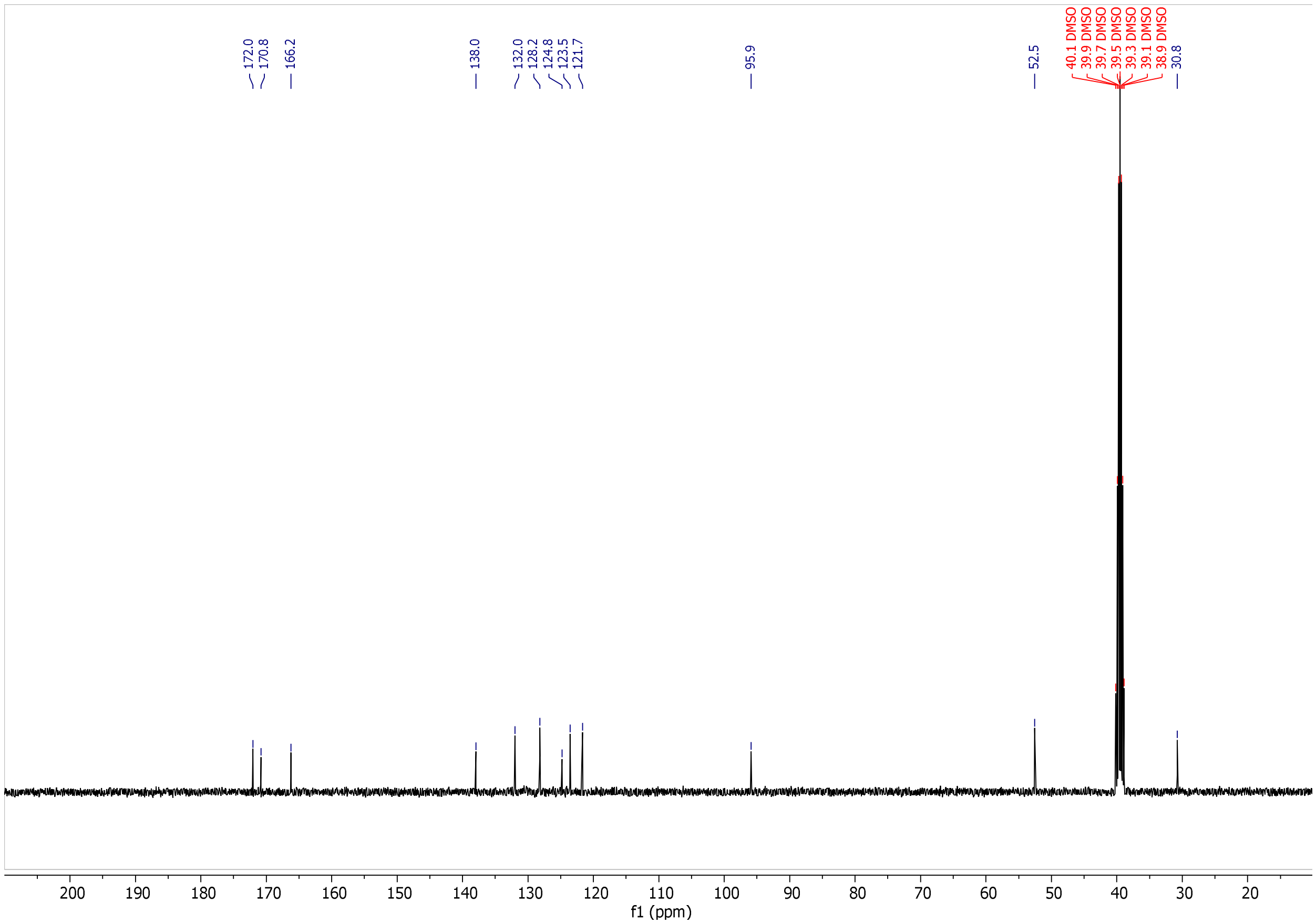

*¹H NMR for S3*

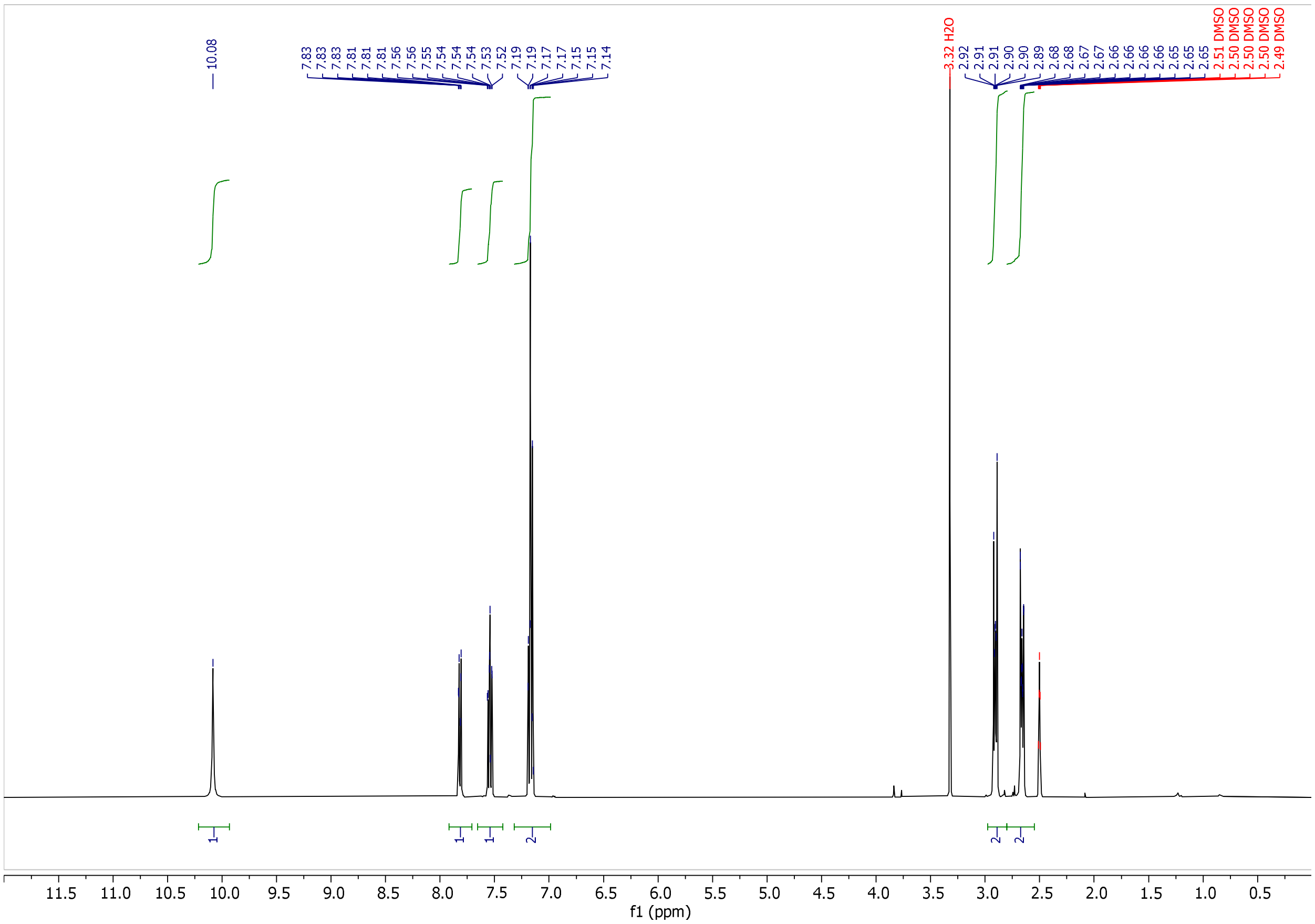

10.08
7.83
7.83
7.83
7.81
7.81
7.81
7.56
7.56
7.55
7.54
7.54
7.54
7.53
7.52
7.19
7.19
7.17
7.17
7.15
7.15
7.14
3.32 H2O
2.92
2.91
2.91
2.90
2.90
2.89
2.68
2.68
2.67
2.67
2.66
2.66
2.66
2.65
2.65
2.65
2.51 DMSO
2.50 DMSO
2.50 DMSO
2.50 DMSO
2.49 DMSO
1
1
1
2
2
2
11.5
11.0
10.5
10.0
9.5
9.0
8.5
8.0
7.5
7.0
6.5
6.0
5.5
5.0
4.5
4.0
3.5
3.0
2.5
2.0
1.5
1.0
0.5
f1 (ppm)

*13C NMR for S3*

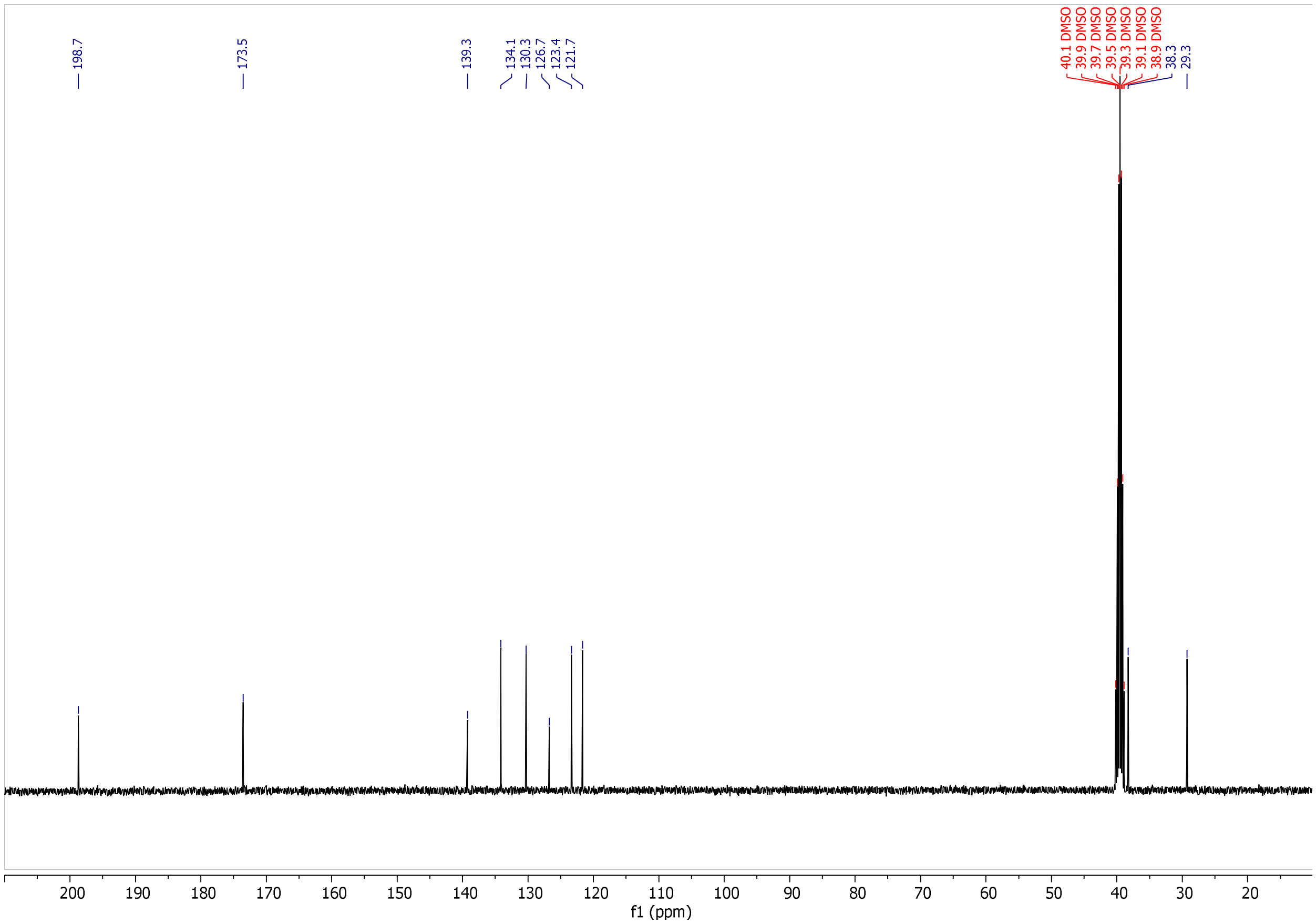

*$^{1}$H NMR for Kenpaullone*

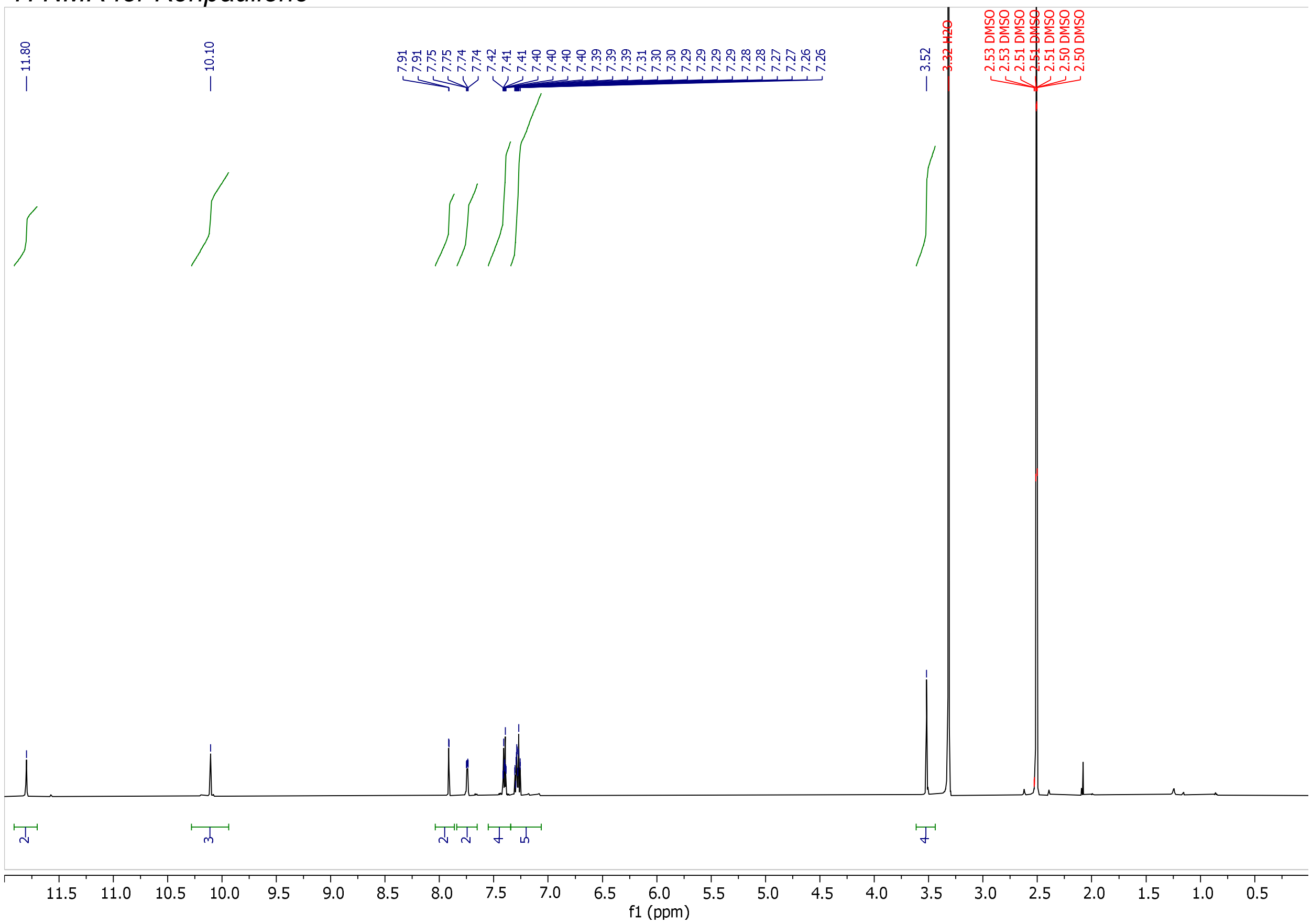

*$^{13}$C NMR for Kenpaullone*

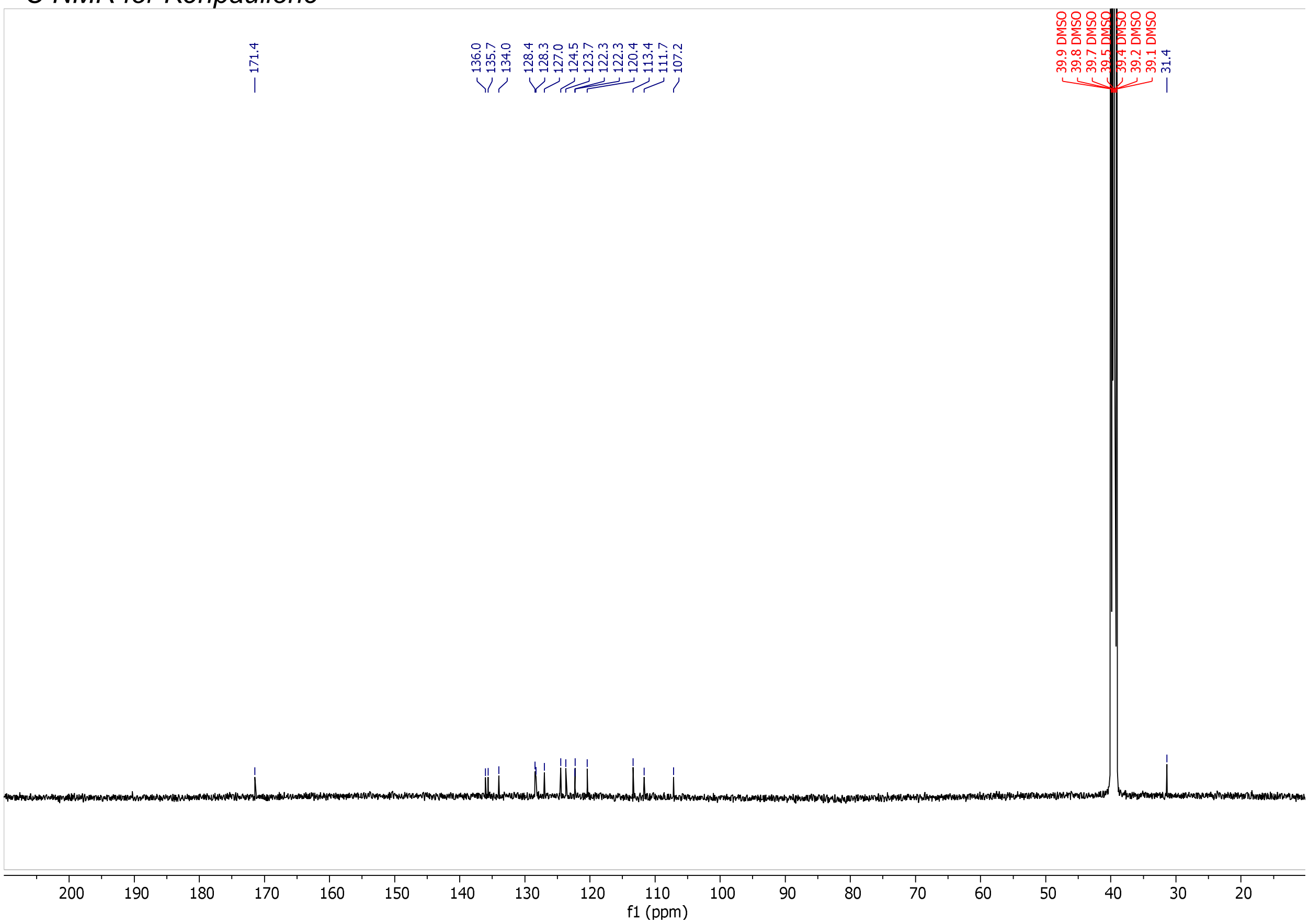

*$^{1}$H NMR for KP-An-A*

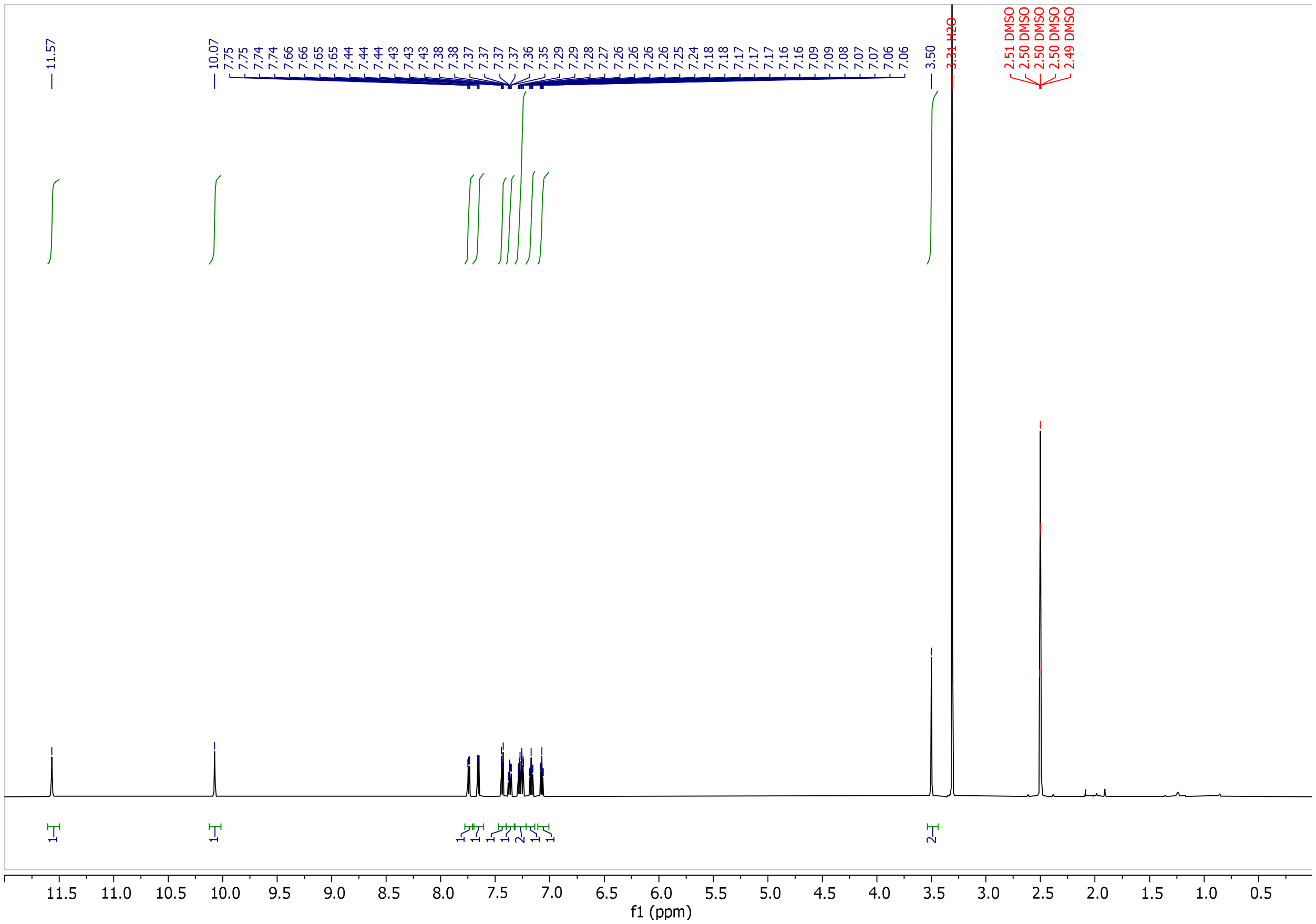

*$^{13}$C NMR for KP-An-A*

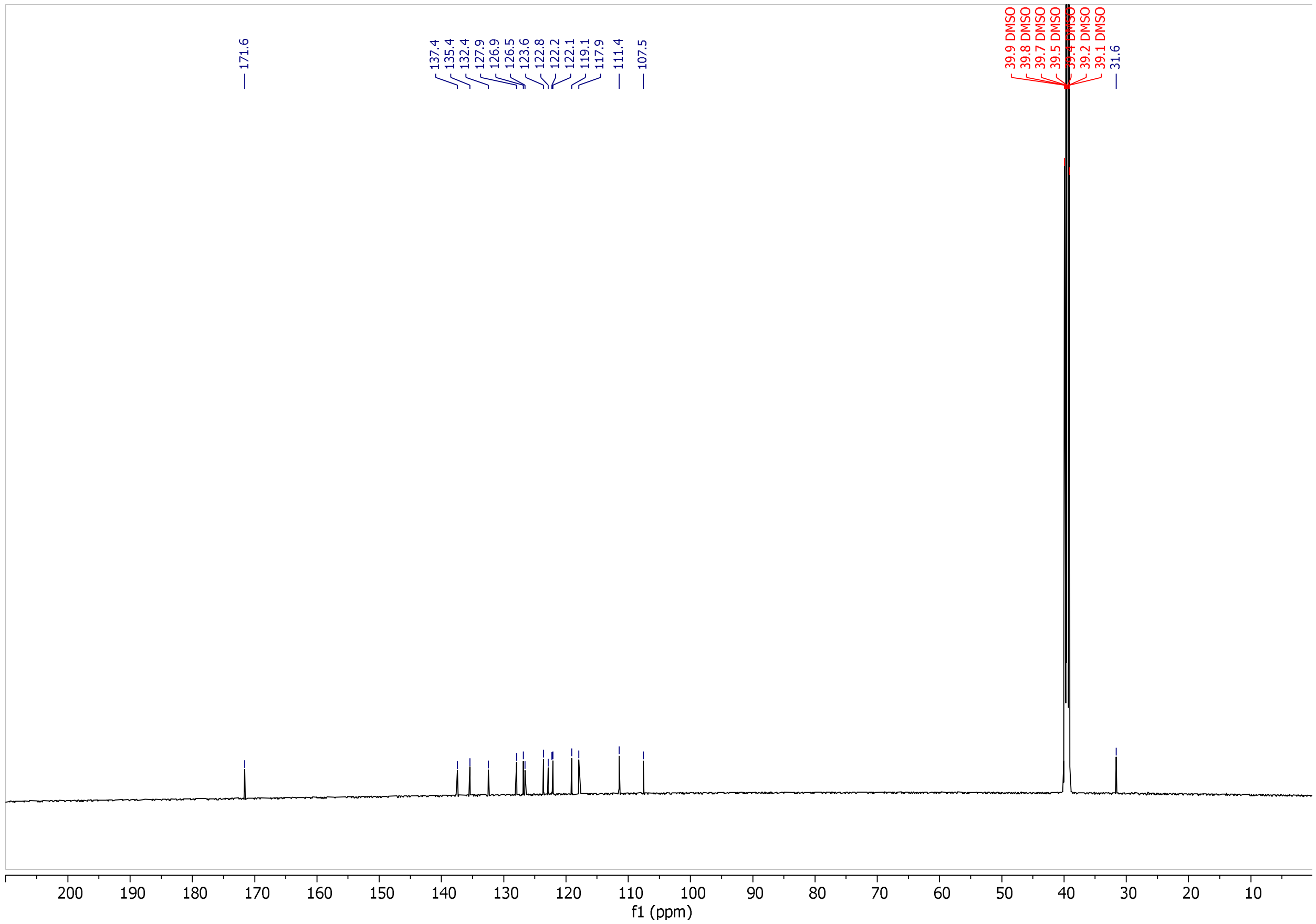

*¹H NMR for KP-An-B*

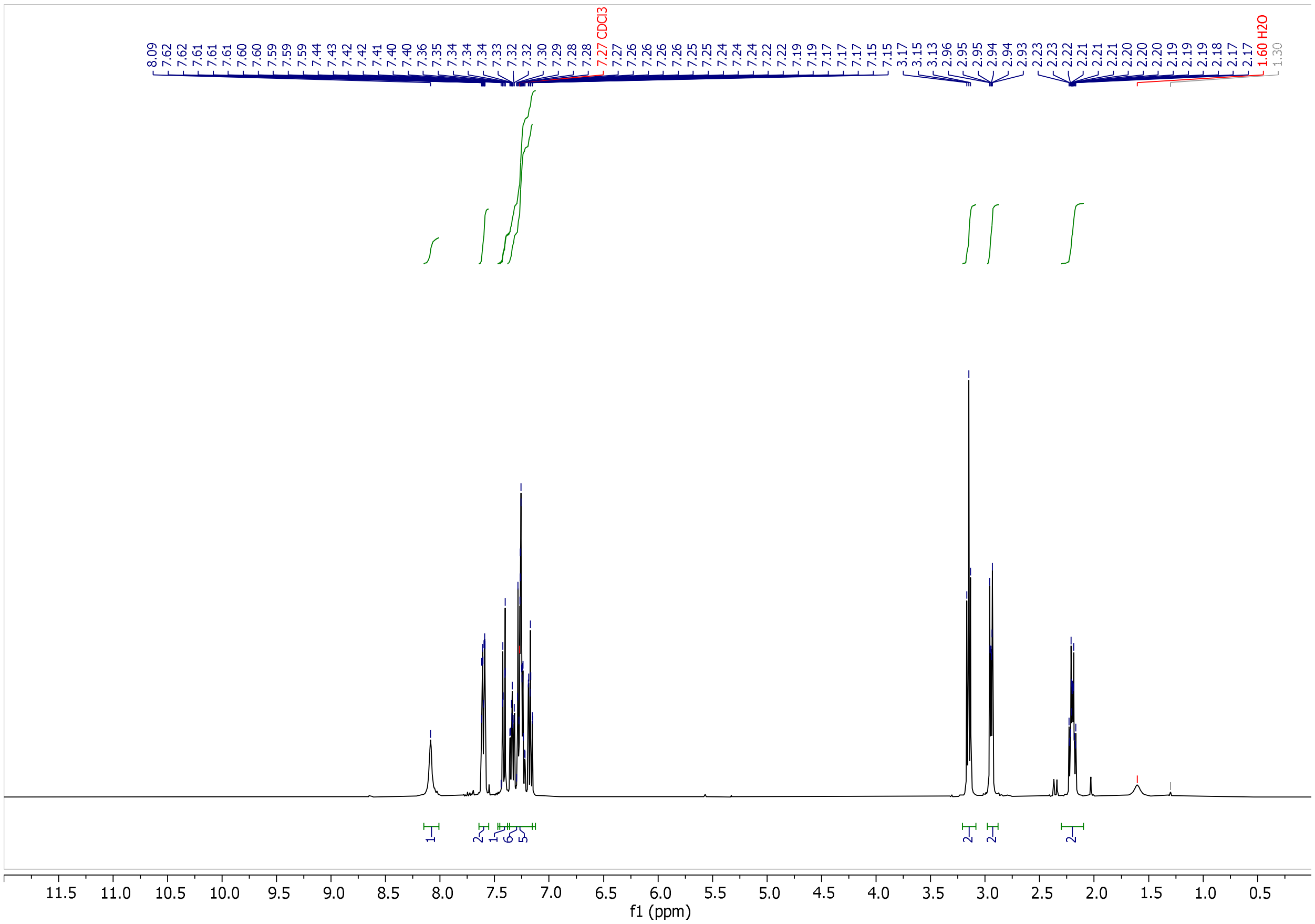

*$^{1}$H NMR for S4*

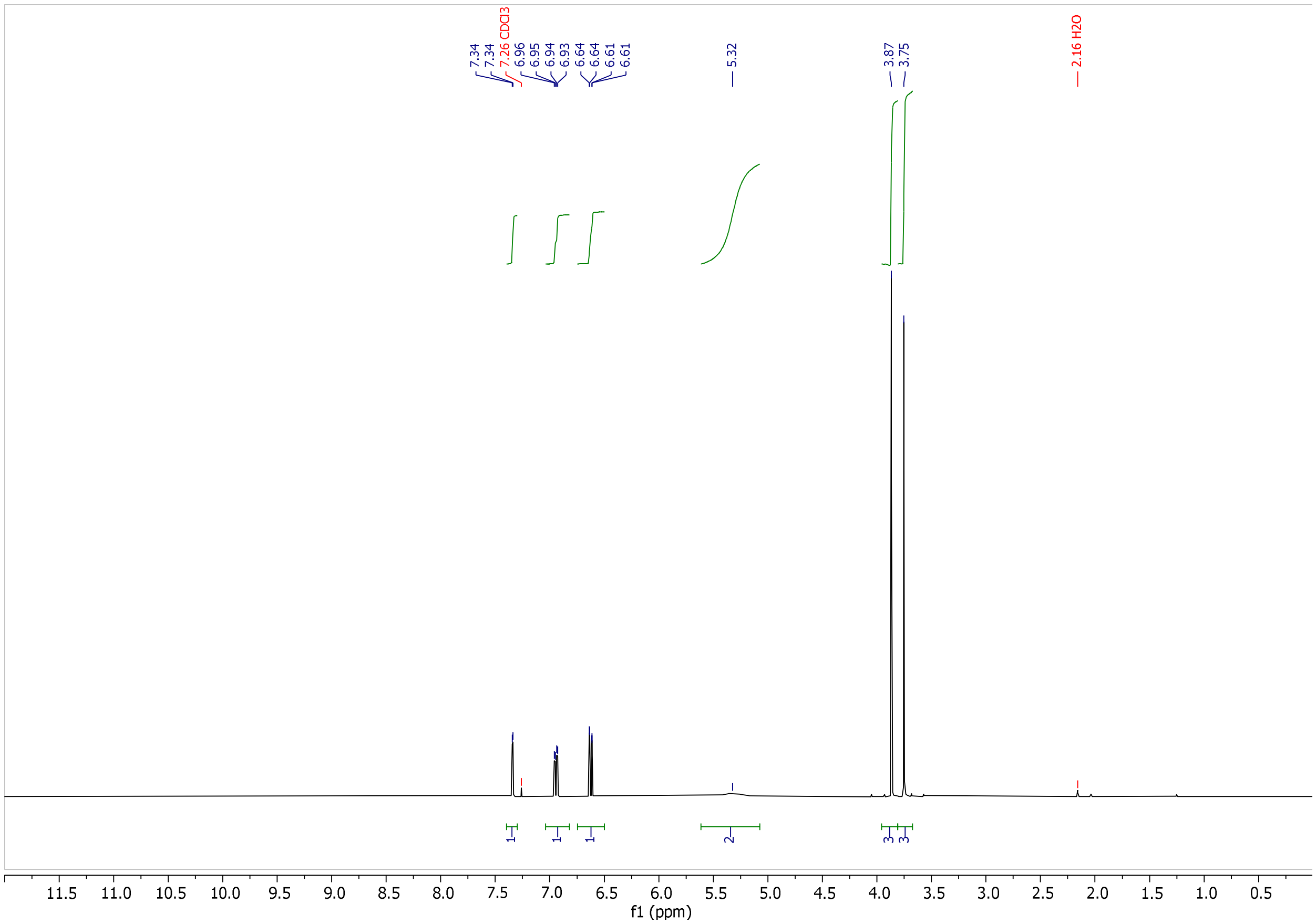

7.34
7.34
7.26 CDCl3
6.96
6.95
6.94
6.93
6.64
6.64
6.61
6.61
5.32
3.87
3.75
2.16 H2O
1
1
1
2
3
3
11.5 11.0 10.5 10.0 9.5 9.0 8.5 8.0 7.5 7.0 6.5 6.0 5.5 5.0 4.5 4.0 3.5 3.0 2.5 2.0 1.5 1.0 0.5
f1 (ppm)

*$^{13}$C NMR for S4*

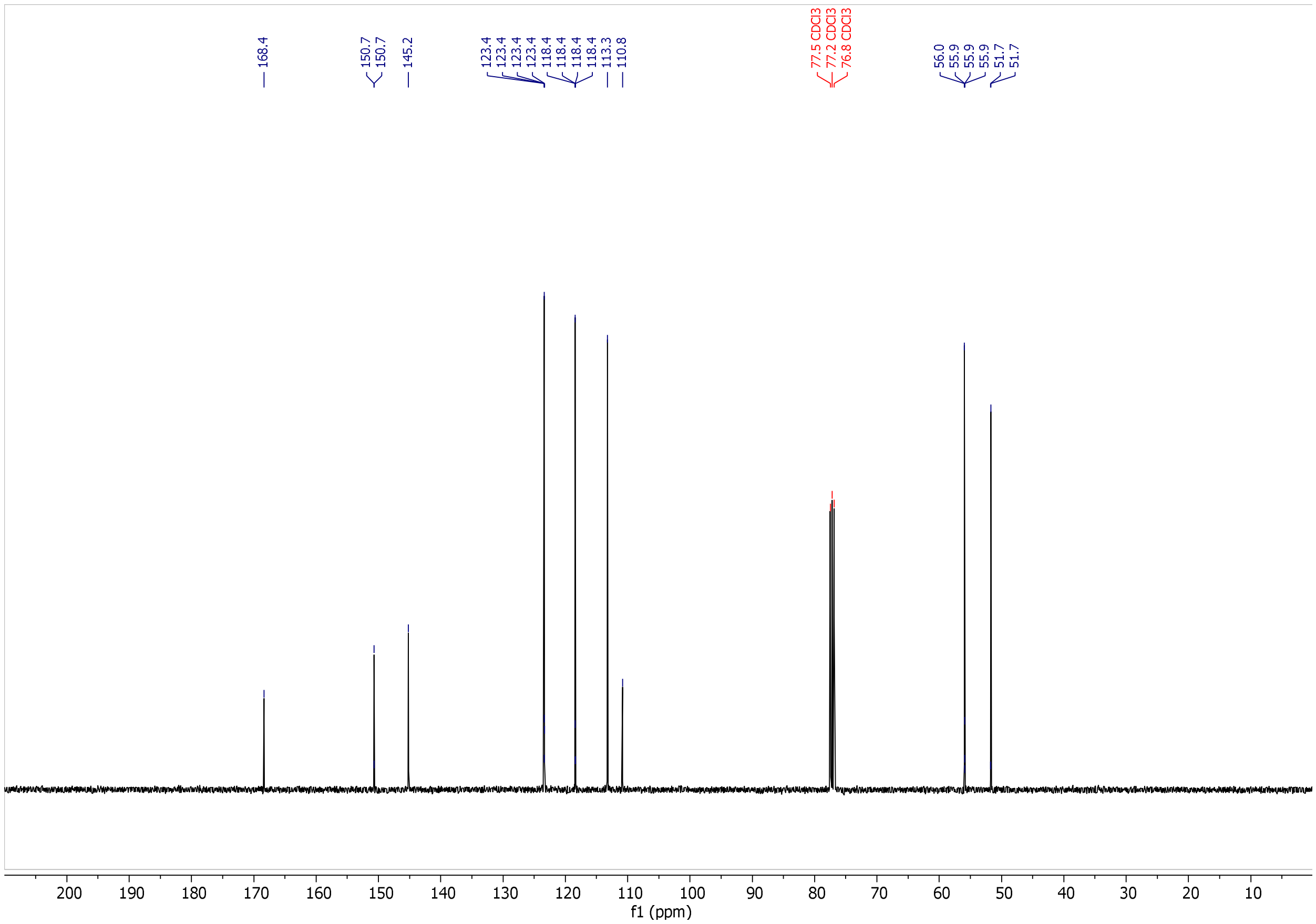

*¹H NMR for S5*

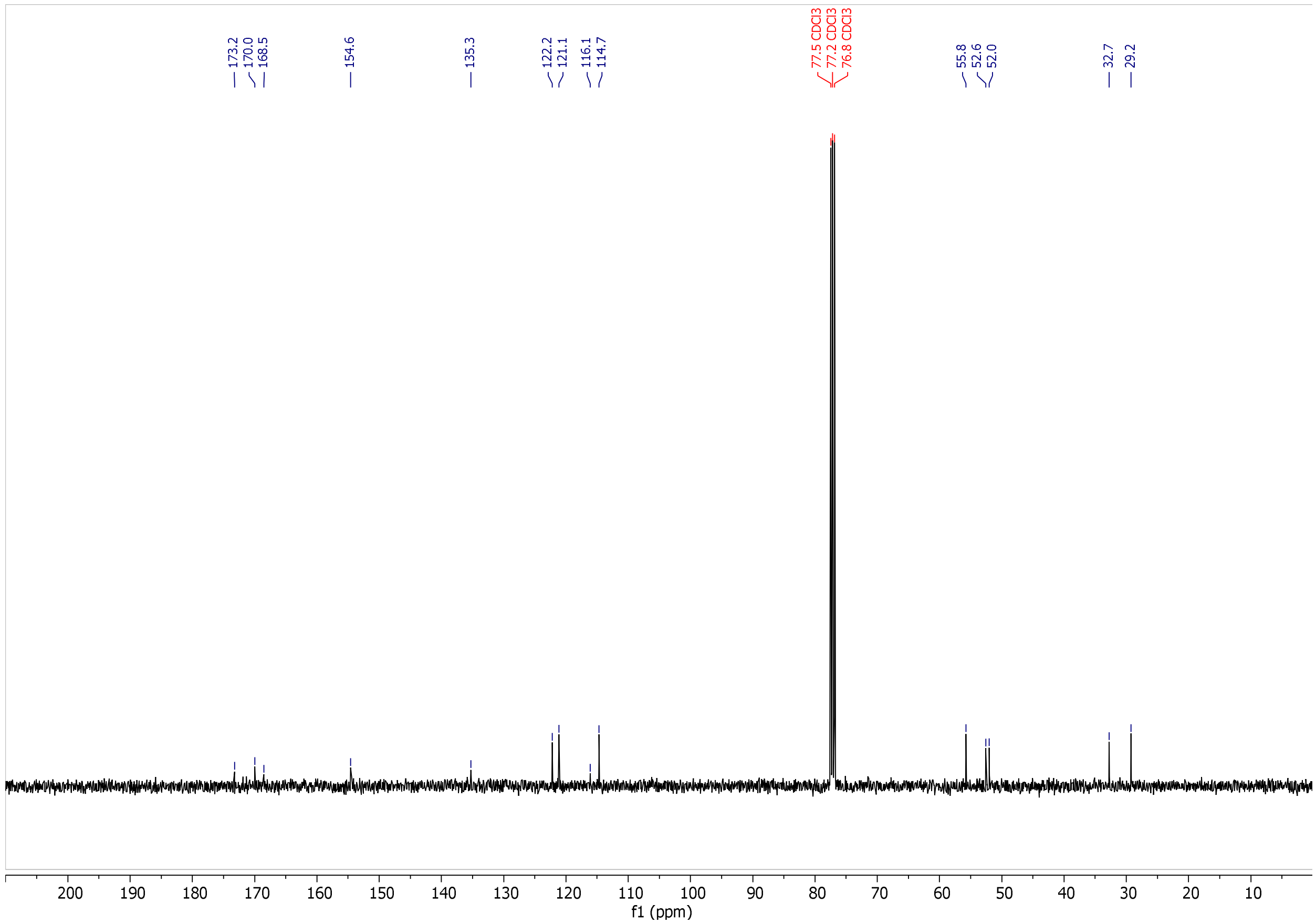
173.2
170.0
168.5
154.6
135.3
122.2
121.1
116.1
114.7
77.5 CDCl3
77.2 CDCl3
76.8 CDCl3
55.8
52.6
52.0
32.7
29.2
200 190 180 170 160 150 140 130 120 110 100 90 80 70 60 50 40 30 20 10
f1 (ppm)

*$^{13}$C NMR for S5*

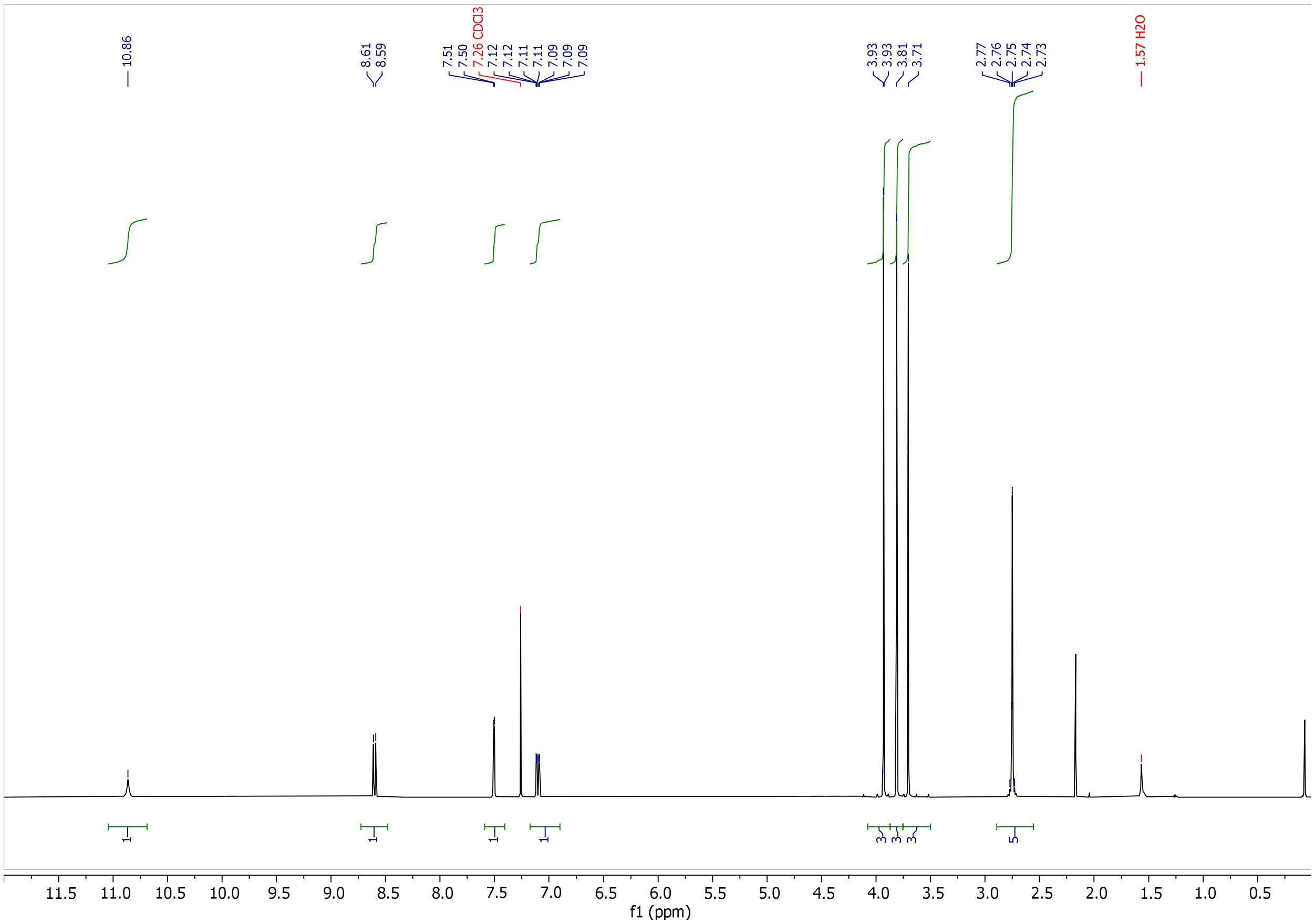

*$^{1}$H NMR for S6*

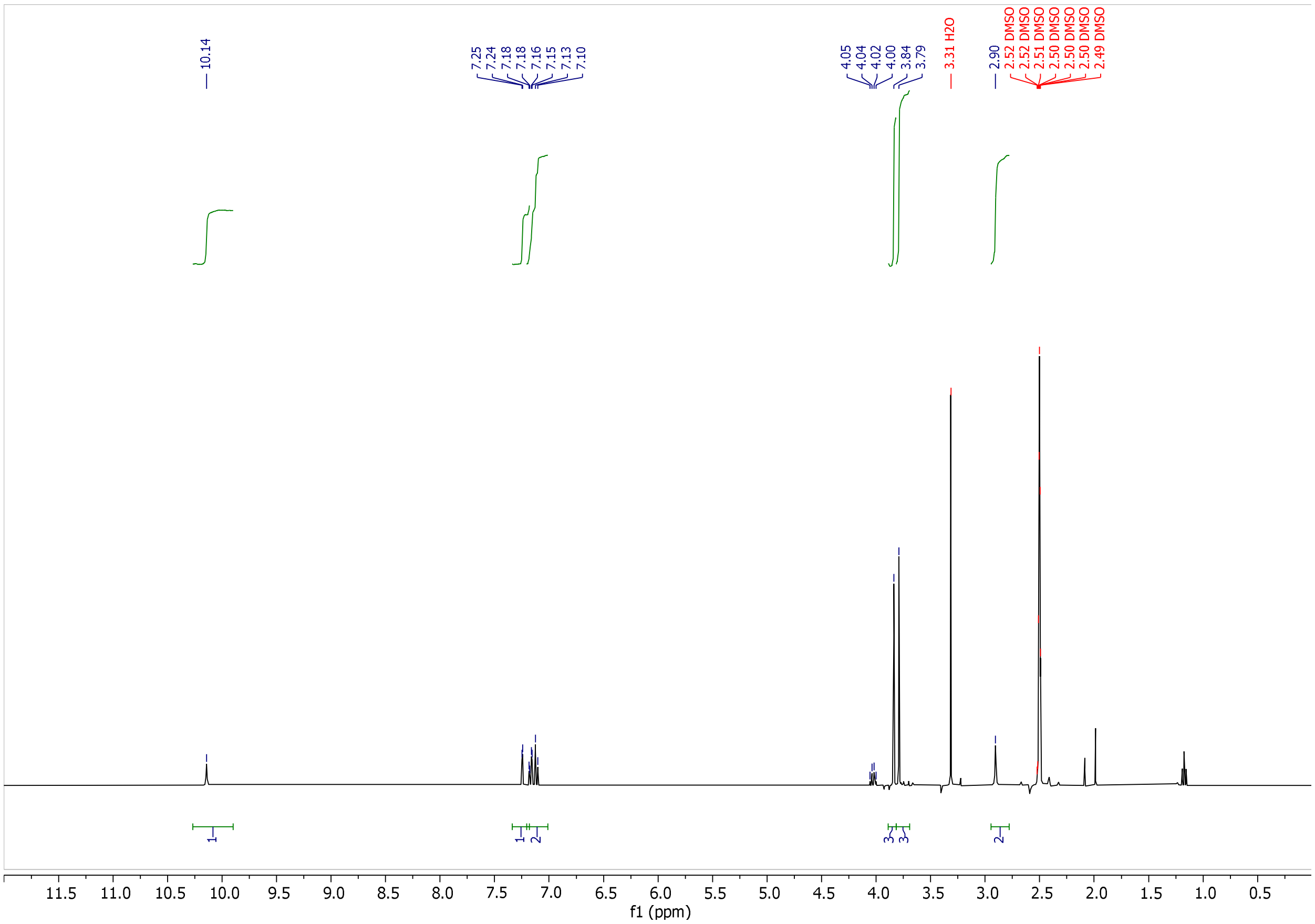
10.14
7.25
7.24
7.18
7.18
7.16
7.15
7.13
7.10
4.05
4.04
4.02
4.00
3.84
3.79
3.31 H2O
2.90
2.52 DMSO
2.52 DMSO
2.51 DMSO
2.50 DMSO
2.50 DMSO
2.50 DMSO
2.49 DMSO
1
1
2
3
3
2
11.5 11.0 10.5 10.0 9.5 9.0 8.5 8.0 7.5 7.0 6.5 6.0 5.5 5.0 4.5 4.0 3.5 3.0 2.5 2.0 1.5 1.0 0.5
f1 (ppm)

*¹³C NMR for S6*

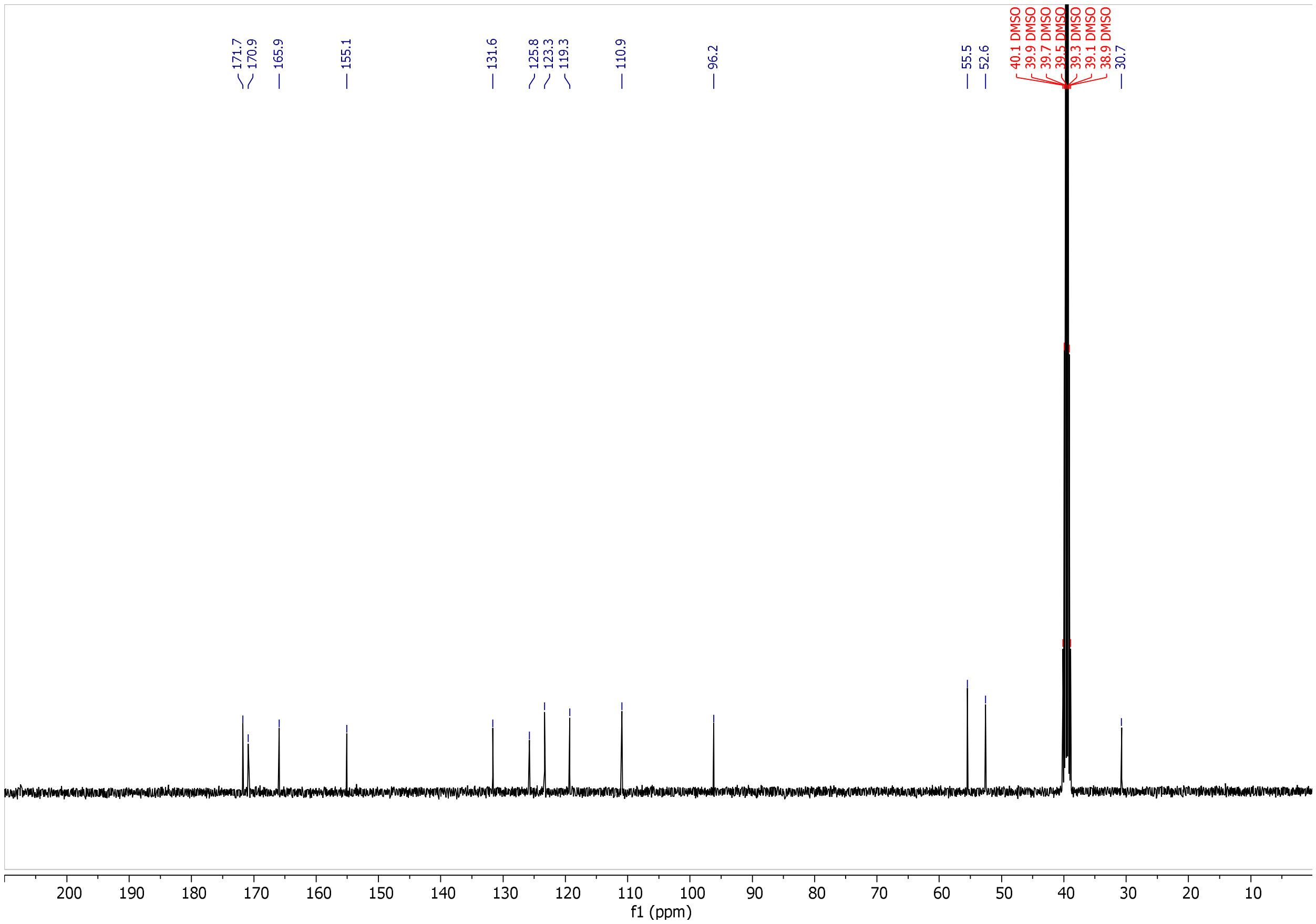

*¹H NMR for S7*

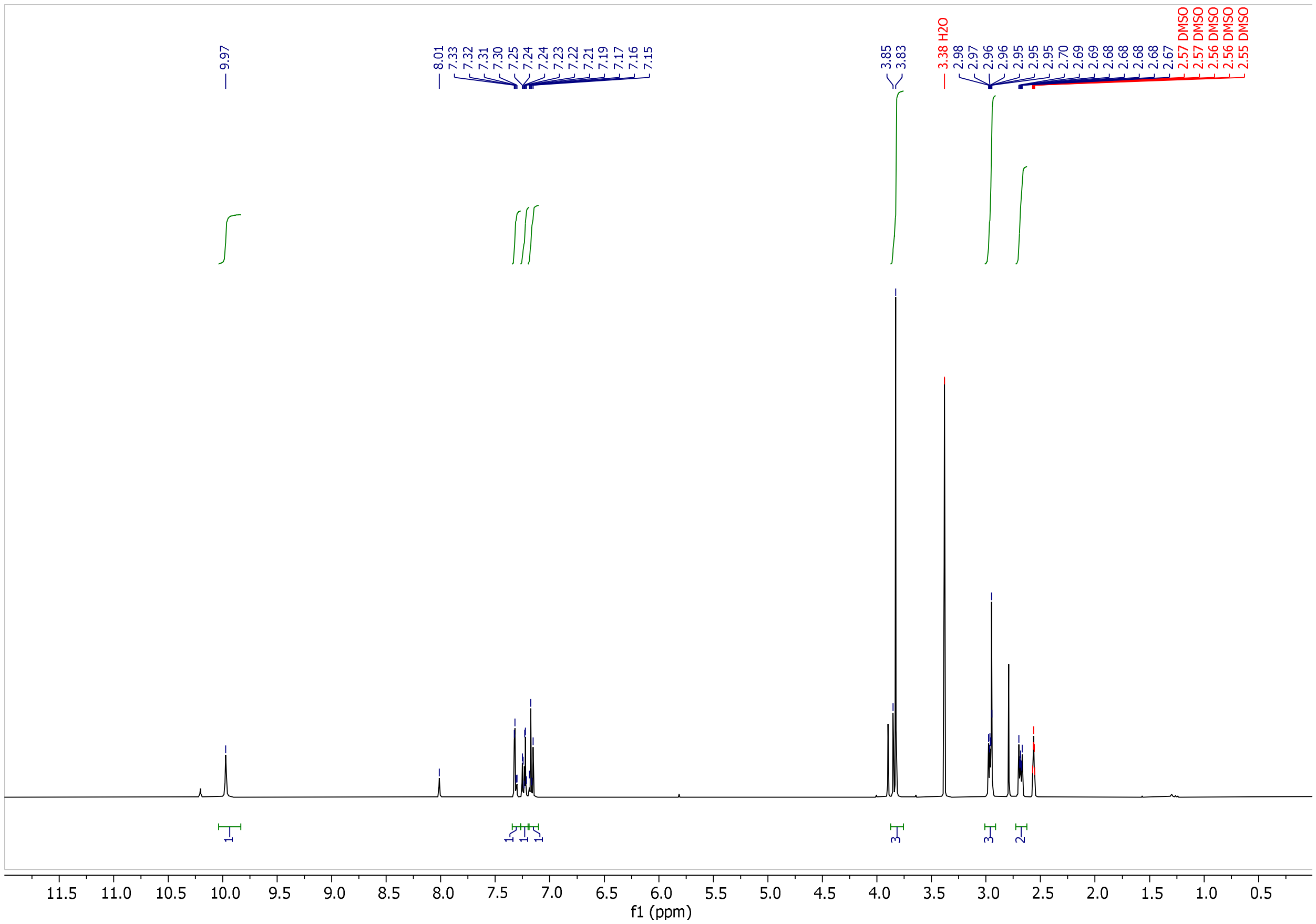

*$^{13}C$ NMR for S7*

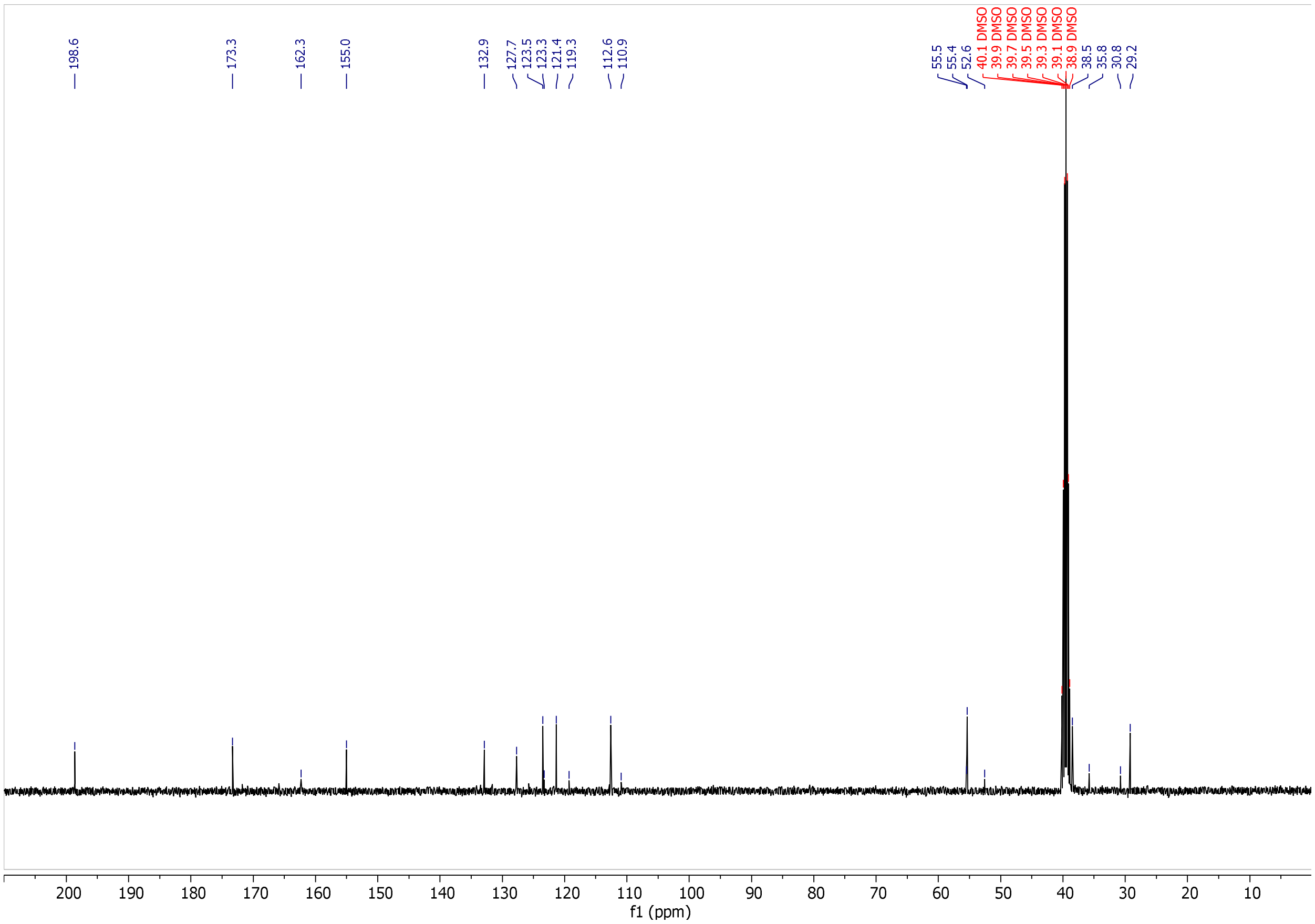

*¹H NMR for S8*

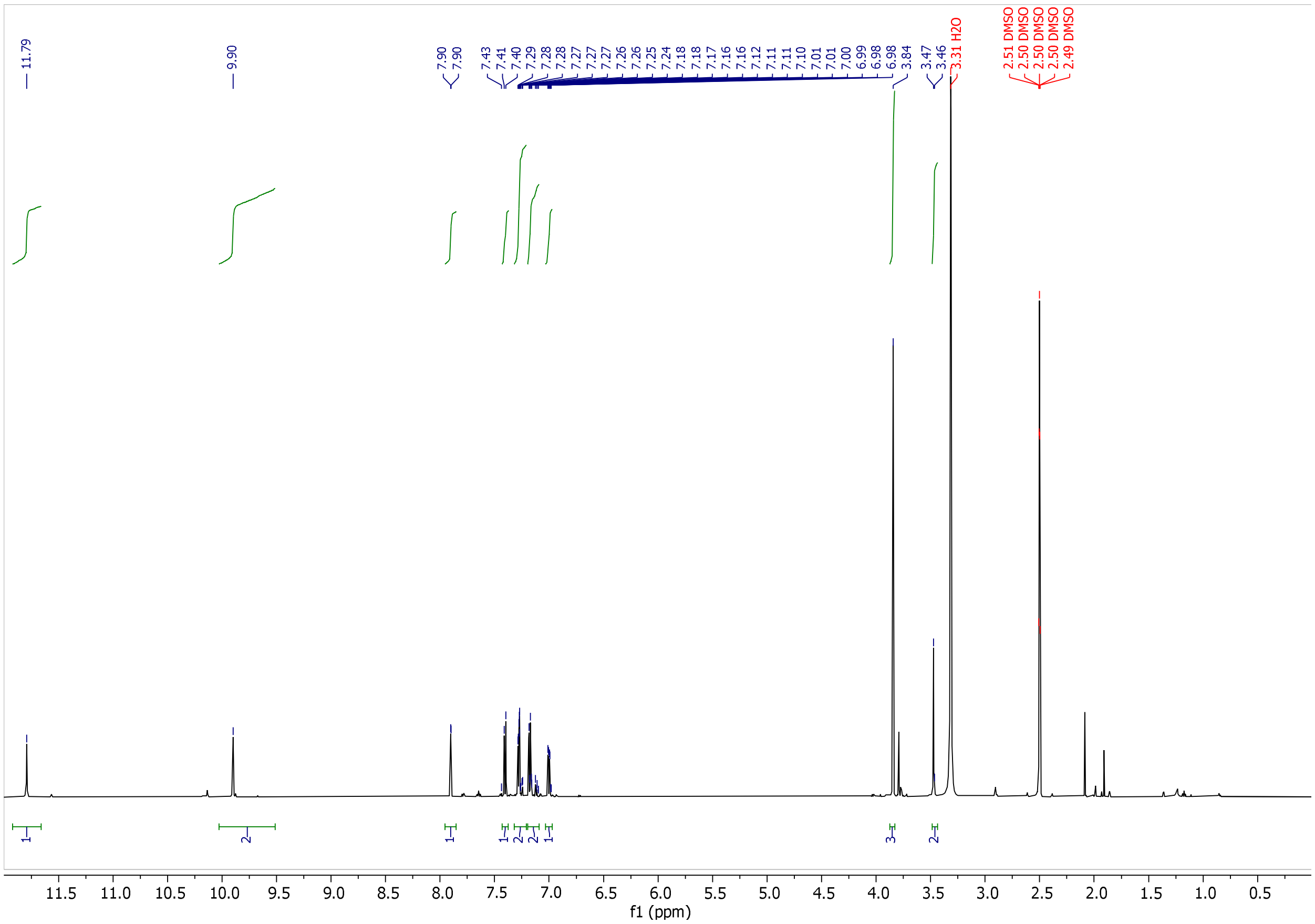

11.79
9.90
7.90
7.90
7.43
7.41
7.40
7.29
7.28
7.28
7.27
7.27
7.27
7.26
7.26
7.25
7.24
7.18
7.18
7.17
7.16
7.16
7.12
7.11
7.11
7.10
7.01
7.01
7.00
6.99
6.98
6.98
3.84
3.47
3.46
3.31 H2O
2.51 DMSO
2.50 DMSO
2.50 DMSO
2.50 DMSO
2.49 DMSO
f1 (ppm)

*$^{13}$C NMR for S8*

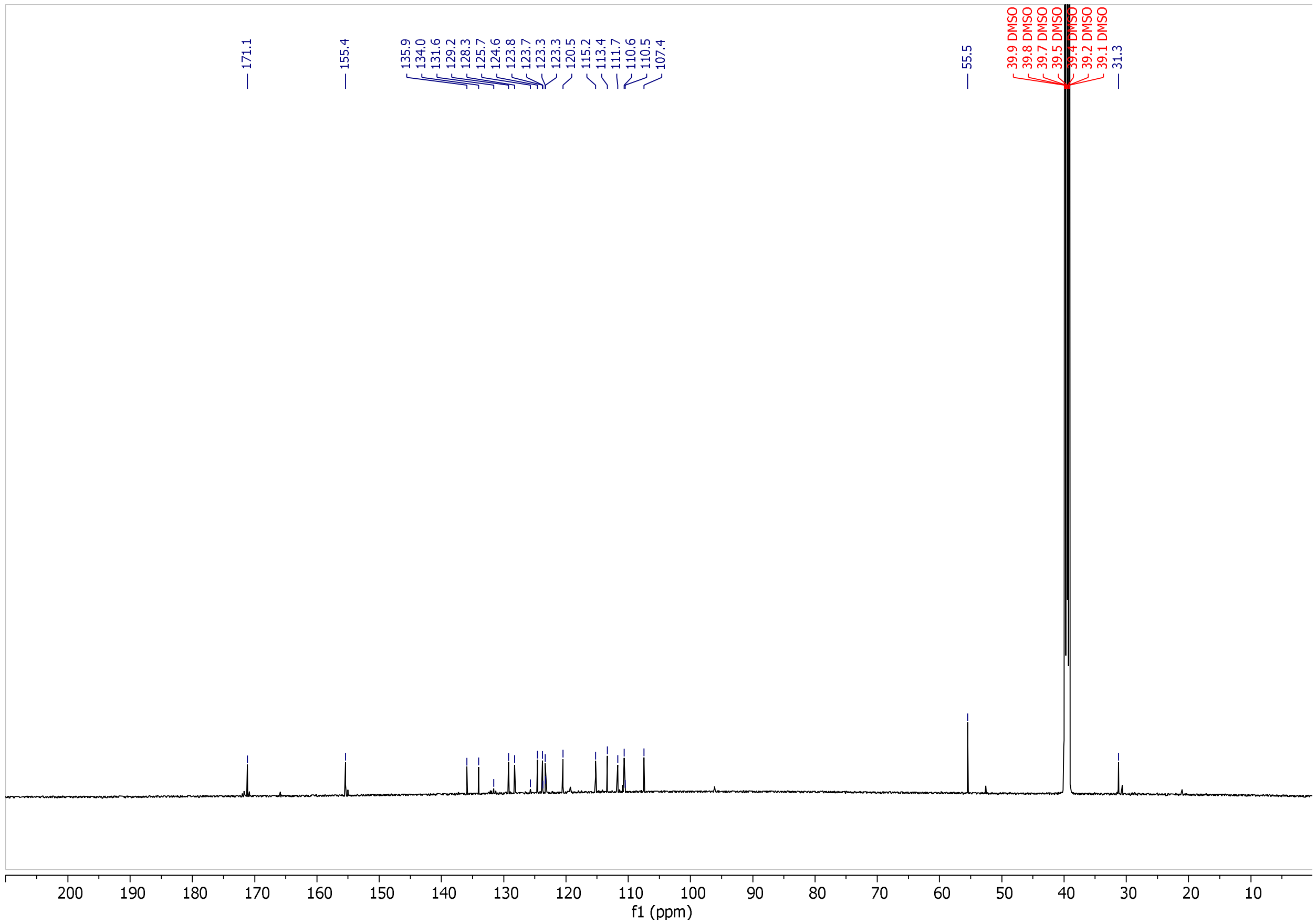

*¹H NMR for S9*

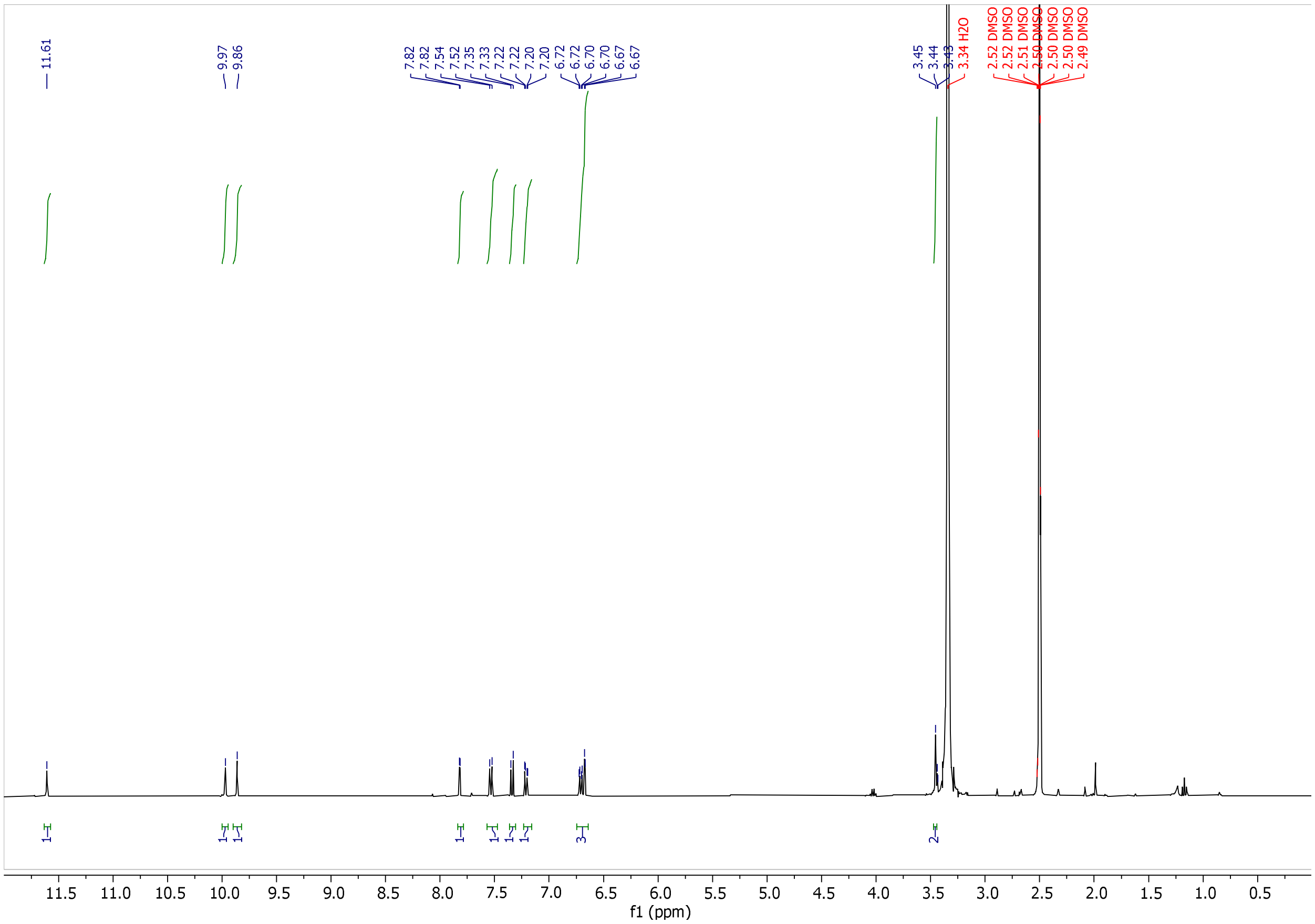

*$^{13}C$ NMR for S9*

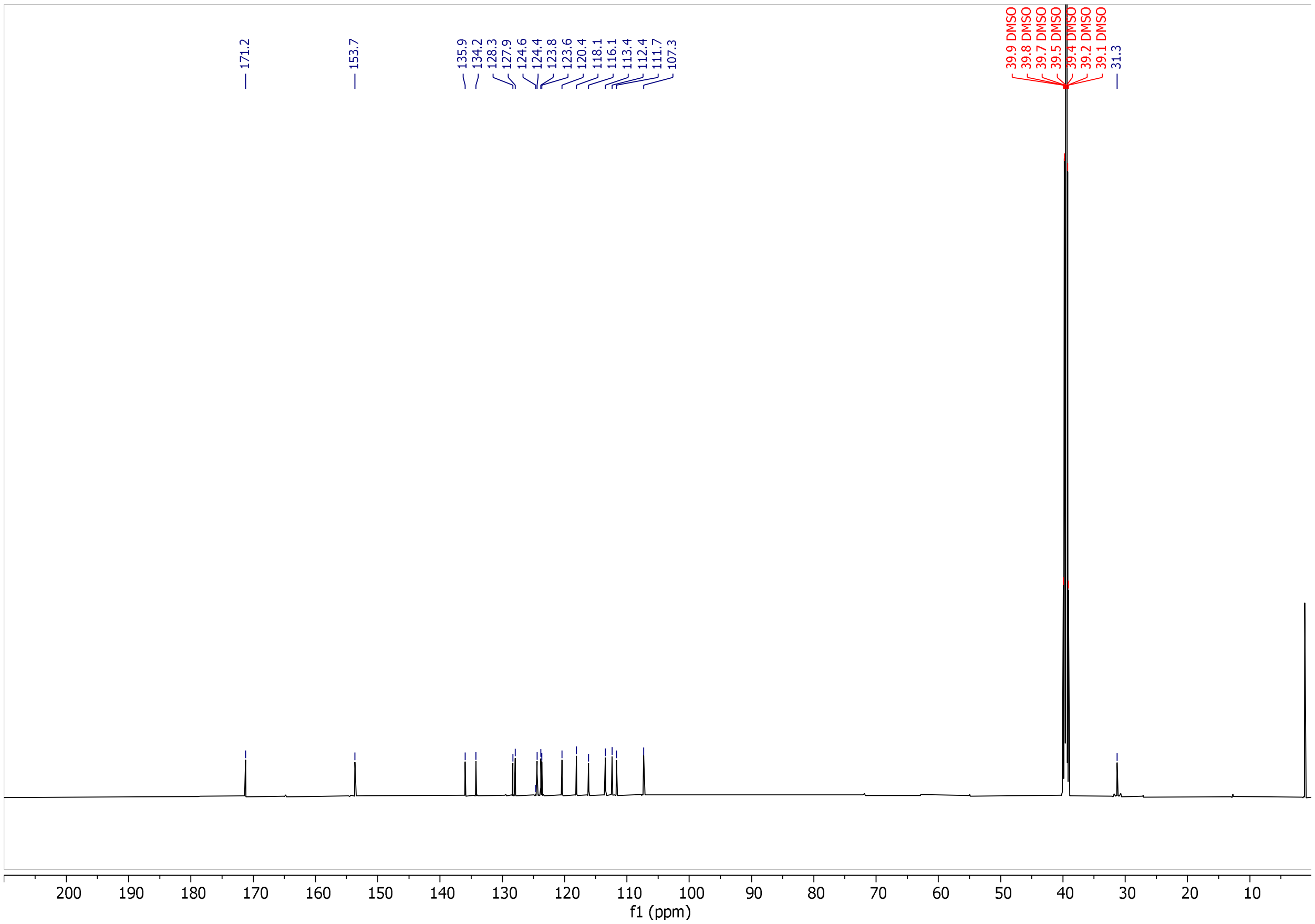

*¹H NMR for KP-DA*

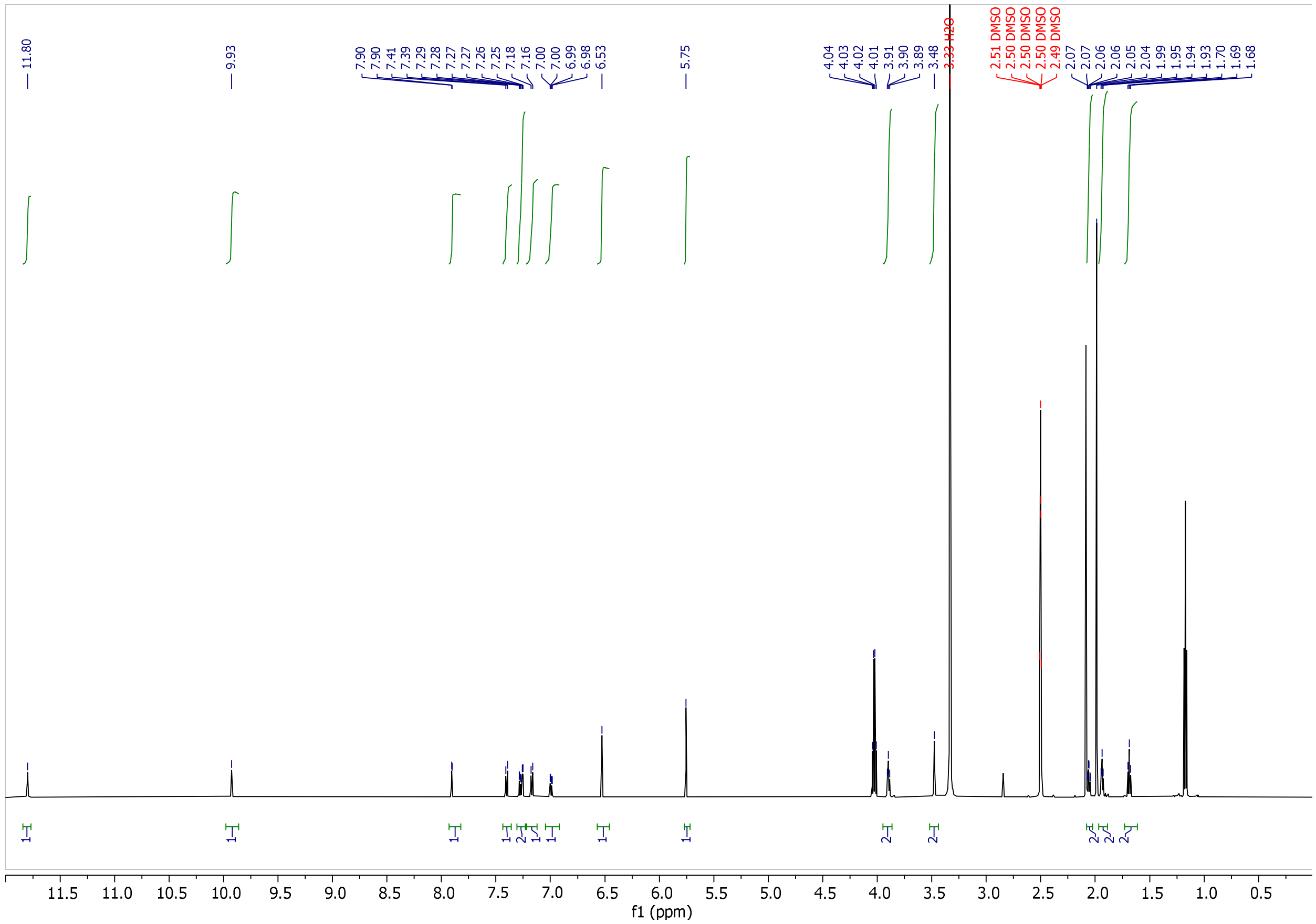

*$^{13}$C NMR for KP-DA*

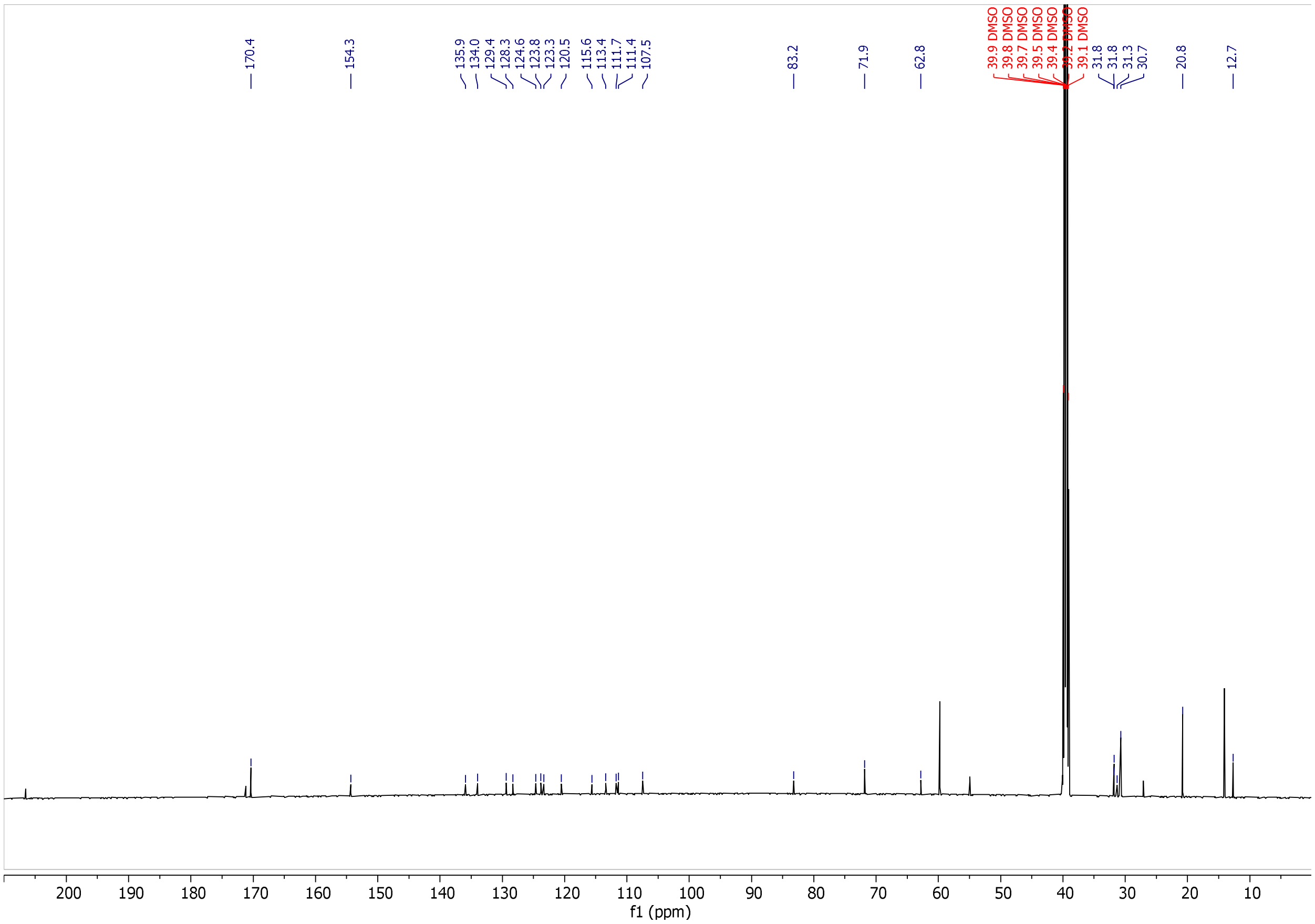

*$^1$H NMR for S10*

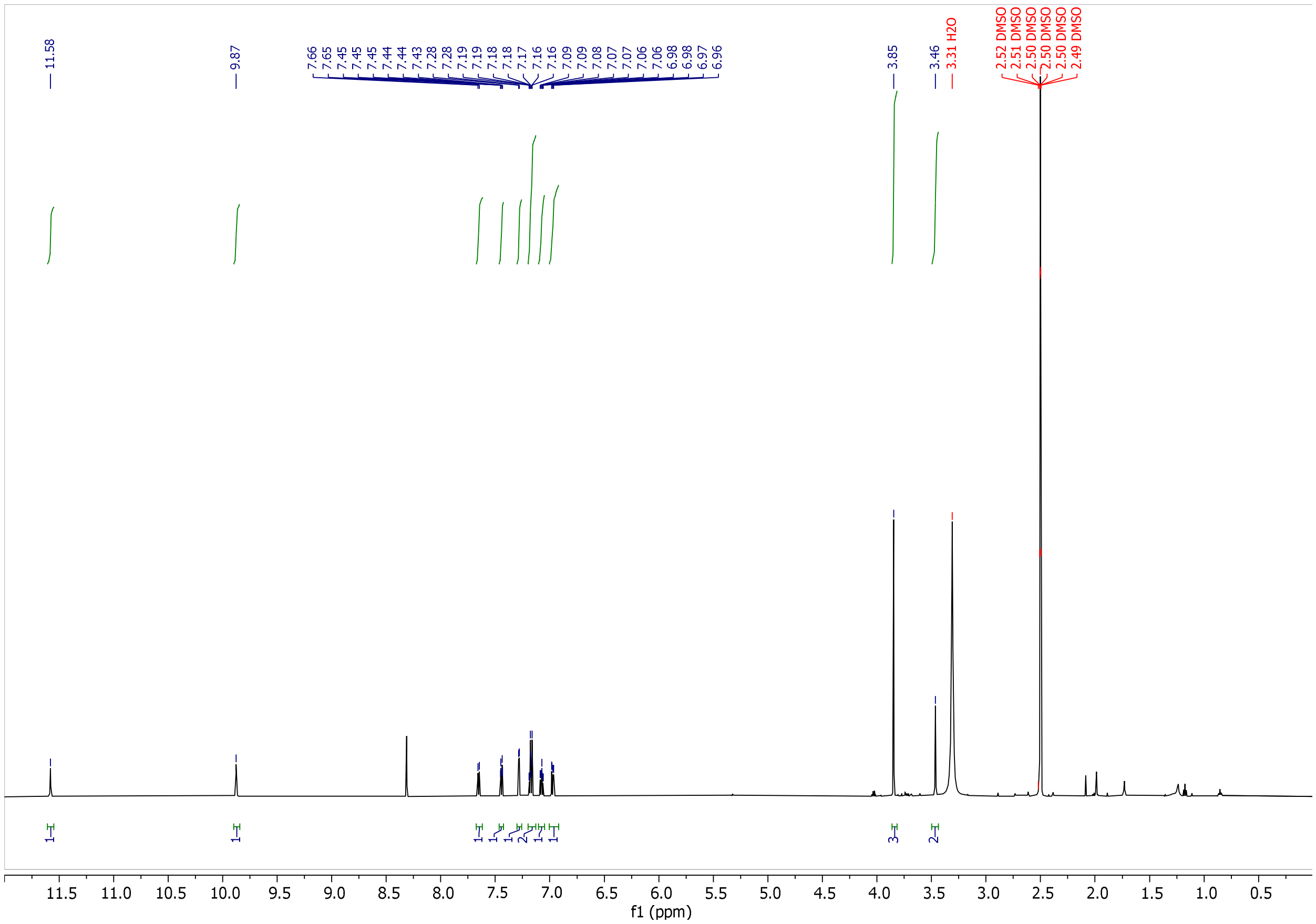

*$^{13}$C NMR for S10*

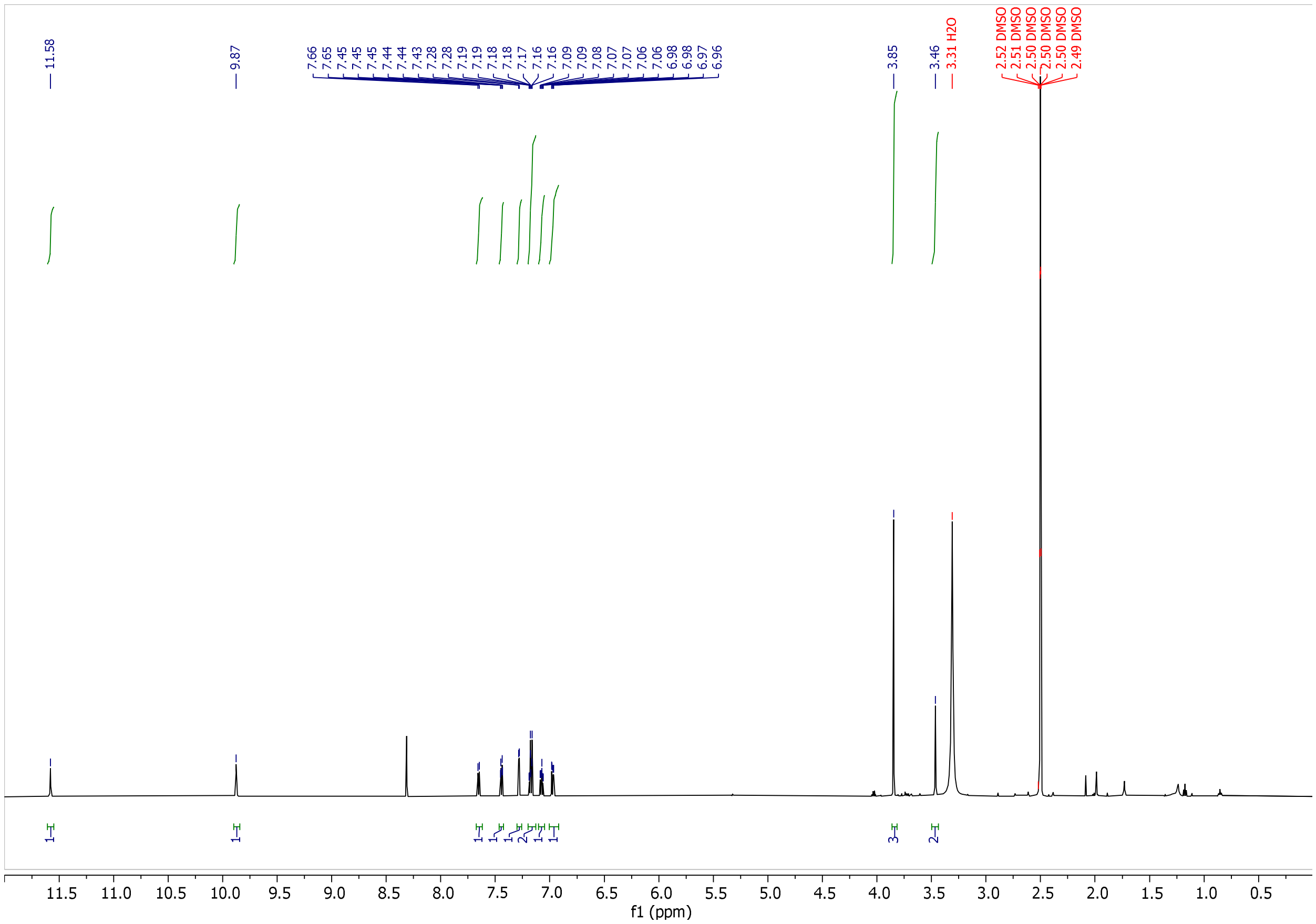

*¹H NMR for S11*

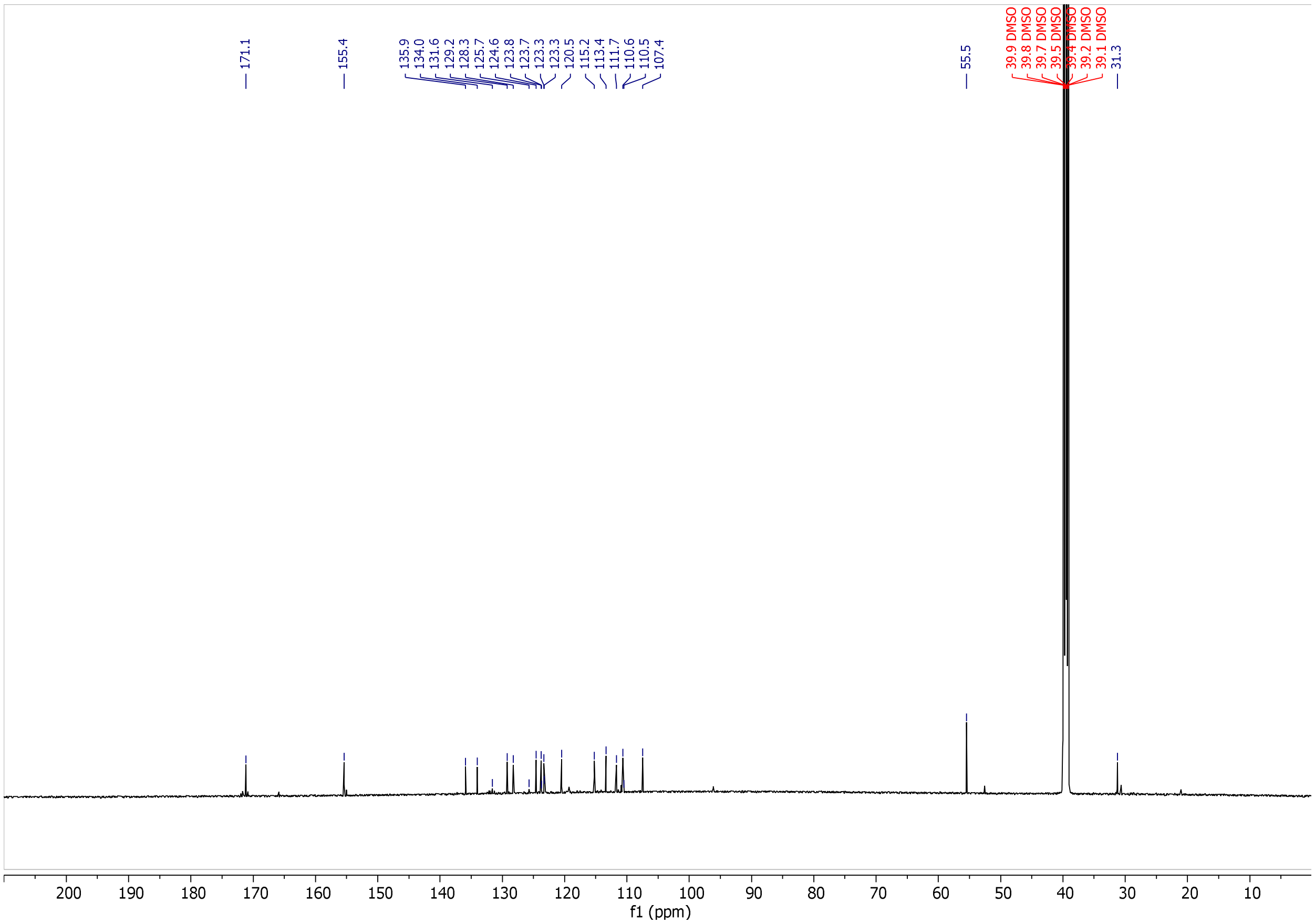

*¹³C NMR for S11*

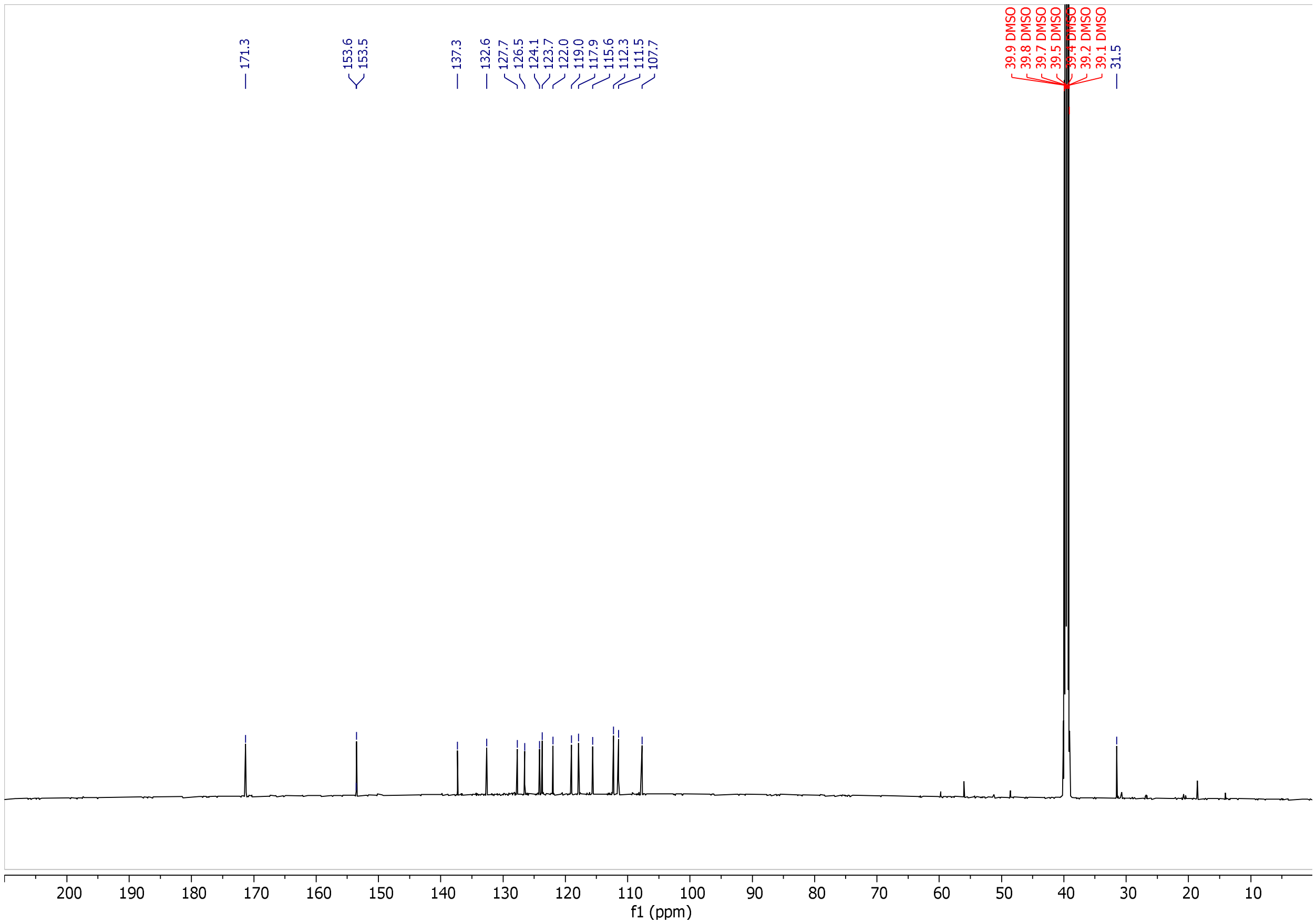

*¹H NMR for KP-An-A-DA*

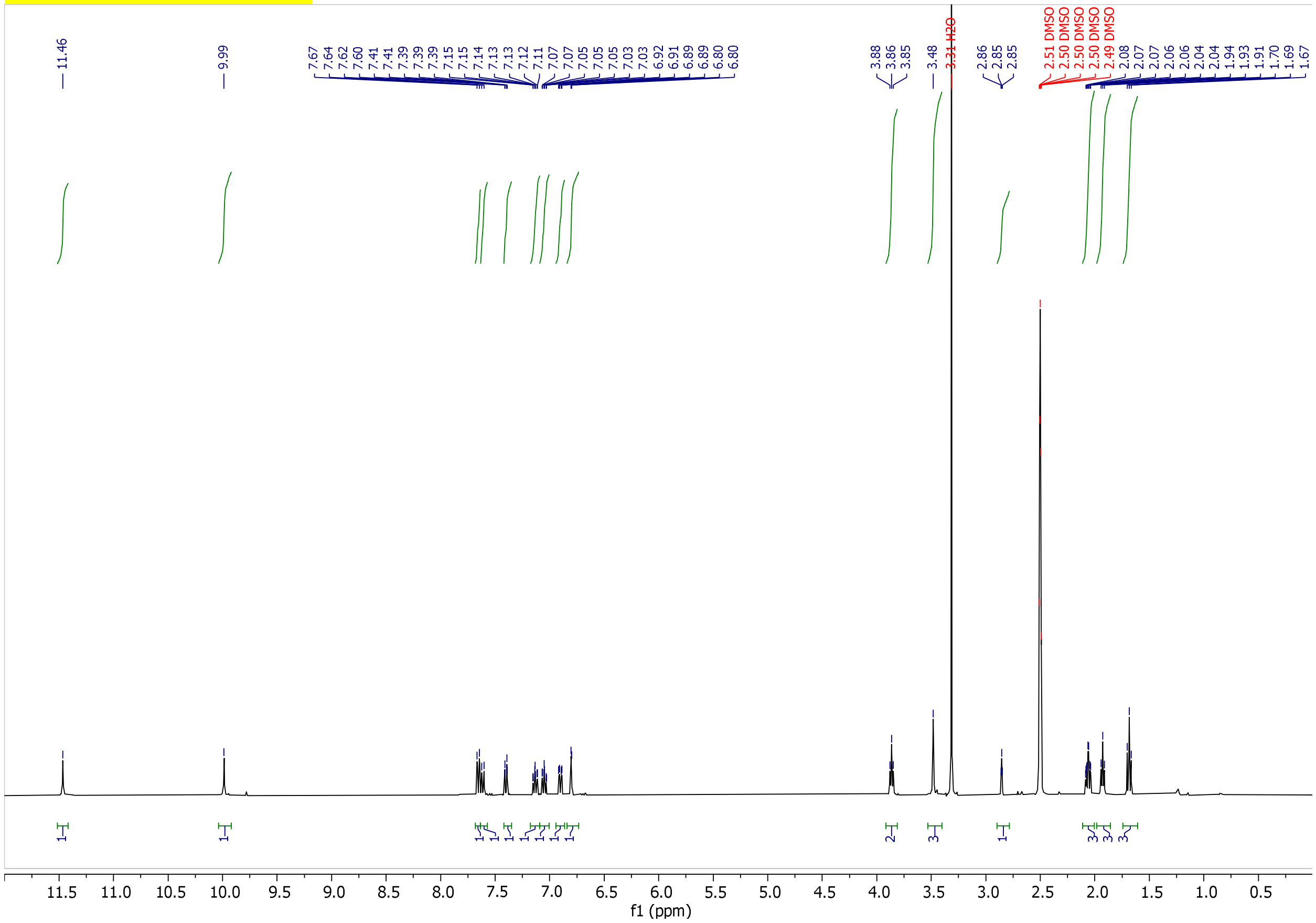

$^{13}$C NMR for KP-An-A-DA

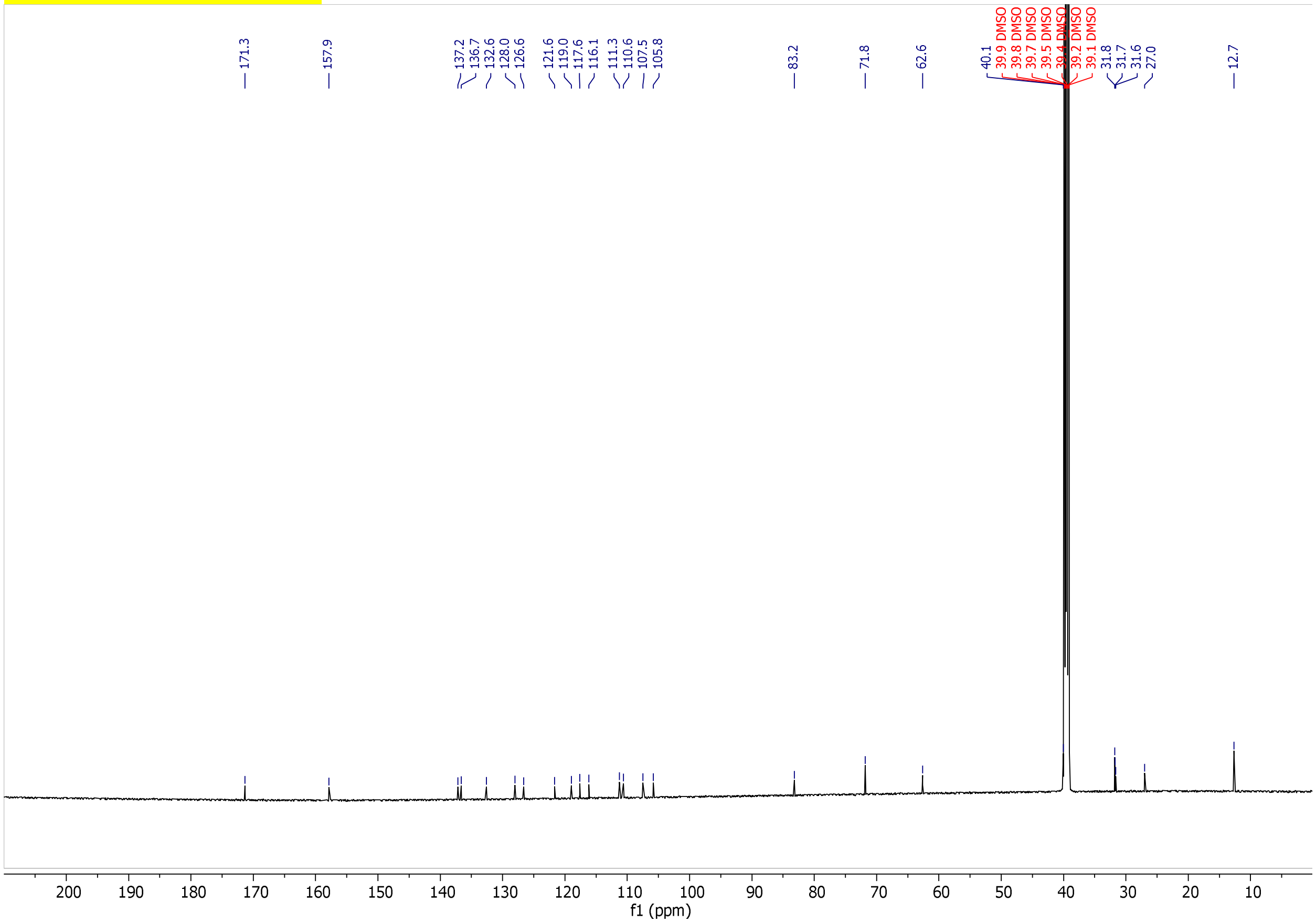

*$^{1}$H NMR for KP-AZ*

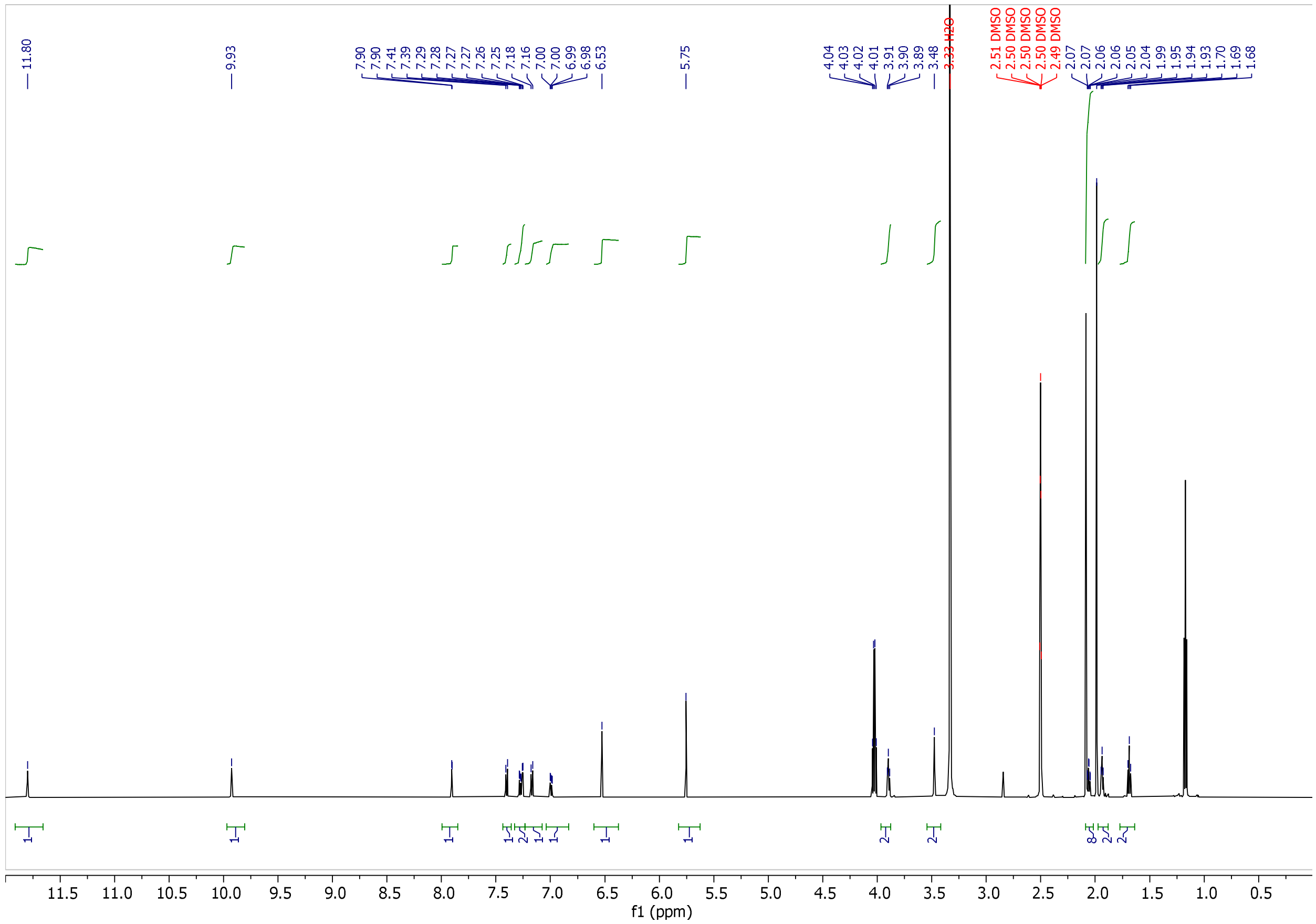

*$^{13}$C NMR for KP-AZ*

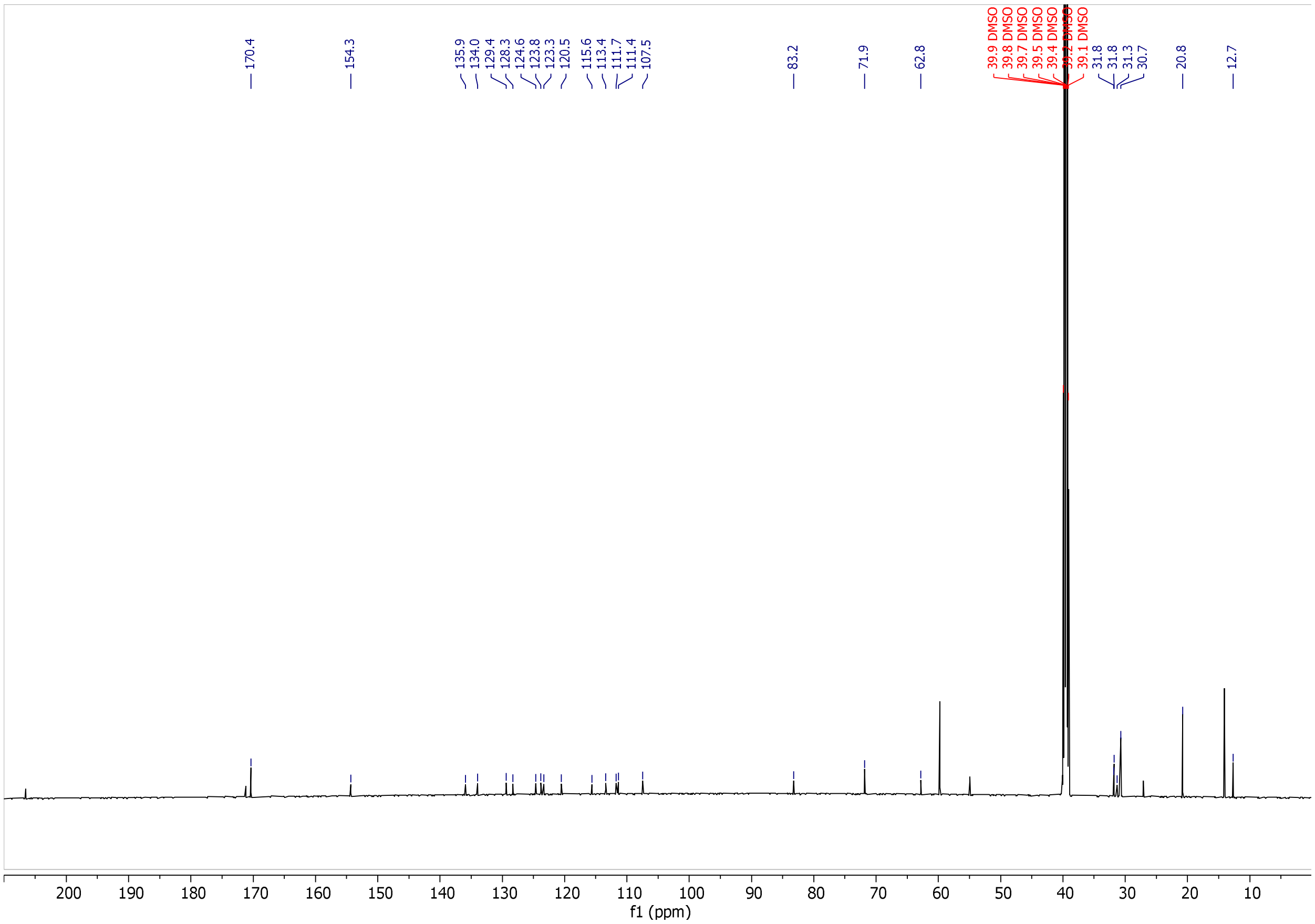

*¹H NMR for KP-An-A-AZ*

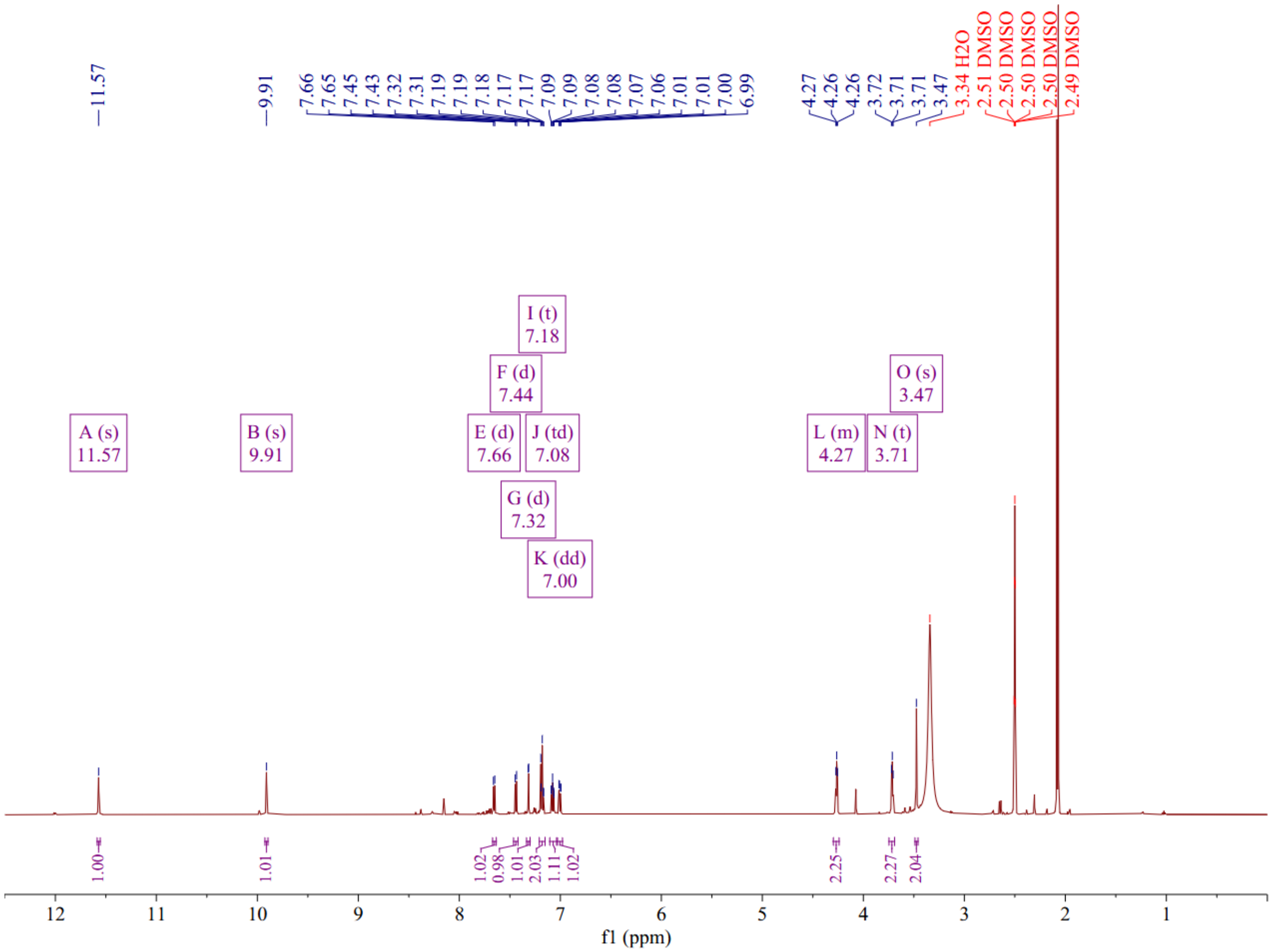

*$^{13}$C NMR for KP-An-A-AZ*

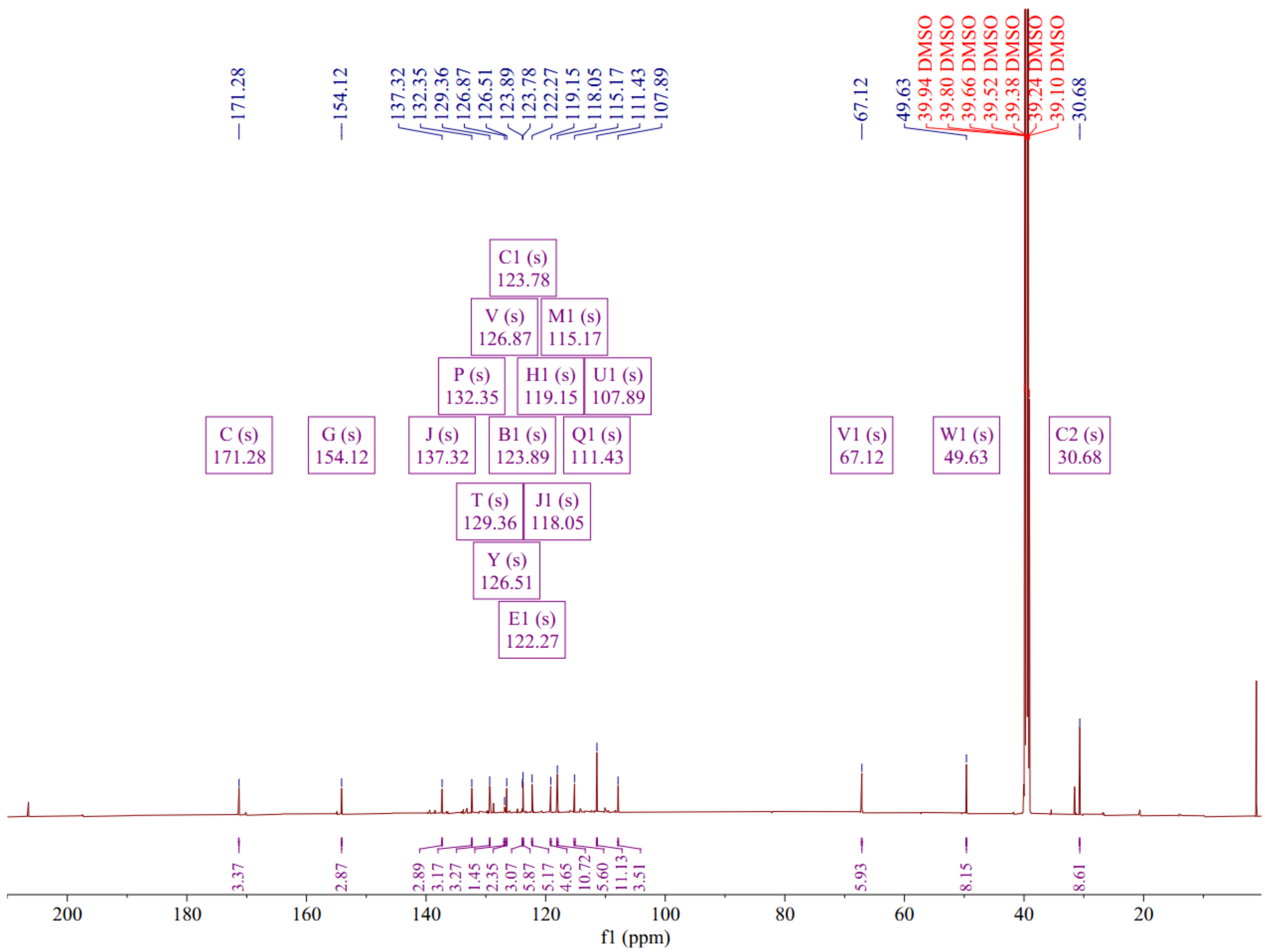

References


1 Huang, J. *et al.* Discovery and Optimization of Seven-Membered Lactam-Based Compounds to Phenocopy the Inhibition of the Aurora Kinase B. *ACS Med Chem Lett* **13**, 1091-1098 (2022). https://doi.org:10.1021/acsmedchemlett.2c00098

2 Duffey, M. O. *et al.* Discovery of a potent and orally bioavailable benzolactam-derived inhibitor of Polo-like kinase 1 (MLN0905). *J Med Chem* **55**, 197-208 (2012). https://doi.org:10.1021/jm2011172

3 Leost, M. *et al.* Paullones are potent inhibitors of glycogen synthase kinase-3beta and cyclin-dependent kinase 5/p25. *Eur J Biochem* **267**, 5983-5994 (2000). https://doi.org:10.1046/j.1432-1327.2000.01673.x

4 Lee, S. Y. *et al.* Small molecule-mediated up-regulation of microRNA targeting a key cell death modulator BNIP3 improves cardiac function following ischemic injury. *Sci Rep* **6**, 23472 (2016). https://doi.org:10.1038/srep23472

5 Yadav, N. et al. Tricyclic dihydrobenzoxazepine and tetracyclic indole derivatives can specifically target bacterial DNA ligases and can distinguish them from human DNA liase I. *Org Biomol Chem* **13**, 5475 (2015). 10.1039/c5ob00439j

6 Dang, T. *et al.* Hydrazo-CEST: Hydrazone-Dependent Chemical Exchange Saturation Transfer Magnetic Resonance Imaging Contrast Agents. *Chemistry* **24**, 9148-9156 (2018). https://doi.org:10.1002/chem.201801671

7 Yang, D. Z., Jing; Zhang, Shenqiu; Allen, Thaddeus; Shi, Qiong; Nimishetti, Naganna; Huang, Jian; Li, Hongmei; Yang, Chenglu. Preparation of fused azepine compounds and their use in the treatment of cancer. WO2022152097 (2022).

8 Wieking, K. *et al.* Synthesis of paullones with aminoalkyl side chains. *Arch Pharm (Weinheim)* **335**, 311-317 (2002). https://doi.org:10.1002/1521-4184(200209)335:7<311::AID-ARDP311>3.0.CO;2-F
